# A compendium of single extracellular vesicle flow cytometry

**DOI:** 10.1002/jev2.12299

**Published:** 2023-02-09

**Authors:** Joshua A. Welsh, Ger J. A. Arkesteijn, Michel Bremer, Michael Cimorelli, Françoise Dignat‐George, Bernd Giebel, André Görgens, An Hendrix, Martine Kuiper, Romaric Lacroix, Joanne Lannigan, Ton G. van Leeuwen, Estefanía Lozano‐Andrés, Shoaib Rao, Stéphane Robert, Leonie de Rond, Vera A. Tang, Tobias Tertel, Xiaomei Yan, Marca H. M. Wauben, John P. Nolan, Jennifer C. Jones, Rienk Nieuwland, Edwin van der Pol

**Affiliations:** ^1^ Translational Nanobiology Section, Laboratory of Pathology, Center for Cancer Research, National Cancer Institute National Institutes of Health Bethesda Maryland USA; ^2^ Department of Biomolecular Health Sciences Faculty of Veterinary Medicine Utrecht University Utrecht The Netherlands; ^3^ Institute for Transfusion Medicine University Hospital Essen, University of Duisburg‐Essen Essen Germany; ^4^ Vesicle Observation Center Amsterdam University Medical Centers Amsterdam The Netherlands; ^5^ Experimental Clinical Chemistry Amsterdam University Medical Centers Amsterdam The Netherlands; ^6^ Department of Chemical Engineering Drexel University Philadelphia Pennsylvania USA; ^7^ Aix Marseille Univ, INSERM, INRAE, C2VN, UFR de Pharmacie Marseille France; ^8^ Hematology and Vascular Biology Department CHU La Conception Assistance Publique‐Hôpitaux de Marseille Marseille France; ^9^ Clinical Research Center Department for Laboratory Medicine Karolinska Institutet Stockholm Sweden; ^10^ Evox Therapeutics Ltd Oxford UK; ^11^ Laboratory of Experimental Cancer Research Department of Human Structure and Repair Ghent University Ghent Belgium; ^12^ Biomedical Engineering & Physics Amsterdam University Medical Centers Amsterdam The Netherlands; ^13^ Dutch Metrology Institute VSL Delft The Netherlands; ^14^ Flow Cytometry Support Services LLC Arlington Virginia USA; ^15^ Amsterdam Cardiovascular Sciences Atherosclerosis and Ischemic Syndromes Amsterdam The Netherlands; ^16^ Cancer Center Amsterdam Imaging and Biomarkers Amsterdam The Netherlands; ^17^ Flow Cytometry & Virometry Core Facility, Faculty of Medicine, Department of Biochemistry, Microbiology, and Immunology University of Ottawa Ottawa Ontario Canada; ^18^ MOE Key Laboratory of Spectrochemical Analysis & Instrumentation Key Laboratory for Chemical Biology of Fujian Province Department of Chemical Biology College of Chemistry and Chemical Engineering Xiamen University Xiamen Fujian People's Republic of China; ^19^ Scintillon Institute San Diego California USA; ^20^ Cellarcus Biosciences San Diego California USA

**Keywords:** calibration, extracellular vesicles, flow cytometry, microparticles, MIFlowCyt‐EV, nanoparticles, standardization

## Abstract

Flow cytometry (FCM) offers a multiparametric technology capable of characterizing single extracellular vesicles (EVs). However, most flow cytometers are designed to detect cells, which are larger than EVs. Whereas cells exceed the background noise, signals originating from EVs partly overlap with the background noise, thereby making EVs more difficult to detect than cells. This technical mismatch together with complexity of EV‐containing fluids causes limitations and challenges with conducting, interpreting and reproducing EV FCM experiments. To address and overcome these challenges, researchers from the International Society for Extracellular Vesicles (ISEV), International Society for Advancement of Cytometry (ISAC), and the International Society on Thrombosis and Haemostasis (ISTH) joined forces and initiated the EV FCM working group.

To improve the interpretation, reporting, and reproducibility of future EV FCM data, the EV FCM working group published an ISEV position manuscript outlining a framework of minimum information that should be reported about an FCM experiment on single EVs (MIFlowCyt‐EV). However, the framework contains limited background information. Therefore, the goal of this compendium is to provide the background information necessary to design and conduct reproducible EV FCM experiments. This compendium contains background information on EVs, the interaction between light and EVs, FCM hardware, experimental design and preanalytical procedures, sample preparation, assay controls, instrument data acquisition and calibration, EV characterization, and data reporting. Although this compendium focuses on EVs, many concepts and explanations could also be applied to FCM detection of other particles within the EV size range, such as bacteria, lipoprotein particles, milk fat globules, and viruses.

## OUTLINE

1 Introduction 4

2 Background 5

2.1 Extracellular vesicle samples 5

2.1.1 Physicochemical properties of extracellular vesicles 5

2.1.2 Extracellular vesicle environment 18

2.2 Interaction between light and particles 19

2.2.1 Light 19

2.2.2 Fluorescence 20

2.2.2.1 Brightness 21

2.2.2.2 Quenching 22

2.2.2.3 Photon saturation and photobleaching 22

2.2.3 Light scattering 23

2.2.3.1 Refractive index 23

2.2.3.2 Scattering cross section 24

2.2.3.3 Rayleigh scattering 25

2.2.3.4 Mie theory 26

2.3 Flow cytometry 27

2.3.1 Fluidics 27

2.3.1.1 Sorter fluidics 28

2.3.2 Optics 29

2.3.2.1 Flow cell 29

2.3.2.2 Illumination optics 29

2.3.2.3 Detection optics 30

2.3.3 Electronics 30

2.3.3.1 Analog to digital converter 30

2.3.3.2 Differentiating optical signals from background noise 31

2.3.3.3 Deriving pulse statistics from optical signals 31

2.3.3.4 Coincidence and swarm detection 32

2.3.3.5 Dynamic range, resolution and scaling 32

2.4 Alternative flow cytometer implementations 33

2.4.1 Flow cytometry sorters 33

2.4.2 Imaging flow cytometers 34

2.4.2.1 Signal processing 34

2.4.3 Spectral flow cytometers 35

2.5 Flow cytometer characterization and calibration 35

3 Experimental design and pre‐analytical procedures 36

3.1 Experimental design 36

3.2 Pre‐analytical procedures 36

3.2.1 Ultracentrifugation 37

3.2.2 Density gradient centrifugation 37

3.2.3 Size exclusion chromatography 37

3.2.4 Ultrafiltration 38

4 Sample preparation 38

4.1 Fluorescent staining 39

4.1.1 Immunofluorescence staining 38

4.1.1.1 Direct and indirect immunofluorescence staining 39

4.1.1.2 Antibody alternatives 40

4.1.1.3 Fluorophore selection 40

4.1.1.4 Types of fluorophore 40

4.1.1.5 Tandem dyes 41

4.1.1.6 New fluorophore developments for EV FCM 41

4.1.1.7 Other fluorophore considerations 41

4.1.2 Generic fluorescent staining 42

4.1.2.1 Membrane and amine reactive dyes 42

4.1.2.2 Nucleic acid stains 42

4.1.3 Genetic expression of fluorescent proteins 42

4.2 Staining procedure 43

4.2.1 Staining conditions, considerations and procedure 43

4.2.1.1 Removal of staining reagent aggregates 43

4.2.1.2 Estimation of EV concentration 43

4.2.1.3 Titration of staining reagents 43

4.2.1.4 Optimization of incubation time and temperature 44

4.2.1.5 Reduction of unbound reagents 44

4.3 Detection of stained EVs by flow cytometry 44

5 Assay controls 45

5.1 Buffer‐only controls 46

5.1.1 Procedure 47

5.2 Buffer with reagents controls 47

5.2.1 Procedure 47

5.3 Unstained controls 47

5.3.1 Procedure 48

5.4 Isotype controls 48

5.4.1 Procedure 48

5.5 Fluorescence‐minus‐one and single‐stained controls 48

5.5.1 Procedure 49

5.6 Procedural controls 49

5.6.1 Procedure 49

5.7 Serial dilution controls 49

5.7.1 Procedure 50

5.8 Detergent treatment controls 50

5.8.1 Procedure 51

6 Instrument data acquisition and calibration 51

6.1 Trigger detector and threshold 51

6.1.1 Selecting the trigger detector(s) 51

6.1.2 Selecting the trigger threshold 51

6.2 Sample volume determination and flow rate stability 53

6.2.1 Calibrated pump 53

6.2.2 Counting beads 53

6.2.3 Determine sample volume weight 53

6.2.4 Flow rate sensor 54

6.3 Calibration 55

6.3.1 Fluorescence calibration 55

6.3.1.1 Procedures 55

6.3.1.2 Limitations 57

6.3.1.3 Cross calibration using hard‐dyed beads 57

6.3.2 Light scatter calibration 57

6.3.2.1 Procedure 58

6.3.2.2 Limitations 59

7 Extracellular vesicle characterization 59

7.1 Diameter, surface area and volume approximation of extracellular vesicles 59

7.1.1 Procedures 60

7.1.2 Limitations 60

7.2 Refractive index approximation of extracellular vesicles 61

7.2.1 Procedures 61

7.2.2 Limitations 62

7.3 Antibody and antigen number approximation 62

8 Data reporting 62

8.1 EV number concentration 62

8.2 EV brightness 63

8.3 EV size distribution 63

9 Conclusions 64

Acknowledgements 64

References 65

## 1 INTRODUCTION

Extracellular vesicles (EVs) are the generic term for particles naturally released from the cell that are delimited by a lipid bilayer and cannot replicate (Théry et al., 2018). Consequently, EVs are present in fluids contacting cells. As cells and EVs interact continuously, applications of EVs include liquid biopsy biomarkers, therapeutic agents, and quality monitoring of ecosystems and food production (Biller et al., 2014; Cai et al., 2018; Khamsi, 2020; van der Pol et al., 2012). However, realization of EV applications is challenging because (1) the subcellular size of EVs hampers their detection and characterization (van der Pol et al., 2014; van der Pol et al., 2010; Vogel et al., 2021), and (2) EVs originating from different cell types co‐exist with non‐EV particles (Tian et al., 2020). Flow cytometry (FCM) offers a multiparametric technology capable of identifying single EVs and measuring their cellular origin.

**FIGURE 1:**
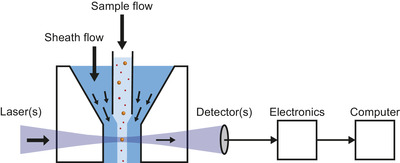
Schematic representation of a flow cytometer. Particles in a sample flow are hydrodynamically focused by a sheath flow and guided through the center of one or more focused lasers. Fluorescence and light scattering signals from particles are detected, processed by electronics and stored on a computer. Figure 7 shows a more detailed schematic of a flow cytometer.

Figure 1 shows that flow cytometers measure fluorescence and light scattering signals originating from thousands of single particles per second in a fluid stream (Shapiro, 2003). At optimal conditions, particles flow one by one through the centre of one or more focused laser beams. Next, the fluorescence and light scattering signals are collected, detected, processed by electronics and stored on a computer. The fluorescence signals can be used together with fluorescent staining to confirm the presence and quantify the number of biomolecules associated with EVs, including antigens and phospholipids (de Rond et al., 2019, 2018). Knowledge on the presence of antigens is typically used to establish the cellular origin of EVs. The light scattering signals can be used to derive the diameter and refractive index of EVs (de Rond et al., 2018; Konokhova et al., 2012; van der Pol et al., 2018). Per identified EV population, FCM can further be used to derive the number concentration (van der Pol et al., 2018) of the particles analyzed.

Most flow cytometers are designed to detect cells, which are larger than EVs. Whereas cells exceed the background noise, signals originating from EVs partly overlap with the background noise, thereby making EVs more difficult to detect than cells. This technical mismatch together with the complexity of EV‐containing fluids causes limitations and subsequent challenges with conducting, interpreting and reproducing EV FCM experiments. To address and overcome these challenges, researchers from the International Society for Extracellular Vesicles (ISEV), International Society for Advancement of Cytometry (ISAC), and the International Society on Thrombosis and Haemostasis (ISTH) joined forces and initiated the EV FCM working group (www.evflowcytometry.org).

While there are no gold standards for EV collection, handling, isolation, and detection, it has become clear that reporting all experimental variables will allow a better degree of reproducibility. This insight led to the publication of comprehensive literature on preanalytical variables to consider and report, such as the methodological guidelines to study EVs (Coumans et al., 2017), minimal information for studies of EVs (MISEV) (Lötvall et al., 2014; Théry et al., 2018), and transparent reporting and centralizing knowledge in EV research (EV‐TRACK) (Van Deun et al., 2017). To improve the interpretation, reporting, and reproducibility of future EV FCM data, the EV FCM working group published an ISEV position manuscript outlining a framework of minimum information that should be reported about an FCM experiment on single EVs (MIFlowCyt‐EV) (Welsh et al., 2020). As MIFlowCyt‐EV focuses on reporting, the framework contains limited background information. This compendium serves as a companion to MIFlowCyt‐EV to provide readers with background information and rationale for the elements within the reporting framework. The concepts covered in this current work, although EV‐centric, will be applicable to FCM measurements of all other particles within the EV size range.

This compendium starts with providing a background to EVs (Section 2.1), concepts around light (Section 2.2), and FCM hardware (Section 2.3) that are required to understand later chapters. After the background chapter, the chapters follow the structure of the MIFlowCyt‐EV framework and cover experimental design (Section 3.1) and preanalytical procedures (Section 3.2), sample preparation (Chapter 4), assay controls (Chapter 5), instrument data acquisition and calibration (Chapter 6), EV characterization (Chapter 7), and data reporting (Chapter 8). In addition, Table 1 highlights the main differences between FCM experiments on cells and EVs, Table 2 provides a list of frequent misconceptions, and Table 3 contains an index of terms and abbreviations used throughout this compendium.

**TABLE 1:** Main differences between flow cytometry (FCM) experiments on cells and extracellular vesicles (EVs). APC: allophycocyanin; CD: cluster of differentiation; FSC: forward scattered light; PE: phycoerythrin; SSC: side scattered light

Chapter	Topic	Cells	EVs
3. Experimental design & pre‐analytical procedures		Cells have a large surface area, express many different proteins and have a large cytosolic volume for intracellular staining compared to EVs. Therefore, it is feasible to detect more than 40 markers simultaneously.	Multiple factors limit the detection of multiple markers on EVs. These include limited surface area resulting in steric hindrance and flow cytometers typically having only 2–3 detectors capable of detecting <10 copies of a fluorophore.
	Fluorophore selection	In most cases the protein expression is high enough that modern flow cytometers can detect most fluorophores. Limitations in fluorophore detection mainly arise when larger fluorescent antibody panels are being built where there is significant spectral overlap and marker abundance needs to be accounted for.	EV detection using FCM requires the use of the brightest possible fluorophores, for example, PE, APC
4. Sample preparation	Fluorescent staining	When stained, cells are bright due to expressing high numbers of receptors, making it easy to stain a known number of receptors and remove unbound dye.	Due to limited numbers of receptors, stained EVs are dim, which hampers the detection of all EVs in a sample as well as antibody titration. In addition, the removal of unbound reagents typically results in dilution and loss of EVs and therefore is difficult, particularly for high‐throughput scenarios.
	Sample washing	Minor cell loss, because cells pellet down efficiently.	EVs may be discarded, because EVs are small and do not pellet down efficiently.
5. Assay controls	Buffer only controls	Cells exceed background noise.	EVs are close to and below the background noise.
	Buffer with reagents controls	Antibody aggregates have signals lower than cells.	Antibody aggregates may have signals similar to EVs.
	Unstained controls	Reagents do not affect the count rate of cells	Reagents may affect the count rate of EVs
	Isotype controls	Non‐specific binding to Fc receptors	Non‐specific binding to Fc receptors
	Single‐stained controls	Spectral spill over	Spectral spill over
	Procedural controls	Staining methods require specific processing steps after staining	Staining methods require specific processing steps after staining
	Serial dilution controls	Flow cytometer electronics are typically designed to detect and remove coincidence events. While two or three events may be coincidentally detected as doublets or triplets, this can usually be removed from analyses using parameter height, width, area comparisons.	Multiple (hundreds or more) EVs may be artefactually counted as one event (swarm detection)
	Detergent treatment controls	N/A	Unclear whether detected particles are envisioned EVs or other particles
6. Instrument data acquisition and calibration	FSC vs. SSC	Cells scatter majority of light in FSC direction. FSC is often used to determine relative size while SSC is a means of determining relative granularity of cells.	EV light scatter becomes more isotropic as they become smaller. FSC is less sensitive to resolve EVs than SSC on the majority of flow cytometers and SSC can be used in combination with Mie modelling to approximate the EV diameter.
	Trigger channel(s) and thresholds	Cells scatter the majority of light in FSC direction and exceed the limit of detection. The FSC threshold is sufficient for majority of cell applications	EVs are not fully detectable by flow cytometers. The best trigger channel and threshold for the maximum signal to noise ratio will depend upon the assay and will likely be an SSC or fluorescent trigger.
	Flow rate/volumetric quantification	Flow rate is generally not a concern for typically cellular analysis given that the input material is typically a diluted to a set concentration for staining for example,10^6^ cells/ml	Flow rate should be carefully considered for EV analysis and the impact of changing the flow rate to the sensitivity should be assessed. In many cases increasing the flow rate can decrease the sensitivity and increase the signal variation.
	Fluorescence calibration	Fluorescence calibration was developed to help determine lymphocyte epitope abundance, for example, CD4 epitopes	Fluorescence calibration is required to determine instrument sensitivity and enable comparisons across platforms with difference limits of detection. Fluorescence calibration is also used for EV characterization, such as epitope abundance.
	Light scatter calibration	Light scatter calibration not required due to the ease of cellular detection on commercial cytometers.	Light scatter calibration is required to determine instrument sensitivity and enable comparisons across platforms with difference limits of detection. Light scatter calibration is also used for EV characterization, such as diameter or refractive index approximation.

**TABLE 2:** List of frequent misconceptions regarding flow cytometry (FCM) experiments on extracellular vesicles (EVs). S/N: signal to noise ratio; RI: refractive index

Misconception	Background and good practice
Flow cytometers detect the entire size range of EVs	The dynamic range of flow cytometers allows detection of a limited size range only. The concentration of EVs should therefore be reported with the dynamic range of the detector(s) in standardized, comparable units. See section 2.1 for more information.
Flow cytometers are incapable of detecting EVs smaller than the wavelength of light	Particles with smaller dimensions than the wavelength of light do scatter light and may emit fluorescence, which can be detected with a flow cytometer if the signal exceeds the background noise.
The shortest illumination wavelength will provide the best EV light scatter sensitivity	While particles scatter light more efficiently at shorter illumination wavelengths, the detection of light has many variables that depend on the wavelength, such as the noise characteristics and quantum efficiency of the detector, the transparency of the flow cell, and the presence of stray light. The S/N determines which illumination wavelength results in the highest sensitivity. See section 2.3.2.2 for more information.
The scatter intensity of a polystyrene or silica bead is equivalent to a similar‐sized E	Light is scattered differently by particles with different refractive indices. Particles with higher refractive indices scatter more light. At an illumination wavelength of 488 nm, polystyrene (RI = 1.605) scatters light more efficiently than silica (RI ≈ 1.45), which scatters light more efficiently than EVs (RI ≈ 1.40). See Sections 2.2.3 and 6.3.2 for more information.
Using high‐speed and ultracentrifugation steps to isolate and concentrate EVs of different size (e.g., microvesicles and exosomes) upon FCM analyses	For many samples and staining procedures, it is not required to use high‐speed and ultracentrifugation steps to isolate and concentrate EVs upon FCM analysis. When the EV concentration is too low to provide statistically significant results, EVs should be concentrated. When the staining procedures introduce non‐EV particles that could be artifactually detected as EVs, such as micelles, EV samples require purification.

**TABLE 3:** List of terms and abbreviations used throughout the compendium. The meanings are not definitions but should be placed in the context of flow cytometry experiments on extracellular vesicles

Term used in manuscript	Abbreviation or symbol	Section(s)	Synonyms	Meaning
Absorption spectrum	ε(λ)	2.2.2.1–2.2.2.2		Fraction of incident light that is absorbed as a function of wavelength.
Allophycocyanin	APC	2.2.2.1		Protein isolated from red algae that exhibits fluorescence.
Amplitude (of a wave)		2.2.1		Measure of the change of wave in a single period.
Analog to digital converter	ADC	2.3.3.1		System that digitizes an analogue input signal, such as a current or voltage, by scaling it onto an axis with a fixed number of equally sized bins, which are called channel numbers.
Angular scattering distribution (of light)		2.2.3.4	Phase function	Distribution of the light scattering intensity as a function of the scattering angle.
Antibody bead capture	ABC	2.2.2.1		Beads bearing calibrated numbers of immunoglobulin binding molecules.
Antibody staining		4.2.1.4, 5.4, 5.4.1, 5.5.1	Antibody labeling, Immunostaining, immunofluorescence staining	Staining of antigens at the surface of EVs or cells with antibodies conjugated to fluorophores.
Antibodies		4.1		Reagent generally used to detect presence of specific protein epitopes.
Antigen‐binding fragment	Fab	4.1.1		Fragment of an antibody containing the antigen binding sites.
Aptamers		4.1.1.2		Oligonucleotide or peptide molecules that specifically bind to a molecular target, such as antigens.
Area (of a pulse)		2.3.3.3		Area under the curve of a signal pulse after baseline restoration [arb. unit]. See also Pulse statistics.
Arbitrary units	arb. Unit	2.2.3.2, 2.3.3.1, 2.3.3.2, 2.3.3.5, 2.5, 6.3, 6.3.1, 6.3.1.1		Relative unit of measurement that serves to compare multiple measurements performed in a similar environment.
Assay controls		2.1.2, 3.1, 4.3, 5, 5.6.1		Control experiments aiming to confirm that the detected signals originate from the intended particles.
Autofluorescence		2.2.2		Natural, intrinsic emission of light by a particle after absorbing light in the absence of fluorophores.
Avalanche photodiode	APD	2.3.2.3		Light detector made of a semiconductor that exploits the photoelectric effect to convert light into electricity.
Background noise		2.3.3.2, 4.3, 5.1, 6.1, 6.1.2		Unwanted offset and fluctuation of a signal measured in the absence of a particle and associated with electronic, fluidic, and optical noise.
Brightness (of an extracellular vesicle)		8.2		Measured fluorescence or light scattering intensity associated with an extracellular vesicle.
Brightness (of a fluorophore)	*B*	2.2.2.1		Efficiency of a fluorophore to emit light upon illumination $\left[m^{-1}\cdot M^{-1}\right]$
Buffer with reagents controls		5.2, 6.1.2		Assay control wherein fluorescent staining reagents in buffer are measured to quantify the contribution of unbound reagents to the total counts of particles in a sample.
Buffer‐only controls		5.1, 6.1.2		Assay control wherein the dilution buffer is measured to confirm that the buffer and flow cytometer are clean and to quantify the contribution of background noise.
Calibration		2.5, 6.3, 6.3.2		Measurement of a reference material that can be used to relate the arbitrary units of measurement to standard units, preferably with a known uncertainty.
Charges		2.2.1, 2.2.3		Electric charges, such as the electrons and protons of a molecule.
Coincidence (detection)		2.3.3.4		Stochastic process during which two or a few particles are simultaneously illuminated and detected.
Collisional quenching		2.2.2.2		Type of dynamic quenching, wherein an excited donor molecule experiences contact with an acceptor molecule that facilitates non‐radiative transitions to the ground state. See also Dynamic quenching.
Density gradient centrifugation		3.2.2		Method to purify extracellular vesicles that follows the principle of sedimentation by centrifugal force, where particle separation is based on differences in density.
Detergent treatment controls		5.8		Assay control to differentiate detergent‐sensitive membrane‐enclosed particles, such as extracellular vesicles, from detergent‐resistant particles.
Dynamic range		2.3.3.5, 2.5, 6.1.2, 6.3.1		Ratio between the upper and lower limit of detection.
Dynamic quenching		2.2.2.2		Type of quenching, wherein a donor molecule absorbs a photon and becomes excited, but in contrast to fluorescence the donor molecule transfers the energy to an acceptor molecule and therefore relaxes to the ground state without emission of a photon. See also Quenching.
Electronic abort		2.3.3.3		Method of a flow cytometer to abort and register coincidence events.
Electronic noise		2.3.3.2		Unwanted offset and fluctuation of a signal associated to the electronics, typically originating from dark current of the detector and thermal noise.
Electronics		2.3.3		One of the three basis systems of a flow cytometer, responsible for processing the electronic signals originating from the detectors. See also Fluidics and Optics.
Electromagnetic radiation		2.2.1	Electromagnetic wave	Waves made up by coupled oscillations of electric and magnetic fields originating from accelerating or oscillating charges, such as the electrons and protons of a molecule.
Effective refractive index		2.2.3.1	Solid particle equivalent refractive index	Refractive index of a solid particle, which scatters the same amount of light as a similar‐sized particle having a non‐homogeneously distributed refractive index
Effective scattering cross section	$\sigma_{\Omega }$	2.2.3.2		Hypothetical area [*m* ^2^] of a particle that incoming light must impinge in order to be scattered towards a lens with solid collection angle Ω. See also Scattering cross section.
Emission spectrum		2.2.2.2, 4.1.1.3		Relative fraction of incident light that is emitted by a fluorescence substance as a function of wavelength.
Event (signal)		2.3.3.2		Occurrence during which a signal exceeds the trigger threshold and is statistically characterized and registered by the electronics.
Extracellular vesicle	EV	2.1		Particle with a phospholipid membrane that is naturally released by a cell and does not contain a functional nucleus.
Flow cell		2.3.1, 2.3.1.1		Transparent cell that allows a liquid sample to flow through one or more laser beams.
Flow cytometry	FCM	1, 2.3	Traditional flow cytometry	Technology used to measure fluorescence and light scattering signals from single particles that are flowing through a focused light source, most commonly a laser beam.
Flow cytometry sorters		2.3.1.1, 2.4.1		Flow cytometer capable of sorting single particles in a liquid based on the measured fluorescence and light scattering signals. See also flow cytometry.
Fluidics noise		2.3.1		Unwanted offset and fluctuation of a signal associated to the fluidics, typically originating from particles in the sheath fluid.
Fluidics (of a flow cytometer)		2.3.1		One of the three basis systems of a flow cytometer, responsible for sample delivery and sample positioning at the laser intercept, and optionally for sorting of particles after detection. See also Electronics and Optics.
Fluorescein isothiocyanate	FITC	2.2.2.1		Fluorescent compound.
Fluorescence		6.3.1		Emission of light by a molecule after absorbing light.
Fluorescence resonance energy transfer	FRET	2.2.2.2		Type of resonance energy transfer, wherein the energy of an excited donor molecule is transferred from the donor to the acceptor, followed by the emission of photon. See also Resonance energy transfer.
Fluorescence lifetime		2.2.2, 2.2.2.2		Average time a molecule stays in the excited state before emitting a photon.
Fluorescence‐minus‐one controls		5.5		Assay control to determine the background fluorescence level in the absence of a fluorescent antibody conjugate, which is helpful to determine the fluorescence gate that differentiates between stained particles and unstained particles.
Fluorescent antibody conjugate		4.1.1	Antibody	Antibody conjugated with one or more fluorophores.
Fluorescent stain			Fluorescent dye, label, marker, probe, reporter, stain, tag	Fluorophores covalently bonded to a macromolecule, which in turn can bind to particles in a sample.
Fluorescent staining		2.1.1, 4.1, 4.1.2	Fluorescent labelling, probing, staining, tagging	Procedure to make particles fluorescent by adding a *fluorescent staining reagent* to a sample, which allows for the phenotypic analysis of particles through identification of specific components, such as lipids, proteins, DNA and RNA.
Fluorophore		2.1.1, 2.2.2, 2.3.2.2, 2.3.2.3, 2.4.3, 4.1, 4.1.1, 6.3.1.1	Fluorochrome	Chemical compound that exhibits fluorescence.
Fluorophore to protein ratio	F/P	4.1.1		Ratio of the number of fluorophores bound to a protein, typically an antibody.
Förster resonance energy transfer		2.2.2.2		See Fluorescence resonance energy transfer.
Frequency of light	*f*	2.2.1		Oscillation frequency [Hz] of an electromagnetic wave.
Height (of a pulse)		2.3.3.3	Peak	Amplitude of a signal pulse, typically after baseline restoration [arb. unit]. See also Pulse statistics.
Imaging flow cytometry		2.4.2		Technology used to image fluorescence and light scattering signals from single particles that are flowing through a focused light source onto an array of detectors.
Immunoglobulin G	IgG	2.1.1		Most common type of antibody found in the human blood circulation.
Interrogation point		2.3.1, 5.7		Volume where the sample flow is illuminated by the laser beam.
Irradiance (of light)	*E*	2.2.3.2		Power of light per unit area $\left[W\cdot m^{-2}\right]$. See also Power.
Isotype controls		5.4		Assay control, in which isotype control antibodies with a Fab region that recognizes irrelevant antigens are used to quantify the contribution of antibodies that do not bind via antibody‐antigen interaction but to Fc receptors.
Isotype specific fragment	Fc	2.1.1, 4.1.1		Fragment of an antibody that binds to isotype‐specific Fc receptors and that is identical in all antibodies of the same isotype.
Laser power		2.3.2.2		Amount of energy that a laser emits per second [*W*].
Jablonski diagram		2.2.2, 2.2.3		Diagram illustrating the levels of the energy states of a molecule and the transitions between them.
Jet‐in‐air		2.3.1.1		Fluid stream that is injected into open air by a nozzle and wherein particles are directly illuminated and detected.
Light		2		See Electromagnetic radiation.
Light scattering		2.2.3		Instantaneous re‐emission and redirection of light by an illuminated object
Limit of detection	LoD	2.1.1	Detection limit	Lowest signal that can be differentiated from the background noise with a sufficient degree of statistical significance.
Lipoprotein particles		2.1.2		
Median fluorescence intensity	MFI	4.3		Frequently used statistic to describe the fluorescence intensity distribution of a particle population.
Metrologically traceable		2.2.3.1, 6.2.2		Type of measurement or specification, wherein the measured or reported quantity values are related to a known reference through a chain of well‐documented calibrations, each contributing to the measurement uncertainty (Joint Committee for Guides in Metrology (JGCM) 2012).
Mie theory		2.2.3.4		Lorenz–Mie–Debye solution of Maxwell's equations, which describes the scattering of an electromagnetic plane wave by a homogeneous sphere, a core‐shell particle, or an infinite cylinder.
Milk fat globules		2.1.2		
Molar extinction coefficient	ε	2.2.2.1		Hypothetical area named the absorption cross section, which defines the fraction of incoming light that is absorbed by a mol of fluorophore $\left[m^{2}\cdot \mathrm{mo}l^{-1}\right]$. The units are also expressed as $\left[m^{-1}\cdot M^{-1}\right]$.
Molecules of equivalent soluble fluorochrome	MESF	4.3		Standard unit of fluorescence intensity, wherein 1 unit of MESF equals the fluorescence intensity of 1 unbound fluorochrome in solution under the same environmental conditions as the flow cytometry experiment.
Nanobody		4.1.1.2	Single‐domain antibody	Fragments of camelid antibodies that contain only a part of the Fab region.
Nanoparticle				Particle with a diameter between 1 and 100 nm.
Nanoparticle tracking analysis	NTA	2.4.2		Optical technology to measure the size distribution of particles in solution by tracking their Brownian motion.
Non‐EV particle		2.1.2		Particle not being an extracellular vesicle.
Nucleic acid stains			RNA, DNA stain	Fluorescent reagents that bind to nucleic acids in DNA and/or RNA.
Number concentration		1, 8.1	Concentration	The number of particles per volume of liquid $\left[mL^{-1}\right]$.
Numerical aperture	NA	2.3.2.3		Dimensionless number that characterizes the solid angle over which a lens can collect or emit light.
Optical noise		2.3.3.2		Unwanted offset and fluctuation of a signal associated to the optics, typically originating from black body radiation, fluorescence and Raman scattering of the buffer and/or sheath fluid, and stray light.
Optical signals		2.3.2, 2.3.3		Fluorescence and light scattering signals originating from illuminated particles.
Optics (of a flow cytometer)		2.3.2		One of the three basis systems of a flow cytometer, responsible for illuminating particles and collecting and propagating optical signals generated by the particle towards the fluorescence and light scatter detectors. See also Electronics and Fluidics.
Paul Karl Horan dye	PKH dye	4.1.2.1		Type of generic fluorescent stain that labels lipids.
Phospholipid membrane		1	Phospholipid bilayer, lipid bilayer	4 nm to 7 nm thick membrane made of two layers of lipid molecules
Phosphate buffered saline	PBS	2.2.2.1, 2.3.1		Buffer solution commonly used in biological research containing disodium hydrogen phosphate, sodium chloride, potassium chloride and potassium dihydrogen phosphate.
Photon		2.2.1, 2.2.2, 2.2.2.1, 2.3.2.3		Massless particle and smallest amount, also called quantum, of electromagnetic radiation.
Photobleaching		2.2.2.3		Irreversible modification of the chemical structure of a fluorophore by light, which renders the fluorophore non‐fluorescent.
Photodiode		2.3.2.3		Light detector that converts light into an electric current without intrinsic amplification.
Photon saturation		2.2.2.3		State of an illuminated fluorophore, wherein the frequency of the absorption and emission cycles is determined by the fluorescence lifetime, thereby limiting the emitted fluorescence intensity.
Photomultiplier tube		2.3.2.3		Light detector that multiplies the electric current produced by incident light in multiple dynode stages
Phycoerythrin	PE	2.1.1		Protein isolated from red algae that exhibits fluorescence.
Polarization (of a wave)		2.2.1		Geometric orientation of the oscillations of the field components of a wave.
Power (of light)	*P*	2.2.3.2		Amount of energy that light transfers per second [*W*].
Pre‐analytical procedures		3.2		Procedures that may affect a sample before the measurement, including collection, handling, storing and processing of a sample.
Pre‐analytical variables		3.2		Variables involved in the procedures that may affect a sample before the measurement.
Procedural controls		5.6		Assay control to identify potential artifacts due to the combination of certain reagents and EV purification methods.
Propagation direction (of a wave)		2.2.1		Direction where a wave is moving towards.
Pulse statistics		2.3.3.3	Summary statistics	Statistical summary of the signal pulse generated during an event, such as the area, height and width of a pulse.
Quantum dot	QD	4.1.1.4		Nanocrystals of a fluorescent semiconductor material that typically range 10–20 nm in diameter.
Quantum yield		2.2.2.1		Ratio between the number of emitted photons and the number of absorbed photons.
Quenching		2.2.2.2, 5.5, 5.5.1		Process induced by molecular interactions between a fluorophore and a surrounding molecule that decreases the quantum yield of a fluorophore.
Rainbow beads		6.3.1.3		Mixture of hard‐dyed beads impregnated with multiple fluorophores that excite and emit across the full spectral range used in flow cytometry.
Rayleigh scattering		2.2.3.3		Scattering of light by particles that are smaller than ∼1/10^th^ the illumination wavelength.
Reference material		2.5		Sufficiently homogeneous and stable material with specified properties that can be used to perform a calibration, that is, to relate the arbitrary units of measurement to standard units.
Reference particles		2.2.3.1, 2.2.3.4, 6.2	Beads	Type of reference material that contains homogeneous particles with specified properties, such as the fluorescence brightness, number concentration, mean diameter and/or refractive index, in solution.
Refractive index	*n*	2.1.1, 2.2.3.1		Physical property of a material, defined as the speed of light in vacuum relative to that in the material.
Resistive pulse sensing	RPS	4.2.1.2		Technology to measure the concentration and size distribution of particles in solution based on the Coulter principle (Cimorelli et al., 2021; Coumans et al., 2014).
Resolution		2.3.3.5		Ability to distinguish two different signal levels.
Resonance energy transfer		2.2.2.2		Type of dynamic quenching, wherein the energy of an excited donor molecule is transferred from the donor to the acceptor, followed by either non‐radiative transition to the ground state or emission of photon. See also Dynamic quenching.
Sample flow		2.3.1	Core stream	Laminar flow containing the sample, which is typically injected into the sheath flow and hydrodynamically focused into the flow cell of a flow cytometer.
Scattering cross section	$\sigma_{s}$	2.2.3.2		Hypothetical area [*m* ^2^] of a particle that incoming light must impinge in order to be scattered, thereby determining how efficiently a particle scatters light.
Self‐quenching		2.2.2.2		Type of resonance energy transfer, wherein the energy of an excited donor molecule is transferred to the same type of donor molecule, followed by a non‐radiative transition. See also Resonance energy transfer.
Sensitivity		2.1.1, 2.2.2.3, 2.3.2.2, 2.3.2.3, 2.4.1, 2.4.2, 2.5, 3.2.1, 4.3, 5.1.1, 5.2.1, 6.1.2, 6.3.1, 6.3.2, 8.1		Ability to detect weak signals, such as the ability to detect extracellular vesicles.
Serial dilution controls		5.7		Assay control aiming to finding the lowest sample dilution and highest count rate without the occurrence of swarm detection.
Sheath flow		2.3.1		Main laminar flow of liquid, typically water or phosphate buffered saline, running through the flow cell of a flow cytometer.
Shot noise		2.3.3.2	Photon noise, Poisson noise	Randomness of electronic and optical signals originating from the discrete nature of light (photons) and electricity (electrons).
Signal to noise ratio	S/N	2.3.2.3		Ratio of the level of a signal to the level of the background noise.
Single‐stained controls		5		Assay control, wherein a sample is measured in the presence of one fluorescent staining reagent to validate the compensation of spectral spillover and to identify potential confounding factors from fluorescent staining.
Size distribution		2.1.1, 4.3, 6.3.2.2, 7.1, 8.3	Particle size distribution	Relative number or relative number concentration of particle sizes present in a sample.
Size‐exclusion chromatography	SEC	3.2.3		Chromatography method to fractionate particles in solution based on size.
Spectral flow cytometry		2.4.3		Technology used to measure light scattering signals and the emission spectrum of fluorescence signals from single particles that are flowing through a focused light source.
Spectral spillover		4.1.1.3, 5.5, 5.5.1		Fluorescence light emitted by one type of fluorophore is picked up by the detector associated with the detection of another type of fluorophore, because the two different types of fluorophores have an overlapping emission spectrum.
Spectral unmixing		2.4.3		Mathematical procedure in which a measured emission spectrum, consisting of a mixture of overlapping spectra originating from different types of fluorophores, is decomposed into the spectra of the individual types of fluorophores.
Speed of light	*c*	2.2.1		Universal physical constant that is equal to 2.98 · 10^8^ m s^−1^.
Standard units		2.1.1, 2.2.2.1, 2.3.3.5, 2.5, 6.1.2, 6.3, 6.3.2.2, 8		Standardized units used for consistent measurement, such as the number of fluorescent molecules, the number of photons, the meter, and cubic meters.
Static quenching		2.2.2.2		Type of quenching, wherein a donor molecule forms a non‐fluorescent complex with an acceptor molecule in the ground state, which typically results in a change of the absorption spectrum of the donor molecule. See also Quenching.
Swarm detection		2.3.3.4, 5.7		Special form of coincidence detection, wherein multiple (hundreds or more) particles at or below the lower limit of detection are continuously and simultaneously illuminated and artefactually detected as single particles.
Time delay integration charge‐coupled devices	TDI‐CCD	2.4.2		Array of detectors designed to follow moving objects throughout the entire field‐of‐view before reading out the signals, which gives the ability to detect moving objects at low light intensities.
Total scattering cross section	$\sigma_{s}$	2.2.3.2, 2.2.3.4		Hypothetical area [*m* ^2^] of a particle that incoming light must impinge in order to be scattered into all directions. See also Scattering cross section.
Trigger threshold		2.3.3.2, 5.1.1, 5.2.1, 6.1		A predefined threshold filtering the signal of one or more detectors in order to record only those events whose signals exceed the threshold.
Ultracentrifugation		3.2.1		Method to concentrate extracellular vesicles that follows the principle of sedimentation by centrifugal force, where particle separation is based on differences in size and density.
Ultrafiltration		3.2.4		Method to fractionate particles in solution by size using a filter with pore sizes targeting proteins.
Unbound reagents		3.1, 3.2.4, 4.2.1.5, 5.2		Particles from a fluorescent staining reagent, such as proteins and fluorophores, that did not interact with EVs during fluorescent staining or reagents added prior to staining.
Unstained controls		5.3, 6.1.2		Assay control wherein a diluted sample is measured to determine the fluorescence level of unstained particles and to provide a reference for the number of events detected without reagents.
Wavelength	λ	2.2.1, 2.3.2.2		Distance over which the shape of a wave repeats.
Width (of a pulse)		2.3.3.3		Time interval during which a signal exceeds the trigger threshold [arb. unit]. See also Pulse statistics.

## 2 BACKGROUND

This chapter contains background information on EV FCM and starts with providing a physical description of EVs and EV samples (Section 2.1) and the interaction between light and EVs and other particles (Section 2.2). Next, the working principles of FCM in general (Section 2.3) and of FCM sorters, imaging flow cytometers, and spectroscopic flow cytometers (Section 2.4) are discussed.

### 2.1 Extracellular vesicle samples

#### 2.1.1 Physicochemical properties of extracellular vesicles

**FIGURE 2:**
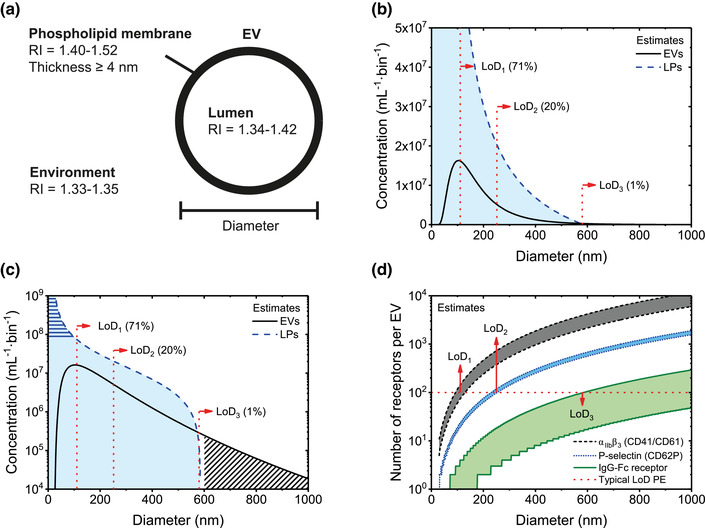
Physicochemical properties of extracellular vesicles (EVs) and their environment affecting flow cytometry measurements. (A) Schematic of an EV, which contains a lumen enclosed by a phospholipid membrane. The phospholipid membrane contains lipids and proteins, has a presumed refractive index (RI) of 1.40–1.52 (Ducharme et al., 1990; Horvath et al., 2003; Kienle et al., 2014; van Manen et al., 2008), and has a thickness of 4 nm or more depending on its composition (Arraud et al., 2014; Lewis & Engelman, 1983; Mitra et al., 2004). The lumen may contain DNA, lipids, organelles, proteins, RNA, and soluble molecules and has a presumed RI of 1.34–142 (Brunsting & Mullaney, 1974; Curl et al., 2005; Ghosh et al., 2006; Maltsev et al., 2011; Valkenburg & Woldringh, 1984; van Manen et al., 2008). The environment typically is phosphate‐buffered saline and has a RI close to water. In addition, the environment may contain DNA, non‐EV particles, proteins, RNA and soluble molecules. (B) Estimated size distribution of EVs (solid line) and lipoprotein particles (LPs; dotted line) in human blood plasma, which will be used throughout the manuscript as a model sample to explain typical problems involved in EV flow cytometry. The bin width is 1 nm. Due to the broad size distribution of EVs, the lower LoD (dotted lines) of different flow cytometers determines the totally measured EV concentration. The number in brackets indicates the percentage of detected EVs by a flow cytometer with given lower LoD. (C) The same size distributions as in panel B but plotted with a logarithmic vertical scale, revealing that the concentration of lipoprotein particles rapidly increases with decreasing diameter (horizontal stripes) and that the EV concentration decreases several orders of magnitude with increasing diameter (diagonal stripes). (D) Estimated number of αIIbβ3 (CD41/CD61) antigens, P‐selectin (CD62P) antigens, and IgG‐Fc receptors per EV versus the diameter of EVs in blood plasma, assuming that the number of receptors increases quadratically with the diameter of EVs. The horizontal dashed line indicates an LoD of 100 phycoerythrin (PE) molecules. The smallest detectable diameter of EVs does not only depend on the sensitivity of the detector used, but also on the properties of EVs, such as the number of stained receptors.

By definition, an EV is a particle with a phospholipid membrane that is naturally released by a cell and does not contain a nucleus (Théry et al., 2018). Figure 2(A) shows a schematic representation of an EV. The phospholipid membrane of an EV has a typical thickness between 4 and 7 nm (Arraud et al., 2014; Lewis & Engelman, 1983; Mitra et al., 2004; Perissinotto et al., 2021), but may be thicker due to the presence of proteins (Palviainen et al., 2020). To facilitate detection and characterization of EVs by FCM, lipids and proteins present in or associated with the membrane can be fluorescently stained (Section 4.1). Depending on the membrane composition, proteins can diffuse freely within the membrane (Vorselen et al., 2018). The membrane composition also determines the membrane refractive index, which in turn affects how efficiently the membrane scatters light (Section 2.2.3.1). Assuming that EVs and cells have a similar membrane composition, the refractive index of the membrane of EVs ranges from 1.40 to 1.52 (Ducharme et al., 1990; Horvath et al., 2003; Kienle et al., 2014; van Manen et al., 2008).

EVs contain an intraluminal region, composed of water and proteins, along with other biomolecules, including DNA, ions, lipids, and RNA. Comparable to the membrane, EV cargo can also be fluorescently labelled. The cargo of an EV determines its luminal refractive index, which in turn affect how efficiently the intravesicular lumen scatters light (Section 2.2.3.1). Assuming that the lumen of cells and EVs have a similar chemical composition, the refractive index of the lumen of EVs ranges from 1.34 to 1.42 (Brunsting & Mullaney, 1974; Curl et al., 2005; Ghosh et al., 2006; Maltsev et al., 2011; Valkenburg & Woldringh, 1984; van Manen et al., 2008). The relation between the refractive index distribution of an EV and the intensity of scattered light will be detailed in Section 2.2.3.4.

EVs are smaller than their cell of origin and therefore the size of EVs is an important property in differentiating EVs from cells. Size distributions of EVs have three general properties. First, EVs are polydisperse, meaning that EVs differ in size. Most fluids probably have a continuum of EV sizes that range from the smallest EVs to the smallest cells. Second, EVs have a minimum size depending on their composition. As a reference, the smallest EVs in human blood plasma have a diameter between 30 and 50 nm (Arraud et al., 2014; Brisson et al., 2017). Third, the size distribution of EVs typically has a maximum below 200 nm, which implies that above 200 nm the concentration of EVs decreases with increasing diameter. In human blood plasma, determination of EV morphology and size by cryo‐electron microscopy (cryo‐EM) indicates that more than 95% of the EVs are spherical and smaller than 500 nm, whereas rare, larger EVs can have a tubular shape (Arraud et al., 2014).

Figure 2(B) shows an estimate of the size distribution of EVs (solid line) in plasma from healthy individuals based on cryo‐EM and FCM data (Arraud et al., 2014; Gasecka et al., 2020). Although the actual size distribution of EVs may differ from the size distribution in Figure 2(B), the estimated size distribution is useful to explain the challenges involved in the detection of EVs by FCM. Like other optical instruments, flow cytometers have a lower and upper limit of detection (LoD; Section 2.3.3.5) for each measured signal and for the measured concentration. The lower and upper LoD of the detectors define the smallest and largest signals that can be detected, and thus determine the size range of EVs that can be characterized. Due to the broad size distribution of EVs, the lower LoD will define the detected concentration of EVs. For example, a theoretical lower LoD of three different FCMs is shown in Figure 2(B). The lower LoD corresponds to EVs with a diameter of 110 nm, 250 nm, or 580 nm, and allow for the detection of 71%, 20%, and 1% of all EVs, respectively. In this example, the total concentration is 3·10^9^ EVs per ml.

Differences between flow cytometer LoDs partially explain why the reported concentrations of EVs originating from the same biological samples can differ by orders of magnitude (Gasecka et al., 2017). Recent reports of measured EV concentrations are higher than those in older literature, probably due to the development of more sensitive assays and flow cytometers (Berckmans et al., 2019; Gasecka et al., 2017). To enable reproducible measurements of EV concentrations, the MIFlowCyt‐EV framework recommends to report LoD in standard units (Welsh et al., 2020). In reality, however, the lower LoD is not an infinitely narrow line but a complex function depending on the background signal and efficiency of the detector (de Rond et al., 2020). Moreover, the LoD expressed in terms of EV diameter also depends on the refractive index distribution of EVs. Therefore, metrologically sound procedures to determine the LoD need to be developed, validated and introduced to the field.

Figure 2(C) shows the same EV size distribution as in Figure 2(B), but with a logarithmic vertical scale. Whereas in Figure 2(B) the EVs larger than 600 nm seem absent, Figure 2(C) shows that EVs larger than 600 nm are present, and that the EV concentration decreases several orders of magnitude with increasing diameter of EVs. Many flow cytometers have the capacity to detect the upper range of the EV size distribution, and due to the power‐law nature of the distribution of EVs, these instruments will measure a significantly lower concentration compared to more sensitive flow cytometers.

As most EVs are approximately spherical, geometry can be used to estimate the surface area and volume of an EV from the measured diameter. The surface area of EVs determine (1) the number of lipids and proteins present in the membrane, and (2) the number of fluorophores that can be stained per EV using fluorescent staining. Figure 2(D) shows an estimate of the number of integrin αII_b_β_3_ (CD41/CD61), the most abundant platelet receptor, P‐selectin (CD62P), a receptor reflecting platelet secretion, and isotype‐specific receptors (Fc) of immunoglobulin G (IgG) per EV versus the diameter of EVs in blood plasma. Assuming a constant number of receptors per surface area, the number of receptors increases quadratically with the diameter of EVs. Figure 2(D) also shows a lower LoD corresponding to the detection of 100 phycoerythrin (PE) molecules, which would allow the detection of EVs with (1) a diameter ≥110 nm when stained with αII_b_β_3_, (2) a diameter ≥250 nm when stained with P‐selectin, and (3) a diameter ≥580 nm when stained with IgG‐Fc. Thus, the smallest detectable diameter of EVs does not only depend on the sensitivity of the flow cytometer, but also on the properties of EVs, such as the density of antigens.

#### 2.1.2 Extracellular vesicle environment

In an EV FCM experiment, the environment of EVs is predominantly water (Figure 2A), which has a refractive index between 1.33 and 1.35 for illumination wavelengths typically used in FCM (Section 2.2.1). The environment of EVs may contain non‐EV particles, DNA, ions, proteins, and RNA. Especially non‐EV particles, such as lipoprotein particles and milk fat globules (de Rond et al., 2019; van der Pol et al., 2018; van Herwijnen et al., 2016), may overlap with EVs in size and density and even form complexes (Sódar et al., 2016), thereby making it impossible to isolate (Section 3.1) or discriminate all EVs from non‐EV particles.

Although individual particles smaller than EVs, including DNA, soluble molecules, proteins, and RNA are generally below the lower LoD of flow cytometers, they can still affect EV detection by (1) interacting with and binding to EVs, (2) forming aggregates, or (3) increasing the background noise level. The presence of particles with properties overlapping with EVs emphasizes the need for (1) assay controls (Chapter 5) to confirm that detected signals indeed originate from EVs, (2) a pan‐EV marker specifically staining all and only EVs (de Rond et al., 2019; de Rond et al., 2018), and (3) isolation methods with 100% recovery of exclusively EVs. Assay controls for EV FCM experiment are well‐established, but currently neither a pan‐EV marker nor a perfect isolation method exist.

To exemplify how non‐EV particles may interfere with detection of EVs, Figure 2(B) shows the overlap in size distributions between lipoprotein particles and EVs. As reliable reference intervals for lipoprotein particle concentrations or EV concentrations in blood plasma do not yet exist, the concentrations are an order of magnitude approximation. For reference, the total concentration of lipoprotein particles is in the order of 2·10^16^ particles per ml (Jeyarajah et al., 2006).

Figure 2(C) illustrates that for EV size distribution in blood plasma, the concentration of lipoprotein particles rapidly increases with decreasing diameter (Kuchinskiene & Carlson, 1982; Matyus et al., 2015). Current knowledge suggests that the total EV concentration in plasma is orders of magnitude lower than the concentration of lipoprotein particles, particularly lipoprotein particles with a diameter <100 nm. Thus, EVs in blood plasma are outnumbered by lipoprotein particles. Consequently, the smaller the particles a flow cytometer can detect, the higher the ratio of detected lipoprotein particles to EVs. As most EV samples are a complex mixture of components that often outnumber the EVs, assay controls (Chapter 5) are essential to confirm that the detected signals indeed originate from EVs.

### 2.2 Interaction between light and particles

To understand how light is used to detect particles with FCM, the next sections will define light, discuss its origin and most relevant properties, and explain how EVs and similar‐sized particles interact with light within the context of FCM.

#### 2.2.1 Light

**FIGURE 3:**
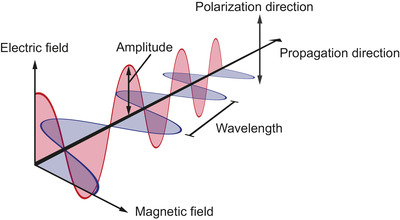
Schematic representation of light. Light is electromagnetic radiation, which are waves made up by coupled oscillations of electric (red) and magnetic (blue) fields. The primary properties of an electromagnetic wave are the amplitude, polarization direction, propagation direction, and wavelength.

In FCM, particles are mainly illuminated and detected using visible light, which is electromagnetic radiation with wavelengths between 400 and 700 nm in air. Electromagnetic radiation are waves made up by coupled oscillations of electric and magnetic fields. Electromagnetic radiation originates from accelerating or oscillating charges, such as the electrons and protons of a molecule. When the centre of a negatively charged cloud of electrons is shifted away from the centre of a positively charged cloud of protons, the molecule constitutes an electric dipole, which means that the molecule acts like an antenna. During this process, an electromagnetic wave is created. Figure 3 shows the primary properties of an electromagnetic wave, which are the amplitude, polarization, propagation direction, and wavelength.

The amplitude of an electromagnetic wave is relevant for particle detection, because the intensity of light scattered by a particle is proportional to the square of the amplitude of an electromagnetic wave. Polarization refers to the geometrical orientation of the electric and magnetic field components. For example, in Figure 3 the electric field component is oriented vertically. The propagation direction denotes where the wave is moving towards, and the wavelength is the distance over which the shape of the wave repeats. The speed of light in vacuum is a universal physical constant that is equal to 2.98 · 10^8^ m s^−1^. In a material, the speed of light is lower than in vacuum and determined by the refractive index (Section 2.2.3.1). Furthermore, the speed of light *c* and the wavelength λ determine the frequency of light *f* according to:

(1) $$f\;=\frac{c}{\lambda }\;\left[s^{-1}\right]$$

Thus, the lower the frequency of the oscillating charges and the emitted light, the longer the wavelength of the emitted light, and the lower the energy of the electromagnetic wave.

Light can be described as electromagnetic radiation, but also as a stream of massless particles named photons, which is relevant to understand the concept of fluorescence (Section 2.2.2). A photon is the smallest amount, also called quantum, of electromagnetic radiation.

#### 2.2.2 Fluorescence

Fluorescence is the emission of light by a molecule^1^ after absorbing light. Fluorescence is not instantaneous, but typically takes several nanoseconds (Lakowicz, 2006). In addition, energy is lost during absorption and fluorescence and therefore the emitted light has a longer wavelength than the incoming light.

**FIGURE 4:**
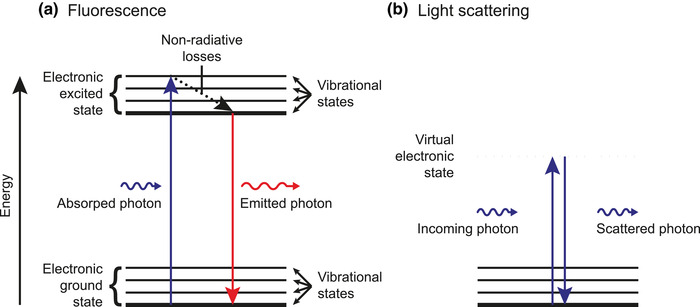
Jablonski diagrams of fluorescence and light scattering by a molecule. (A) The horizontal lines represent the levels of energy states of a molecule. The lowest energy level corresponds to the ground state (thick line). When the electrons are in the lowest possible orbital, the molecule is in the electronic ground state. Within the electronic ground state, the molecule can occupy different vibrational states with different energies, representing different types of periodic motions of the molecule. In the process of fluorescence, an incoming photon is absorbed and temporarily excites both the electronic and vibrational state of a molecule. Next, the molecule loses energy and therefore decays to the lowest vibrational state without radiative emission (dotted arrow). Finally, the molecule relaxes to the ground state under the emission of a photon. Due to the non‐radiative energy loss, the emitted photon has a lower energy and longer wavelength than the incoming photon. (B) In the process of light scattering, an incoming photon excites the molecule to a virtual electronic state (dashed line), which is instantaneously followed by relaxation of the molecule to the ground state and emission of a photon. The energy and wavelength of the incoming and scattered photon are the same.

To understand fluorescence, it is important to know that a molecule has different energy states, which depend on the orbital of the electron(s) and the vibrational motion of the molecule. To illustrate this, Figure 4(A) shows a Jablonski diagram depicting the fluorescence of light by a molecule. The horizontal lines represent the levels of energy states of a molecule. The lowest energy level corresponds to the ground state. When the electrons are in the lowest possible orbital, the molecule is in the electronic ground state. Within the electronic ground state, the molecule can occupy different vibrational states with different energies, representing different types of periodic motions of the molecule. In the process of fluorescence, an incoming photon is absorbed and temporarily excites both the electronic and vibrational state of a molecule, as shown in Figure 4(B).^2^ Around one picosecond after the absorption of a photon, the excess of vibrational energy is converted into non‐radiative energy, such as heat. Due to the non‐radiative loss of energy, a molecule decays to the vibrational ground state of the electronic excited state. Next, typically within several nanoseconds, the molecule relaxes to the ground state under the emission of a photon. Due to the non‐radiative energy loss, the emitted photon has a lower energy and thus a longer wavelength than the incoming photon. Once the molecule has returned to the ground state, the process of fluorescence can be repeated as long as the molecule remains intact. The average time a molecule stays in the excited state before emitting a photon is called the fluorescence lifetime and is typically several nanoseconds for organic compounds (Lakowicz, 2006).

In EV FCM, fluorescence is commonly employed by immunofluorescence staining, during which antibodies conjugated with fluorophores are used to detect EV‐associated antigens (Section 4.1.1). However, EVs and other biological particles also emit autofluorescence in the absence of fluorophores, which is intrinsic fluorescence by molecules in the particle. Biochemical molecules that contribute to autofluorescence include aromatic amino acids, collagen, elastin, flavins, lipo‐pigments, and nicotinamide adenine dinucleotide (Monici, 2005). To distinguish stained EVs from unstained particles, the fluorescence intensity of fluorophores that are bound to EVs should be higher than the autofluorescence intensity of unstained particles and, added to that, the background signals of the flow cytometer. The fluorescence intensity of stained EVs depends on the number of fluorophores and the brightness of the fluorophores. The brightness is an important property of fluorophores and is one of several factors that determine whether a stained EV can be detected.

##### 2.2.2.1 Brightness

The brightness of a fluorophore describes how efficiently a fluorophore emits light once illuminated. The common definition of the brightness *B* of fluorophores in suspension is:

(2) $$B\;=\;\varepsilon \left(\lambda ,n\right)\cdot \Phi \left(\lambda ,n\right)\;\left[m^{-1}\cdot M^{-1}\right]$$

where ε is the molar extinction coefficient, Φ the quantum yield, λ the illumination wavelength, and *n* the refractive index of the environment. Analogous to the concept of scattering cross section (Section 2.2.3.2), ε describes a hypothetical area named the absorption cross section, which defines the fraction of incoming light that is absorbed by a mol of fluorophore^3^. The term ε is therefore a measure of how efficient a fluorophore absorbs light. The quantum yield Φ describes the ratio between the number of emitted photons and the number of absorbed photons. When Φ is equal to 1, each absorbed photon results in the emission of photon. In practice, however, Φ is lower than 1 due to processes such as quenching (Section 2.2.2.2) (McCarthy, 2007). Both ε and Φ depend on λ and *n*. To optimize the brightness, λ should match the peak of the absorption spectrum ε(λ). In addition, ε and particularly Φ depend on the (bio) chemical environment, such as the composition and temperature of the medium and the close proximity of other fluorophores. Therefore, a fluorophore in water versus phosphate‐buffered saline (PBS), or a bound versus unbound fluorophore will differ in brightness. As an indication for the brightness of common fluorophores in PBS, allophycocyanin (APC) is 3 to 4‐fold brighter than fluorescein isothiocyanate (FITC) and PE is ∼10‐fold brighter than FITC when illuminated at their optimal excitation wavelength (Chattopadhyay et al., 2012; Dempsey et al., 2011).

Aside from the brightness of a fluorophore, many other factors will impact the measured fluorescence intensity, such as the fluorophore to protein ratio (F/P), photon saturation, photobleaching (Section 2.2.2.3), and the optical configuration of the flow cytometer. For example, when the F/P of APC is 1 and FITC is 4, the brightness per antibody becomes similar, assuming that each antibody is conjugated with the same number of fluorophore molecules. A useful method to compare brightness in practice is to purchase the same antibody clone conjugated to different fluorophores and use antibody bead capture (ABC) calibration (Sections 6.3.1.1 and 7.3). When selecting and evaluating different fluorophores, the measured fluorescence intensities should be compared in standard units, which can be assessed through calibration (Section 6.3.1).

##### 2.2.2.2 Quenching

Quenching is a process induced by molecular interactions between a fluorophore and a surrounding molecule that decreases the quantum yield of a fluorophore. Quenching therefore leads to a decrease in fluorescence intensity (Deka et al., 1996; Lakowicz, 2006) and is an undesired phenomenon. The fluorophore is named the donor, and the surrounding molecule the acceptor. The strength of the interaction between the donor and acceptor depends on their distance. The most relevant types of quenching for EV FCM are static and dynamic quenching.

In static quenching, the donor forms a non‐fluorescent complex with the acceptor in the ground state, which typically results in a change of the absorption spectrum of the donor (Lakowicz, 2006). For example, the fluorophore coumarin‐120 is statically quenched by the nucleotides uridine and deoxycytosine when excited at 386 nm (Seidel et al., 1996).

In dynamic quenching, the donor absorbs a photon and becomes excited, but in contrast to fluorescence the donor transfers the energy to the acceptor and therefore relaxes to the ground state without emission of a photon. Two types of dynamic quenching are collisional quenching and resonance energy transfer. Collisional quenching occurs when the excited donor experiences contact with an acceptor that facilitates non‐radiative transitions to the ground state. Collisional quencher molecules include amines, bromide, iodide, oxygen, and halogens (Lakowicz, 2006). Resonance energy transfer occurs when the donor and acceptor are in close (1–10 nm) proximity and when the emission spectrum of the donor overlaps with the absorption spectrum of the acceptor (SzöllHosi et al., 2006; Vogel et al., 2014). With resonance energy transfer, the energy is transferred from the donor to the acceptor, followed by either non‐radiative transition to the ground state or emission of photon. Resonance energy transfer followed by the emission of a photon is known as Förster or fluorescence resonance energy transfer (FRET). Resonance energy transfer between a donor and an acceptor of the same kind followed by a non‐radiative transition is known as self‐quenching.

As an example of self‐quenching in FCM, the quantum yield of FITC conjugated to an antibody can be reduced when more than four to six fluorophores are bound to a single antibody, or when the antibody density on the cell surface is sufficiently high (Deka et al., 1996). For FITC, “sufficiently high” is defined as an antibody density and F/P resulting in 1 fluorophore per ∼250 nm^2^ at the cell surface (Deka et al., 1996). FITC, however, can also be quenched by other fluorophores, such as R‐phycoerythrin, which are often combined in fluorescent antibody panels (Chapple et al., 1990).

The practical consequence of quenching is that the measured fluorescence intensity is no longer proportional to the number of bound antibodies. Therefore, experiments using fluorophores should be critically evaluated for quenching. The confirmation of quenching, however, requires specialized equipment to measure the absorption spectrum, the emission spectrum, and the fluorescence lifetime. Hitherto, experimental evidence of quenching for EVs is lacking. Whether quenching of fluorophores on EVs occurs, depends on the density of fluorophores, but counterintuitively not on the small dimensions of EVs. It is unlikely that the small dimensions of EVs increase the probability of quenching, because the smallest EVs have a diameter between 30 and 50 nm (Arraud et al., 2014; Brisson et al., 2017), which is substantially larger than the distances required for quenching (1–10 nm).

Generally, static quenching and collisional quenching can be reduced or prevented by selecting fluorophores that are known not to quench when taken into close proximity to each other and to molecules present at the surface of EVs. Self‐quenching can be limited by avoiding high fluorophore concentrations, which depend on the F/P and the antibody density at the surface of EVs. When self‐quenching is caused by a too high antibody density, this can be recognized by antibody titration (Section 4.2.1.3). In the case of self‐quenching, an increase of antibody concentration results in a decrease of the fluorescence intensity.

##### 2.2.2.3 Photon saturation and photobleaching

The emitted fluorescence intensity of a fluorophore depends on the brightness (Section 2.2.2.1), the fluorescence lifetime, and the susceptibility to photobleaching of the fluorophore. The fluorescence lifetime is typically several nanoseconds, whereas the typical illumination time in a flow cytometer is in the order of a μs. During the illumination time, fluorophores typically undergo hundreds of absorption and emission cycles^4^ (van den Engh & Farmer, 1992). The shorter the fluorescence lifetime and the faster a fluorophore is re‐excited, the higher the measured fluorescence intensity. Fast re‐excitation of a fluorophore can be achieved by increasing the illumination irradiance (Section 2.2.3.2). At illumination irradiances where the frequency of the absorption and emission cycles is determined by the fluorescence lifetime, a state of photon saturation is reached and a further increase of the fluorescence irradiance will not result in increased fluorescence (van den Engh & Farmer, 1992).

In practice, many types of fluorophores can only go through a finite number of absorption and emission cycles because light irreversibly modifies their chemical structure^5^. This process is called photobleaching and renders the fluorophore non‐fluorescent. Despite the short illumination time in FCM compared to for example light microscopy, photobleaching does occur in FCM (Pinkel et al., 1979). In contrast to photon saturation, which limits the effective illumination irradiance, photobleaching limits the total dose of photons that a fluorophore can convert into fluorescence (van den Engh & Farmer, 1992). Although the use of an illumination irradiance to reach photobleaching within the illumination time seems beneficial, this strategy may reduce the sensitivity. Whereas bleaching all fluorophores of a particle results in the maximum fluorescence signal, the required illumination irradiance may be so high that the fluorescence signal drops below the optical background noise. The optimal illumination irradiance is therefore not necessarily the maximum illumination irradiance, but instead depends on the chemical environment, fluorescence lifetime, illumination history, and susceptibility to photobleaching of the fluorophore.

Photon saturation and photobleaching are not necessarily a shortcoming, as they can be used to reduce the variation between fluorescence measurements. The illumination of most flow cytometers has a Gaussian irradiance profile (Section 2.3.2.2), which causes particles that pass at different positions through the laser beam to receive different illumination doses. At illumination irradiances below the level of photon saturation and photobleaching, there is a linear relationship between the illumination irradiance and the fluorescence intensity. Consequently, fluctuations of the particle position within the laser beam increase the variation in fluorescence measurements. However, at illumination irradiances that induce photon saturation and/or photobleaching, the fluorescence intensity becomes independent of the particle position, causing a reduction in the variation of fluorescence measurements (van den Engh & Farmer, 1992). Whether photon saturation and/or photobleaching can be used to reduce the variation in fluorescence measurements of EVs requires further investigation.

#### 2.2.3 Light scattering

Light scattering is the instantaneous re‐emission and redirection of light by an illuminated object, such as an EV. As explained in Section 2.2.1, light originates from oscillating charges. When light in turn impinges upon charges within a particle, these charges will start to oscillate as well. Consequently, the incoming light is re‐emitted, that is, scattered, into different directions. Figure 4(B) shows a Jablonski diagram depicting scattering of light by a molecule. An incoming photon excites the molecule from the ground state to a virtual electronic state. In contrast to fluorescence, the excitation is instantaneously followed by relaxation of the molecule to the ground state and emission of a photon. The wavelength of the incoming and scattered photon remains the same, because generally no energy is lost^6^.

Despite the small size of atoms relative to the wavelength of visible light, every single atom scatters light because atoms consist of charged particles. Thus, single proteins, non‐EV particles, and the smallest EVs all scatter light once illuminated. Scattered light may not only reveal the presence of an EV, but also provide information on the physical properties of the EV. Particularly for spherical particles in a liquid, light scattering is well‐understood and determined by the particle diameter, refractive index ratio between the particle and the medium, illumination irradiance, polarization direction, and wavelength of the light illuminating the particle.

##### 2.2.3.1 Refractive index

The refractive index (*n*) of a particle determines how efficiently a particle scatters light. Understanding the relation between the refractive index and light scattering (1) is essential to correctly interpret light scatter signals from EVs and reference particles, such as polystyrene beads, and (2) can be used to derive the diameter of EVs and standardize EV concentration measurements. The refractive index of a material is defined as the ratio of the speed of light in vacuum compared to the speed of light in the material. For example, the speed of light in air ($n_{air}=\;1.000$) is 34% higher than in water ($n_{water}=\;1.34$) (Daimon & Masumura, 2007). At the nanoscopic level, the refractive index depends on the density of molecules and on the tendency of charges to oscillate when exposed to light. According to the Lorentz‐Lorenz equation, the higher the density of molecules and/or the higher the tendency of charges to oscillate, the higher the refractive index, and the slower light travels through that material. At the macroscopic level, the refractive index affects the reflection and refraction of light. For example, from Snell's law and the Fresnel equations it is known that the higher the refractive index ratio between two materials, the stronger light refracts and reflects at the interface, respectively (Hecht, 2001). From the perspective of reflection and refraction, it is understandable that a higher refractive index difference between a particle and the medium results in more light being scattered. How exactly the refractive index affects the intensity of light scattered by EVs, will be explained in Section 2.2.3.4.

The refractive index is an intrinsic property that depends on the illumination wavelength. For most materials illuminated by visible light, the refractive index decreases as the illumination wavelength increases. A material that is commonly used to produce reference particles for the calibration of light scatter detectors of flow cytometers (Section 6.3.2) is polystyrene. Polystyrene has a refractive index of 1.633, 1.605, and 1.592 at 405 nm, 488 nm, and 589 nm, respectively (Kasarova et al., 2007). Manufacturers of reference particles typically specify the refractive index at 589 nm, whereas most flow cytometers measure light scattering at 405 nm or 488 nm. As a refractive index of 1.633 may lead to a 39%^7^ increase in the amount of scattered light compared to a refractive index of 1.592, the illumination wavelength should be taken into account when interpreting the refractive index of reference particles or selecting a refractive index for calculations. The refractive index of substances, such as bulk polystyrene and silica, can be readily found in literature for different wavelengths (Polyanskiy). Currently, the refractive index of reference particles is assumed to be equal to the refractive index of the bulk substance. However, measurements with calibrated flow cytometers suggest that the refractive index of reference particles may differ from the refractive index of bulk substances (van der Pol et al., 2012). Therefore, metrologically traceable measurements are needed to determine the refractive index of reference particles and buffer solutions within a known uncertainty (Kuiper et al., 2022).

Refractive index determination of solid reference particles and particularly EVs is technologically difficult (Section 7.2). Unlike solid particles, the refractive index of EVs is distributed like a core‐shell particle (Section 2.1.1), because the refractive index of the phospholipid membrane (1.40—1.52) is higher than the refractive index of the lumen (1.34–1.42) (Brunsting & Mullaney, 1974; Curl et al., 2005; Ducharme et al., 1990; Ghosh et al., 2006; Horvath et al., 2003; Kienle et al., 2014; Maltsev et al., 2011; Valkenburg & Woldringh, 1984; van Manen et al., 2008). Hence, it is incorrect to assign a single refractive index to EVs. Refractive index statements of an EV should rather be interpreted as an effective refractive index, describing the refractive index of a solid particle which scatters the same amount of light as an equivalent sized core‐shell EV (van der Pol et al., 2021). The concept of an effective refractive index is needed because hitherto only the effective refractive index has been measured (Gardiner et al., 2014; Konokhova et al., 2012; Konokhova et al., 2016; Konokhova et al., 2016; van der Pol et al., 2018; van der Pol et al., 2014). The effective refractive index of EVs increases with decreasing diameter of EVs, because the ratio of phospholipid membrane (high refractive index) to lumen (low refractive index) increases. Studies measuring the effective refractive index of EVs have been predominantly conducted at 405 nm and show that most EVs >100 nm in plasma, urine and various conditioned cell culture media have an effective refractive index ⩽ 1.40 (de Rond et al., 2019; Gardiner et al., 2014; Geeurickx et al., 2019; Konokhova et al., 2012; van der Pol et al., 2018; van der Pol et al., 2014).

##### 2.2.3.2 Scattering cross section

**FIGURE 5:**
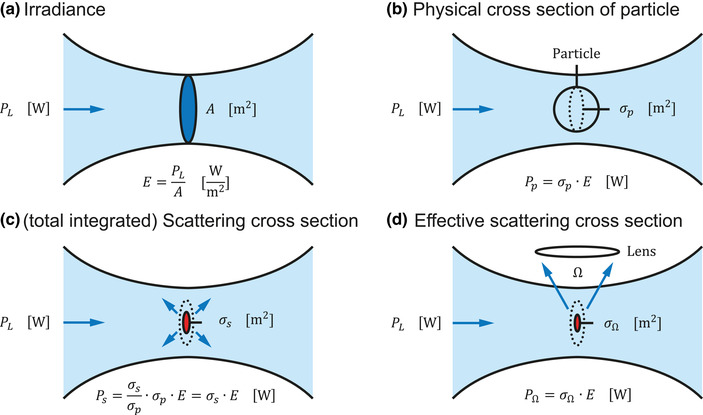
Irradiance of light and the physical cross section and scattering cross section of a particle. (A) Irradiance *E* is the power of light $P_{L}$ per unit area *A* in the focus (blue oval) of the laser beam. (B) Physical cross section $\sigma_{p}$ (dotted oval) of a particle (circle) oriented perpendicular to the incoming laser beam. For clarity, $\sigma_{p}$ and *A* (panel A) have similar dimensions, but in reality $\sigma_{p}$ for extracellular vesicles is much smaller thanA. (C) The scattering cross section $\sigma_{s}$ (red oval) is a hypothetical area defining which fraction of the incoming light is scattered by a particle. (D) The effective scattering cross section $\sigma_{\Omega }$ (red oval) is a hypothetical area defining which fraction of the power of the incoming light is scattered by a particle towards a lens with solid collection angle Ω.

To understand how the effective refractive index of EVs, which is low compared to commonly used reference particles, affects light scattering of EVs, first the concept of scattering cross section needs to be introduced. The scattering cross section determines how efficiently a particle scatters light and can be calculated with light scattering theory (Sections 2.2.3.3 and 2.2.3.4). To understand the concept of scattering cross section, it is important to understand the difference between the irradiance and power of light. In optical physics, the term power refers to the amount of energy that light transfers per second, usually in units of Watts [W]^8^. In turn, the term irradiance refers to the power [W] of light per unit area [m^2^]. To illustrate the meaning of irradiance, Figure 5(A) shows a laser with power $P_{L}$ that is focused to an area *A*. As a result, the irradiance *E* in the focus is given by:^9^

(3) $$E\;=\frac{P_{L}}{A}\;\left[\frac{W}{m^{2}}\right]$$

Figure 5(B) shows a spherical particle that is illuminated by a focused laser beam. For clarity, the particle has a slightly smaller diameter than the focused laser beam. In reality, however, EVs are much smaller than the focused laser beam. The cross‐sectional area of the particle that is oriented perpendicular to the laser beam is defined as the physical cross section of the particle $\sigma_{p}$, such that the power $P_{p}$ impinging on the particle is:

(4) $$P_{p}=\sigma_{p}\;\cdot E\;\left[W\right]$$

From a geometrical perspective, it would be expected that the fraction of laser light that is blocked and scattered by a particle is $\sigma_{p}/A$. However, part of the light may pass through the particle unhinderedly and part of the light missing the particle may change direction due to diffraction. Instead of $\sigma_{p}/A$, the fraction of light that is scattered by a particle is $\sigma_{s}/A$, where $\sigma_{s}$ is a hypothetical area called the scattering cross section. Figure 5C shows $\sigma_{s}$ for an EV. Here, $\sigma_{s}$ is smaller than $\sigma_{p}$, which is typical for low‐refractive index particles such as EVs. From the power of light $P_{p}$ impinging on the particle, only the fraction impinging upon $\sigma_{s}$ is scattered by the particle. Thus, the power of light scattered by a particle $P_{s}$ is given by:

(5) $$P_{s}=\frac{\sigma_{s}}{\sigma_{p}}\;\cdot P_{p}=\frac{\sigma_{s}}{\sigma_{p}}\cdot \sigma_{p}\cdot E=\sigma_{s}\;\left(d,n_{m},n_{p},\lambda \right)\cdot E\;\left[W\right]$$

The variables within the brackets show that $\sigma_{s}$ depends on the particle diameter *d*, the refractive index of the medium $n_{m}$ and the effective refractive index of the particle $n_{p}$, and the illumination wavelength λ. For core‐shell particles, like EVs, $\sigma_{s}$ does not depend on $n_{p}$, but on the refractive index and thickness of the shell and the refractive index of the core. When the aforementioned variables are known, $\sigma_{s}$ can be calculated for spherical and core‐shell particles (Section 2.2.3.4). Equation (5) shows that $P_{s}$ scales linearly with the irradiance of the focused laser beam. Following Equation (3), this means that $P_{s}$ scales linearly with the laser power and inversely with the area of the focused laser beam. Thus, doubling the laser power and halving the area of the focused laser beam will quadruplicate the light scattering signal. Therefore, flow cytometers dedicated to nanoparticle detection are typically equipped with lasers that have higher power and/or are stronger focused than lasers used in traditional flow cytometers (de Rond et al., 2020; Jung et al., 2018; Zhu et al., 2014).

Figure 5C and Equation (5) describe the power of light scattered by a particle into all directions. To emphasize that light is scattered into all directions, $\sigma_{s}$ should rather be called the total scattering cross section. Flow cytometers, however, do not detect light scattered into all directions, but detect only the scattered light collected by a lens. In Figure 5(D), the scattering cross section $\sigma_{s}$ has been replaced with the effective scattering cross section $\sigma_{\Omega }$, which is a hypothetical area defining which fraction of the incoming light is scattered by a particle towards a lens with solid collection angle Ω. In geometry, the solid angle is a measure of the range of connected angles. As the scattered light collected by the lens is a fraction of the totally scattered light, $\sigma_{\Omega }$ is smaller than $\sigma_{s}$. Please note that in EV FCM literature (de Rond et al., 2018; van der Pol et al., 2012; Welsh et al., 2020), the term effective scattering cross section is interchangeably used with the term scattering cross section. Derived from Equation (5), the power of light scattered by a particle towards a lens with solid collection angle Ω, $P_{\Omega }$, is given by:

(6) $$P_{\Omega }=\sigma_{\Omega }\;\left(d,n_{m},n_{p},\lambda ,\Omega \right)\cdot E\;\left[W\right]$$

In practice, however, *E* is unknown, the optical losses are unknown, and $P_{\Omega }$ is measured in arbitrary units (arb. unit) instead of W. The strength of the concept of scattering cross section is that, despite all the unknowns, the measured $P_{\Omega }$ in arbitrary units can still be related to the theoretically calculated $\sigma_{\Omega }$ by solving the linear scaling factor *F* in the following equation (de Rond et al., 2018):

(7) $$P_{\Omega }=\;F\cdot \sigma_{\Omega }\left(d,n_{m},n_{p},\lambda ,\Omega \right)\;\left[\mathrm{arb}.\;\mathrm{unit}\right]$$

Equation (7) provides the essential step to calibrate a light scattering detector of a flow cytometer, which is further discussed in Section 6.3.2. The next two sections discuss two light scattering theories for calculating $\sigma_{\Omega }$.

##### 2.2.3.3 Rayleigh scattering

**FIGURE 6:**
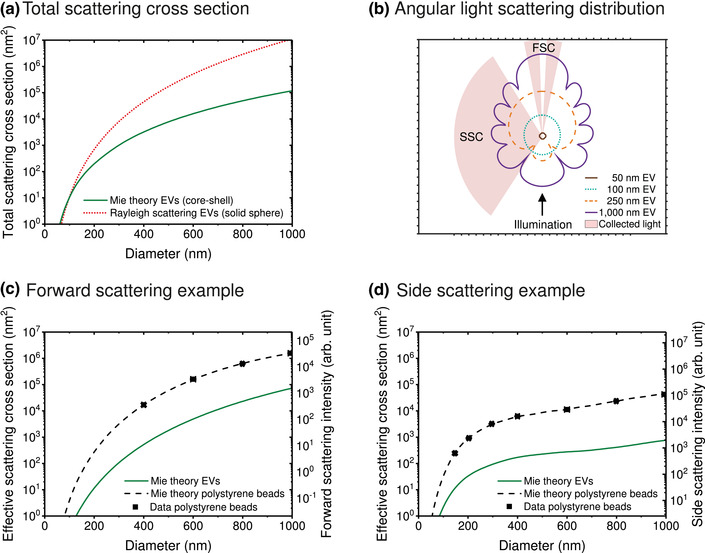
Application of Mie theory calculations for flow cytometry measurements of extracellular vesicles (EVs). (A) Total scattering cross section versus diameter for EVs calculated with Mie theory (solid line) and the Rayleigh approximation for solid spheres (dotted line). (B) Mie calculation of the angular distribution of light intensity scattered by an EV with a diameter of 50 nm (circle), 100 nm (dotted line), 250 nm (dashed line) and 1000 nm (solid line). The numerical apertures of the forward scattered light (FSC) and side scattered light (SSC) detectors are 0.29 and 1.19, respectively. (C) Effective scattering cross section and scattering intensity in arbitrary units (arb. unit) versus diameter for EVs (solid line) and polystyrene beads (dashed line) calculated with Mie theory and measured (symbols) with a customized BD FACSCanto (de Rond et al., 2020). The collection angles are similar to the FSC detector in panel B. (D) Idem as panel C, but the collection angles are similar to the SSC detector in panel B. EVs were modelled as core‐shell particles having a 6 nm thick shell with a refractive index of 1.48 and a core refractive index of 1.38. Polystyrene beads were modelled as solid spheres with a refractive index of 1.6053. The model assumed an illumination wavelength of 488 nm, a medium refractive index of 1.3374. and a polarized laser beam with the electric field component being perpendicular to the plane displayed in panel B.

Rayleigh scattering is scattering of light by particles that are smaller than ∼1/10^th^ the illumination wavelength^10^. However, the diameter of most, if not all, EVs exceeds 1/10^th^ the illumination wavelengths used in flow cytometers. Moreover, the Rayleigh approximation for solid spheres does not take into account that the shell of EVs has a higher refractive index than the intra‐vesicular lumen. Therefore, instead of the Rayleigh approximation, Mie theory is the theory of choice to calculate scattering cross sections of EVs. For comparison, Figure 6(A) shows the total scattering cross section versus diameter for EVs calculated with the Rayleigh approximation for solid spheres and Mie theory. For EVs larger than 100 nm in diameter, the Rayleigh approximation overestimates the real scattering cross section of EVs.

##### 2.2.3.4 Mie theory

Throughout this compendium, the term Mie theory refers to the Mie solution of Maxwell's equations, which describes the scattering of an electromagnetic plane wave by a homogeneous sphere, a core‐shell particle, or an infinite cylinder. Maxwell's equations accurately describe how electric and magnetic fields are generated by and interact with charges. As the Mie solution provides an analytical solution of Maxwell's equations, Mie theory does take into account interference and is applicable to spherical and core‐shell particles of all sizes relative to the illumination wavelength. The Mie solution does not result in a single equation, but takes the form of mathematical infinite series (Bohren & Huffman, 1983), which is beyond the scope of this compendium. However, commercial solutions, free software, and programming scripts are widely available to use Mie theory to describe light scattering signals (de Rond et al., 2018).

In EV FCM, Mie theory is used to calculate the angular distribution of light intensity scattered by reference particles and EVs (de Rond et al., 2018; Konokhova et al., 2012; Konokhova et al., 2016; van der Pol et al., 2012; Welsh et al., 2020). Figure 6(B) shows a Mie calculation of the angular scattering distribution, also called the phase function, of an EV with a diameter of 50 nm, 100 nm, 250 nm and 1000 nm. The illumination light propagates from the bottom to the top and has an electric field component that is oriented perpendicularly to the imaged plane. For the imaged plane, a 50 nm and 100 nm EV scatter light isotropically, meaning that the same intensity of light is scattered into all directions. Due to optical interference, EVs of 250 nm and 1000 nm in diameter have a more complex angular scattering distribution with increased forward scattering compared to smaller EVs. Although for clarity the radial scale of Figure 6(B) is omitted, the impact of the actual logarithmic scale is emphasized by the 10^9^‐fold difference in forward scattered light between 50 and 1000 nm EVs.

Integration of the angular scattering distribution over all collection angles results in the total scattering cross sections, which are 0.38 nm^2^, 10.6 nm^2^, 476 nm^2^, and 0.12 μm^2^ for EVs with a diameter of 50 nm, 100 nm, 250 nm and 1000 nm, respectively. Figure 6(A) shows the total scattering cross section versus diameter for EVs calculated with Mie theory. Within the size range of EVs, the total scattering cross section increases multiple orders of magnitude with increasing diameter. Integration of the angular scattering distribution over the solid angles in which the forward and side scattered light are detected, as depicted in Figure 6(B), results in the effective forward and side scattering cross sections of EVs shown in Figure 6(C) and (D), respectively. The collection angles represent the actual configuration of a customized BD FACSCanto (de Rond et al., 2020). The effective scattering cross sections for forward scattered light (FSC) increase continuously with diameter (Figure 6C), whereas the effective scattering cross sections for side scattered light shows oscillations (Figure 6D), which are attributed to the side scatter lobes of the angular scattering distribution. From Figure 6(C) and (D) it is clear that the collection angles strongly affect the relation between light scattering and particle diameter. To make the relation between the panels of Figure 6 more insightful, Supplemental Materials 1 contains a video of the complex relationship between the angular scattering distribution of EVs, polystyrene beads, and silica beads and the measured FSC and side scattered light (SSC) intensities.

For comparison, Figure 6(C) and (D) show the theoretical effective scattering cross section and the measured scattering intensity of polystyrene beads as well. As polystyrene beads have a refractive index of 1.605, whereas EVs have a core‐shell structure with a lower effective refractive index^11^, polystyrene beads scatter one to two orders of magnitude more light than similar‐sized EVs. The discrepancy between the optical properties of EVs and polystyrene beads together with the strong dependency of the scatter to diameter relation on collection angles show that polystyrene beads should not be used to interpret light scattering signals of EVs without the use of Mie theory. Mie theory describes the data in Figure 6(C) and (D) with coefficient of determination, *R^2^*, exceeding 0.996, which demonstrates how accurately Mie theory describes light scattering of solid spheres.

In summary, Mie theory is used to calculate the angular scattering distribution of solid spheres, such as polystyrene beads, and core‐shell particles, such as EVs. The effective scattering cross section of a particle for a specific light scattering detector of a flow cytometer can be calculated from the angular scattering distribution. Knowledge on the relation between the particle diameter and refractive index and light scattering measured by a flow cytometer allows for size determination of EVs (Section 7.1), standardization (Section 6.3.2) and label‐free particle identification (Section 7.2).

### 2.3 Flow cytometry

A flow cytometer is an instrument used to measure fluorescence and light scattering signals from single particles in liquid suspension that are flowing through a focused light source, most commonly a laser beam. This chapter describes the basic components of a flow cytometer, which are the fluidics (Section 2.3.1), optics (Section 2.3.2) and electronics (Section 2.3.3). In addition, Section 2.4 describes the differences between traditional flow cytometers and sorting, imaging and spectral flow cytometers, which have also been used to characterize EVs. Each section presents a description of the basic components and discusses examples and limitations specific to EV FCM.

#### 2.3.1 Fluidics

**FIGURE 7:**
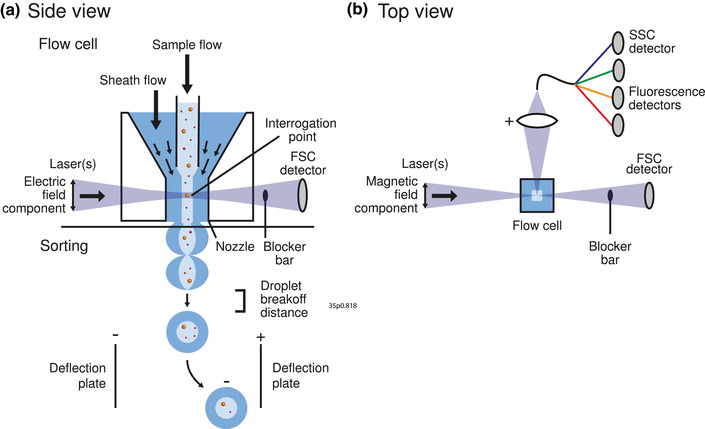
Schematic representation of a flow cytometer. (A) Side view of the flow cell. Particles in a sample flow are hydrodynamically focused by a sheath flow and guided through the center of one or more focused lasers. The point where the sample flow and laser beam intersect is called the interrogation point. The electric field component of the laser beam is aligned parallel to the flow direction. Part of the light scattered by illuminated particles in the sample flow is measured by the forward scattered light (FSC) detector. The laser beam is blocked by the blocker bar. Flow cytometers with the functionality to sort particles are typically equipped with a nozzle that, together with a piezo element, generates droplets. A droplet contains both sample and sheath fluid, and may contain one or more particles. To separate the droplets, an electric charge is applied to the fluid. Depending on the applied charge, a static electric field will deflect the charged droplets towards different containers. (B) Top view of the flow cell. Illuminated particles emit fluorescence and scatter light in the sidewards direction, which is collected by a positive lens, spectrally filtered, and measured by fluorescence detectors and the side scattered light (SSC) detector, respectively. The magnetic field component of the laser beam is aligned perpendicular to the flow direction.

The fluidics system of a flow cytometer has three basic functions (Austin Suthanthiraraj & Graves, 2013). First, the fluidics system is responsible for sample delivery at the laser intercept, which is called the interrogation point. Second, at the interrogation point, samples require accurate positioning. Ideally, particles in the sample pass through the laser intercept at the focus in a high and constant throughput. The third function is optional and involves sorting of particles after detection (Section 2.3.1.1). Although different configurations of the fluidics exist, most commonly a sample flow is surrounded by a second flow, called the sheath flow, as shown in Figure 7(A). The width of the sheath flow is limited by the internal dimensions of the flow cell, which are typically 100–200 μm by 200–400 μm (Shapiro, 2003)^12^.

In flow cytometers with a sheath flow, positioning of the sample is typically done by hydrodynamic focusing. To ensure that particles flow through the focus of the laser intercept, the sample flow is dragged or forced into the center of the sheath flow, which usually consists of PBS or water. Figure 7A shows that along the way to the interrogation point, both the sheath flow and the sample flow become narrower, thereby centering the sample flow in the flow cell. Hydrodynamic focusing works because the sheath and sample fluids form a laminar flow, meaning that the fluids follow smooth paths in layers with little or no mixing and a constant flow velocity. The sheath fluid centers the sample flow and prevents contact between the sample and the flow cell.

To deliver the sample to the sheath flow, two methods are commonly used. The first method is based on a differential pressure between the sample flow and sheath flow. When the pressure of the sample flow exceeds the pressure of the sheath flow, the sample is driven into the sheath flow. Once calibrated (Section 6.2), differential pressure systems allow smooth and consistent delivery of the sample. A disadvantage are variations in pressure, which results in an unstable sample flow rate, thereby making volumetric measurements and particle concentration measurements inaccurate. The second method pumps the sample into the sheath flow by either a syringe or a peristaltic pump. With syringe pumps, the volume of the measured sample can be regulated more accurately than with differential pressure systems. A disadvantage is that pumps can introduce pulsations of the flow rate, thereby causing variations in the sample delivery rate and thus affecting the signal stability.

The pressures of the sheath and sample flow determine their flow rates and thus determine the width of the sample flow, which is typically about 20 μm (Shapiro, 2003)^14^. Increasing the sample pressure leads to a higher sample flow rate, an increased width of the sample flow, and an increased interrogation volume. Particularly for EV detection, increasing the interrogation volume may cause artefacts, such as swarm detection (Section 5.7). Hence, EVs are commonly detected at low flow rates when compared to cells. Flow rates are typically between 1 uL·min^−1^ for flow cytometers equipped with a syringe pump and up to 120 uL·min^−1^ for flow cytometers with pressure driven flow. The particle velocity through the interrogation point is typically 1–10 m·s^−1^ (Shapiro, 2003).

An addition to hydrodynamic focusing is acoustic focusing, wherein hydrodynamic forces and sound waves are combined to align the particles within the sample flow. For cells, acoustic focusing allows higher sample flow rates, as acoustic radiation guide the cells through the centre of the laser intercept, regardless of the width of the sample flow. For EVs, however, acoustic radiation forces are negligible, making acoustic focusing of limited use to EV FCM.

Although the sheath fluid type is often determined by the flow cytometer, ideally the sheath fluid and sample dilution buffer have the same composition to prevent scattering caused by refractive index differences at the interface of the sheath fluid and sample buffer. To prevent damage of EVs by osmosis, EVs need to be diluted in an isotonic buffer solution, such as saline or PBS. Although PBS seems an obvious choice for a sheath fluid, PBS may increase the formation of calcium phosphate crystals in the fluidics system, which may cause additional background noise, clogging, or loss of laminar flow (Cossarizza et al., 2019; Shapiro, 2003). Therefore, several new flow cytometers dedicated to detection of submicrometer particles use purified water as a sheath fluid (Barranco et al., 2019; Pospichalova et al., 2015).

To minimize background counts originating from particles present in the sheath fluid, most flow cytometers contain inline filters with an effective pore diameter of 0.22 μm, 0.1 μm or 0.04 μm to remove such particles. Consulting the instrument manufacturer before replacing filters is recommended, because changing filters may affect the velocity of the sheath flow and hence the laser time delay settings (Section 2.3.3.3).

##### 2.3.1.1 Sorter fluidics

Some flow cytometers are designed to sort particles (Section 2.4.1). To compartmentalize particles in the sample, flow cytometry sorters generate droplets. There are two designs for droplet‐based cell sorters: flow cell based and jet‐in‐air. The main difference between these designs is the position of the interrogation point. Figure 7(A) shows a flow cytometer based on a flow cell, where the interrogation point occurs in a closed flow cell before the nozzle. In a jet‐in‐air sorter, the interrogation point is directly in the fluid stream, downstream from the nozzle. Due to the high refractive index contrast between air and water, which causes stray light, signal loss, and precludes the use of a gel‐immersed collection lens, jet‐in‐air systems typically have a lower light collection efficiency than systems based on a flow cell (Fox & Coulter, 1980).

A piezo in the flow cell induces a high frequency oscillation in the fluid stream, which causes a break‐off of the fluid stream into droplets at a constant position beneath the interrogation point. Note that a droplet contains both sample and sheath fluid, and may contain no particle, a single particle, or multiple particles. The time between particle detection and particle arrival at the break‐off point is precisely known, because both the break‐off point and the flow velocity are constant. Once a particle of interest reaches the break‐off point, an electric charge is applied to the fluid to charge the droplet surrounding the particle. Next, a static electric field will deflect charged droplets and will separate the particles in different containers depending on the applied charge. Uncharged droplets follow a straight path and are directed to a waste container. By applying different charges to different particles of interest, it is possible to separate multiple populations of particles.

#### 2.3.2 Optics

The optics of a flow cytometer can be divided into the illumination and detection optics. The illumination optics focuses light onto the particle, whereas the detection optics collect and propagate optical signals generated by the particle towards the fluorescence and light scatter detectors. In flow cytometers with a flow cell (Figure 7), the illumination optics, detection optics, and sample flow come together in the flow cell, which is a core component of the flow cytometer.

##### 2.3.2.1 Flow cell

Flow cells are most commonly a hollow quartz cuboid. The inner dimensions of the flow cell are typically 100–200 by 200–400 μm (Shapiro, 2003), but differ between flow cytometers. Together with the detection optics, the flow cell determines the solid angle of the collected light. The larger the solid angle, the more light can be collected and propagated towards the detectors, and the higher the signal levels are. As illustrated in Figure 7(B), most flow cytometers have a rectangular flow cell with the longest edge placed in parallel to the incoming laser beam, which allows a larger solid collection angle for fluorescence and SSC than for FSC.

##### 2.3.2.2 Illumination optics

To excite fluorophores with different absorption spectra, flow cytometers are typically equipped with lasers of different wavelengths that illuminate the particles at different positions along the sample flow. A common illumination source are continuous wave diode lasers, which deliver stable, high power, single wavelength, and polarized light. Diode lasers of virtually all visible wavelengths are available. Typical illumination wavelengths are 488 nm (blue) and 633 nm (red), which are the emission wavelengths of the formerly widely available ion argon laser and helium neon laser, respectively. New flow cytometers commonly have 405 nm (violet) illumination, which allows excitation of new fluorophores and provides a ∼2‐fold enhanced light scattering efficiency compared to 488 nm illumination (Section 2.2.3.4). In practice, the use of lasers with a shorter wavelength does not guarantee an increased ability to detect small particles, because (1) below a wavelength of approximately 400 nm standard optical components become opaque and silicon detectors lose sensitivity, and (2) the lower LoD is determined not only by the power of light scattered by a particle, that is, the signal, but also by the background noise (Section 2.3.3). Therefore, the choice of illumination wavelengths rather depends on the absorption spectrum of the used fluorophores.

Typical laser powers in flow cytometers are between 10 mW and several hundreds of mW (Shapiro, 2003). A higher illumination power generally results in improved sensitivity for a given optical configuration, although there are exceptions due to for example photobleaching (Section 2.2.2.3) and a weakly focused beam. A weakly focused beam with relatively high power may produce a lower irradiance than a strongly focused beam with low power. Hence, the optical components responsible for shaping and focusing the laser beam are essential to the sensitivity and performance of the flow cytometer. To achieve homogeneous and constant illumination while allowing micrometer variations of the sample flow position, the laser beam is traditionally focused to a cylindrically shaped beam spot with a Gaussian irradiance profile. Dimensions of the focused laser beam were typically 60–150 μm in the direction perpendicular to the flow and 5–20 μm in the direction parallel to the flow (Cossarizza et al., 2019; Shapiro, 2003). A cylindrically shaped beam spot has two disadvantages. First, only a fraction of the laser power is used to illuminate the particle. Second, the interrogation volume is large compared to EV dimensions, thereby increasing the risk of swarm detection (Section 2.3.3.4). To increase the irradiance and decrease the interrogation volume, while still allowing micrometre variations of the sample flow position, innovations like a flat‐top beam profile are used in new flow cytometers, such as the Cytek Aurora. In contrast to conventional cylindrically shaped beam spots, a flat‐top beam profile ensures that the entire laser power is used to illuminate the interrogation volume while remaining a homogeneous illumination profile (Coumans et al., 2012).

Regarding the polarization direction of the illumination (Section 2.2.1), the electric‐field component of the polarized laser beam is aligned in parallel with the sample flow, as shown in Figure 7(A). This orientation of the polarization ensures that SSC is collected perpendicular to the electric‐field component, which maximizes the SSC signal.

##### 2.3.2.3 Detection optics

Detection optics collect, spectrally separate, and propagate light towards the detectors, converting both fluorescent and scattered light into electronic signals. The numerical aperture (NA) of the collection lens describes the solid angle over which the lens collects light. Most flow cytometers have a high‐NA (>1.0) lens to collect fluorescence and SSC, and a low‐NA (<0.5) lens to collect FSC. To minimize the loss of optical signals, the high‐NA lens is typically a multi‐element lens that is gel‐coupled to the flow cell. SSC is generally more suitable for EV detection than FSC, because (1) the laser beam does not propagate towards the SSC lens, which means that the SSC lens collects less background light than the FSC lens, (2) the SSC lens typically has a higher NA and therefore a higher light collection efficiency than the FSC lens, and (3) EVs with a diameter smaller than about 300 nm scatter more light in the SSC direction relative to the FSC direction (Section 2.2.3.4).

After collection, light is commonly coupled into optical fibres to add confocality and to propagate the light towards the optical components responsible for spectral separation. Adding confocality means to attenuate background scattering originating from the flow cell or from particles in the sheath fluid in a similar way as in a confocal microscope. The goals of spectral separation are (1) to separate scattered light, which has the same wavelength as the illumination, from fluorescence emission, which has a longer wavelength than the illumination, and (2) to separate the fluorescence emission from fluorophores emitting at different wavelengths. Spectral separation is commonly achieved using sequentially placed spectral filters, but it can also be done by wavelength division multiplexing or other types of spectrometers (Section 2.4.3). To maximize sensitivity, spectral filters should match the emission spectra of the used fluorophores. However, when fluorophores with overlapping emission spectra are used, spectral overlap should be taken into consideration when designing spectral filters.

After spectral separation, detectors convert light to electronic signals. Two key parameters describing the performance of optical detectors are the quantum efficiency, which describes the ratio of photons that are converted into measurable electrons to the total number of incoming photons, and the signal to noise ratio (S/N). The three most common detector types in non‐imaging flow cytometers are photodiodes, photomultiplier tubes (PMTs), and avalanche photodiodes (APDs). Photodiodes are typically used to detect light scattering from cells. Although the quantum efficiency of a photodiode is high (∼40%–90%) compared to PMTs (∼10%–30%), photodiodes have no internal amplification of the signal. Therefore, photodiodes have a low S/N compared to PMTs and are unsuitable for EV detection. Today many modern flow cytometers continue to use photodiodes for FSC, but have switched to PMTs or APDs for SSC.

Classically, PMTs were the detector of choice for low light applications in FCM. With a ∼10%–30% quantum efficiency, low noise, and fast response time, PMTs outperform other detectors in the visible region of the spectrum. However, the photon‐level sensitivity of PMTs comes at the expense of price, fragility, size, sensitivity to magnetic fields, and high‐voltage power‐supply requirements.

Silicon APDs have a quantum efficiency of 30%–90% and are more robust than PMTs, but at the expense of higher noise and decreased response time. In the red and near‐infrared (IR) part of the spectrum, current APDs outperform PMTs in terms of sensitivity. At 405 nm illumination, flow cytometers equipped with APDs (e.g., BC CytoFLEX) or PMTs (e.g., Apogee A60‐Micro) are both able to detect single 80 nm polystyrene beads by SSC. It is important to note that there are many types of APDs and PMTs, designed for various different applications. Additionally, detectors of the same manufacturer and type can also differ in performance and therefore require individual characterization during instrument set up.

#### 2.3.3 Electronics

After the detectors have converted light into electronic signals, the signals are processed by the electronics. A basic understanding of the electronics is important both for setting up the flow cytometer and for data interpretation. The functions of the electronics are to (1) convert the analogue electronic signals to digital signals, (2) differentiate optical signals associated with a particle from the background noise, (3) derive statistics to summarize the optical signals from each particle, and (4) relate signals from multiple detectors to a single particle. This section elucidates the basic signal processing steps of the electronics of a flow cytometer. However, the exact implementation of the electronics varies with each manufacturer. Once the electronic signal processing is performed, summarized data is saved on a computer for further analysis.

##### 2.3.3.1 Analogue to digital converter

Over time, the electronics have moved from analogue to semi‐digital and then to fully digital (Snow, 2004; Xiong & Tanik, 2015). The following explanation will focus on flow cytometers with fully digital electronics, wherein the analogue electronic signals leaving the detector are pre‐amplified and directly converted to digital signals by the analogue to digital converter (ADC), such that all further signal processing steps can be done digitally. In a fully digital system, the detected signal and the optical signals closely follow a linear relationship within the full dynamic range of detection. In other words, the detected signal accurately describes the optical signal, which is especially important to calibrating the flow cytometer (Section 6.3). Older flow cytometers with analogue or semi‐digital electronics do not follow this linear relationship and so are more challenging for instrument calibration.

**FIGURE 8:**
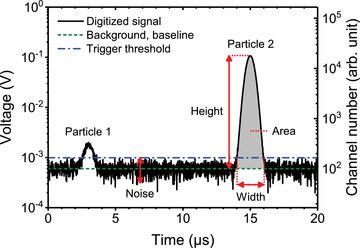
**Basic signal processing steps of the electronics of a flow cytometer**. Simulated electrical signal originating from a detector (left axis) and the same signal digitized by the analog to digital converter (right axis) versus time. The signal has an offset due to amplifiers and gains and fluctuates due to background noise. The median background level is called the baseline (dashed line). At 3 μs and 15 μs, particle 1 and particle 2 pass the interrogation point, respectively, and produce a Gaussian pulse. To identify the signal associated to a particle, a trigger threshold is set at three times the standard deviation of the background noise (dash dotted line). The pulse width is the time interval wherein the pulse exceeds the trigger threshold level (dotted lines). The pulse area is the area under the pulse (gray), but above the baseline. The pulse height is the amplitude of the pulse relative to the baseline. Arb. unit: arbitrary units.

Figure 8 shows a simulation of the electrical signal, such as an electric current or voltage, originating from a detector versus time. To digitize the signal, the ADC scales the analogue electrical signal onto an axis with a fixed number of equally sized bins called channel numbers. These channel numbers are closely related to the arbitrary unit scale stored in FCS data files. As the first decade of the ADC only has 10 channel numbers available to describe the signal (Section 2.3.3.5), the baseline of the background noise is typically scaled to a channel number substantially higher than 1, such as channel number 100 (Figure 8).

##### 2.3.3.2 Differentiating optical signals from background noise

After the signal is digitized, the electronics differentiate optical signals attributed to particles from background noise. Even when no particle is present in the interrogation point, the ADC continuously receives signals associated with electronic, fluidic, and optical noise. Electronic noise can originate from dark current of the detector and thermal noise. Fluidic noise originates from particles in the sheath fluid. Optical noise can originate from black body radiation, fluorescence and Raman scattering of the buffer and/or sheath fluid, and stray light. The optical noise is typically higher than the electronic noise. For scatter detectors, optical noise is dominated by stray light resulting from laser illumination. The difference between optical and electronic noise is usually observed by comparing the difference in signal with lasers on vs. lasers off, respectively (Giesecke et al., 2017; Saleh & Teich, 1991). Electronic and optical noise further involve shot noise, which originates from the discrete nature of light (photons) and electric current (electrons).

Although most of the time no particle is detectable, Figure 8 shows that the signal has an offset and fluctuates due to background noise. At 3 and 15 μs, a particle passes the interrogation point, which typically produces a Gaussian pulse on top of the background noise. The pulse shape is typically Gaussian because laser beams have a Gaussian irradiance distribution. For an optically well‐aligned flow cytometer, the pulse reaches its maximum when the particle is in the centre of the focused beam.

To identify pulses, the electronics apply a digital filter, baseline restoration and a trigger threshold. A digital filter, such as a moving average filter or finite impulse response filter, is applied to attenuate the noise (Xiong & Tanik, 2015). Baseline restoration identifies and subtracts the offset of the background noise^13^, which for example can be achieved by applying a short‐pass filter (Snow, 2004) or a moving median filter. By subtracting the baseline, the base of each pulse will be centred around 0 arbitrary units: the signal level in the absence of a particle. To maintain digital resolution after baseline subtraction (Section 2.3.3.5), flow cytometers use different proprietary procedures.

Next, the trigger threshold acts as a filter that only records events whose signals exceed a defined threshold(s). This threshold is typically set on one or multiple detectors (Section 6.1). In Figure 8, the signal only exceeds the trigger threshold of this detector (dashed line) between 2.5–3.5 μs and 14–16 μs, which corresponds to the time intervals during which the first and second particle pass the interrogation point. The time interval during which a signal exceeds the trigger threshold is registered as one event by the electronics.

##### 2.3.3.3 Deriving pulse statistics from optical signals

The pulse generated during one event is characterized with pulse statistics, including the area, height, and width. The pulse area is the area under the curve of the pulse after baseline restoration, typically multiplied by a scalar to make area equal to height (for a perfectly Gaussian shaped pulse). The pulse height, also named peak, is the amplitude of the pulse, typically after baseline restoration. The pulse width is the time interval that the signal exceeds the trigger threshold. The pulse statistics depend on the particle properties, such as fluorescence brightness, size, and refractive index, but also on the optical configuration and the electronics of the flow cytometer. Once the time interval of an event is known, the electronics calculate the pulse statistics on all detectors. This takes into account the time delay between signals associated with the illumination of a particle by different lasers along the flow direction. The pulse statistics are provided with a time stamp and transferred to a computer and stored in an FCM datafile.

While the concept of pulse area, height, and width are simple, the implementation of the pulse statistics differs between flow cytometers and is often proprietary information. For example, the pulse width typically refers to the time that a pulse exceeds the trigger threshold. Instead of measuring the pulse width, however, many flow cytometers determine the pulse width mathematically from the ratio between the pulse area and height, thereby assuming a Gaussian pulse shape. The use of this mathematical approach, however, leads to limitations in the interpretation of coincidence and swarm detection (Section 2.3.3.4), and background noise (Section 2.3.3.2) (de Rond et al., 2020), which are processes that do not result in a Gaussian pulse shape.

The accuracy of the pulse statistics depends upon the number of datapoints that describe the pulse. The faster the sampling rate, the more datapoints per pulse, and the higher the accuracy of the pulse characteristics. Most ADCs have a sampling rate between 10 and 20 MHz, which means that the signal level is measured between 10 and 20 million times per second. For a typical flow cytometer with a laser height of 10 μm and a core stream velocity of 10 m∙sec^−1^, a sampling rate of 10 and 20 MHz leads to 10 and 20 datapoints per pulse, respectively, to derive the pulse characteristics from. The number of datapoints can be increased by increasing the sampling rate and/or decreasing the flow velocity.

##### 2.3.3.4 Coincidence and swarm detection

Particles in a flow cytometer enter the interrogation point at random moments conforming Poisson statistics (Student, 1907; Poisson, 1837). Therefore, it may occur stochastically that two or more particles are simultaneously illuminated and detected. This is called coincidence. Coincidence may occur at fluorescence or light scatter detectors. The higher the particle concentration, the higher the probability of coincidence events. Most flow cytometers have a method of monitoring coincident events and attempt to remove them by ‘aborting’ the event. An aborted event is named electronic abort and is registered. The ratio of electronic aborts to number of events could be used as a quality control parameter.

A special case of coincidence detection is swarm detection, where instead of two or a few particles, multiple (hundreds or more) particles at or below the LoD are simultaneously and continuously present in the interrogation point. Although each individual particle would be too dim to exceed the trigger threshold, the combined signal of all illuminated particles together exceeds the trigger threshold. Therefore, multiple particles are simultaneously but erroneously measured as a single event, which results in erroneous signals and undercounting the particle concentration.

To understand the analysis of EVs, swarm detection is a particularly important concept, because EVs are at or below the LoD of most flow cytometers. In addition to EVs and other submicron‐sized particles, fluorescent labelling reagents can trigger events due to swarm detection. On the side of instrument design, new instruments could minimize the risk of swarm detection by decreasing the interrogation volume. When preparing samples, swarm detection can be prevented by diluting the sample prior to analysis, and/or by lowering and thus narrowing the sample flow rate, such that only single EVs exceeding the LoD are contributing to the measured event signals. To ensure single EV detection, include serial dilution controls (Section 5.7).

##### 2.3.3.5 Dynamic range, resolution and scaling

The dynamic range is the ratio between the upper and lower LoD and therefore defines the range between the dimmest and brightest light scatter and fluorescence signals that can be measured. It is important to maximize the dynamic range, because EVs are polydisperse and optical signals correlate with the size of EVs. To select the optimal dynamic range of a detector, and to interpret data of dim EVs, it is important to understand how the dynamic range and the scaling of arbitrary units affect the recorded FCM data.

There are two dynamic ranges to consider for flow cytometers: the dynamic range of the ADC (Section 2.3.3.1) and the dynamic range of the detectors. Typically, manufacturers only advertise the dynamic range of the ADC as the “bit depth”. The bit depth *b* determines the number of available channel numbers *N* according to:

(8) $$N\;=2^{b}\;\left[\mathrm{arb}.\;\mathrm{unit}\right]$$

The higher the bit depth, the more available channel numbers to convert analogue signals into, thus the higher the dynamic range of the ADC. Signals exceeding the highest available channel number are truncated and typically represented by the highest available channel number. As technology has progressed, the bit depth of ADCs in flow cytometers has increased. For example, the BD FACSCalibur had a 10‐bits ADC (1024 channels), whereas the BD FACSCanto had an 18‐bit ADC (262,144 channels). Most current flow cytometers have ADCs ranging from 18 to 24‐bits, an exception being the BC Astrios EQ with a 32‐bit ADC (4,294,967,296 channels).

**FIGURE 9:**
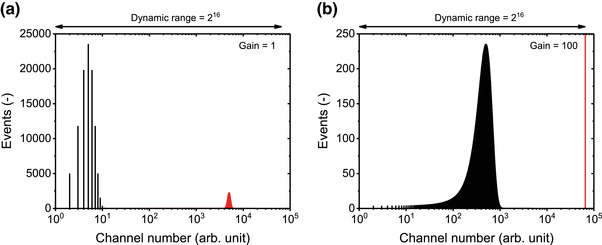
Scaling and the dynamic range of an analog to digital converter (ADC) affect the resolution of a digitized signal. (A) Simulated signal distribution of two particle populations after digitization by a 16‐bit ADC using a gain of 1. The first and second population fall within the first and fourth decade of the ADC, respectively. The first decade has only 10 channel numbers available to describe the signal, which results in poor resolution. (B) Simulated signal distribution of the same particle populations as in panel A after digitization by a 16‐bit ADC using a gain of 100. The first population falls within the third decade of the ADC and has improved resolution compared to panel A. The second population falls off scale, equals the maximum channel number, and therefore cannot be resolved from populations with similar intensities. Arb. unit: arbitrary units.

Besides the dynamic range, the bit depth of the ADC affects the resolution, which dictates the ability of a flow cytometer to distinguish two different signal levels. For example, Figure 9(A) shows a simulation of the signal distribution of two particle populations after digitization by a 16‐bit ADC. The first and second population fall within the first and fourth decade of the ADC, respectively. However, the first decade has only 10 channel numbers available to describe the signal. As a result, the histogram contains 10 spikes and hence the resolution is poor.

To minimize losses in resolution caused by digitization, electric signals are usually scaled to higher channel numbers than 1. For example, by applying an electric gain of 100, the signal of the first particle population would fall within the third decade of the ADC, as shown in Figure 9(B). Due to the electric gain, the particle population is described with 1,000 channel numbers, which is a 100‐fold increase in resolution compared to Figure 9(A). However, due to the limited bit depth of the ADC, the second population falls off‐scale and therefore equals the maximum channel number, namely, 2^16^ = 65,536. Thus, the bit depth of the ADC defines the maximum gain that is allowed before the brightest signal exceeds the maximum channel number. Consequently, a higher bit depth is not only associated with a large dynamic range, but also with the ability to resolve dim signals.

For bright signals, the resolution is limited by other factors aside from the bit depth of the ADC, such as the detector and the stability of the sample flow and illumination. Moreover, the detector not only impacts the resolution, but also affects the dynamic range, because the detector itself has a lower and upper LoD, which are affected by the settings of the detector. For example, when the detector voltage or gain is increased, the dynamic range of the detector decreases. In turn, the dynamic range and the resolution of a detector can be higher or lower than those of the ADC. Therefore, the specified bit depth of the ADC does not necessarily describe the dynamic range of the flow cytometer. Determination of the dynamic range of the full system requires reference signals in comparable, standard units, which could be obtained by a calibration using reference particles (Section 6.3) or a light emitting diode pulser.

### 2.4 Alternative flow cytometer implementations

This section covers background information on the distinction between conventional flow cytometers and alternative flow cytometry technologies that offer new capabilities, such as sorting of particles, imaging of cells and spectral measurements.

#### 2.4.1 Flow cytometry sorters

FCM sorters have been successfully applied to submicron‐sized particles including EVs (Kormelink et al., 2016; Morales‐Kastresana et al., 2019). Sorting offers the opportunity to study the content or function of EVs with a specific antigen expression. When sorting EVs, additional variables need to be considered such as: the sorting time, dilution, and purity of the sorted EVs.

Isolation of EVs with current commercially available sorters is impractical for downstream applications (Morales‐Kastresana et al., 2019; Morales‐Kastresana et al., 2020). Downstream applications require a higher number of EVs than can be sorted within a practical time. For example, bulk assays typically report the number of measured EVs in μg of EVs per ml fluid, implying that at least one billion EVs are required to be detected (Sverdlov, 2012). Although FCM sorters can characterize and sort tens of thousands of particles per second, not all particles are EVs of interest and hence sorting a billion of relevant EVs typically takes more than a day. Besides being impractical, sorting times of a day may cause issues with sterility.

Sorting results in the dilution of the sorted sample. In addition to the dilution that may be introduced by fluorescent staining (Section 4.1) or to prevent swarm detection (Section 5.7), particles are further diluted by sorting because sorting is based on separating particles within droplets of both sample and sheath fluid (Section 2.3.1.1). Depending on the settings, the volume of one droplet is in the nL range. Therefore, attempting to sort a billion EVs would result in a sort volume of ∼1 L and an EV concentration of 10^6^ ml^−1^. As a consequence, a sorted EV sample requires concentrating before downstream applications can be applied. Dilution by sorting is more problematic for EVs than for cells, because EVs are more difficult to concentrate without loss. In addition, the volume of an EV is on average a million‐fold lower than the volume of a cell, meaning many more EVs must be sorted to exceed the detection limit of downstream analyses.

Sorting of particles is a balance between speed, purity, and sensitivity. Concentrated samples can be sorted faster than diluted samples because more droplets will contain a particle. However, for concentrated samples the probability of co‐sorting particles that are below the lower LoD also increases (Kormelink et al., 2016; Morales‐Kastresana et al., 2019), particularly because the lower LoD of commercial FCM sorters is >100 nm for EVs and the concentration of particles with a diameter <100 nm increases with decreasing diameter (Figure 2B‐C). Moreover, assessing the purity of post‐sorted EVs by reanalysis is less straight forward than for cells as particles below the LoD remain undetected. The speed and sensitivity are further affected by the fluid pressure and the nozzle size (van der Vlist et al., 2012). Generally, increasing the nozzle size and decreasing the fluid pressure improve the sensitivity, but also leads to an increased droplet volume, a more diluted sample, and a decreased throughput. The optimal balance between purity, sensitivity and throughput therefore requires fine‐tuning of the nozzle size and fluidic pressure.

Along with the settings, the purity of a sort is directly contingent on the ability to resolve a population. As with both cells and EVs, sorting of different populations can only be achieved if the fluorescence and/or light scattering signals of said populations can be clearly resolved and gated from one another. Gates that are too close, overlapping with background, or another population can lead to low purity in the sorted population. On the other hand, stringent gate settings can lead to a low sort yield. In the case of EVs, only the fraction of EVs exceeding the LoD can be sorted and enriched.

Due to the many variables involved in FCM sorting, the use of calibrated axes (Section 6.3) is desirable for reproducibility. Some older sorters, however, still use analogue log amplifier electronics (see Section 2.3.3). This results in non‐linear scaling and inaccurate calibrations, especially when signals from reference particles need to be extrapolated for the quantification of dim signals. The use of non‐linear analogue log amplifiers is therefore not recommended for small particle analysis.

#### 2.4.2 Imaging flow cytometers

The main distinction between imaging and traditional flow cytometers is the way optical signals are detected. Conventional flow cytometers focus optical signals originating from a particle onto a single detector. Imaging flow cytometers make an image of each particle by focusing the light onto an array of detectors. This array of detectors typically are time delay integration charge‐coupled devices (TDI‐CCD), which are designed to detect moving objects at low light levels (Han et al., 2016). A TDI‐CCD essentially follows a moving particle throughout the entire field‐of‐view before reading out the signals, which increases the S/N compared to traditional charge‐coupled devices.

The main advantage of an imaging flow cytometer is that particles larger than the diffraction limit, such as single cells, can be imaged. The diffraction limit defines the maximum possible spatial resolution of a lens. Most EVs are below the diffraction limit and hence imaging EVs is not possible. Similar to nanoparticle tracking analysis (NTA), imaging flow cytometers visualize EVs as diffraction limited spots. Consequently, serial dilution controls are required to confirm detection of single particles (Section 5.7). While the fluorescent sensitivity of imaging flow cytometers is higher than traditional flow cytometers, the light scatter sensitivity of imaging cytometers is currently limited and precludes detection of non‐fluorescent 100 nm polystyrene beads.

##### 2.4.2.1 Signal processing

With imaging FCM, each fluorescence or light scattering signal originating from a particle is measured by multiple pixels, which causes notable differences in signal processing between imaging FCM and traditional FCM. To quantify the signal amplitude, signals need to be integrated over multiple pixels, which requires the selection of a region of interest (ROI) around the particle. To add to the complexity, each pixel of the TDI‐CCD has its own response and noise characteristics. Therefore, dynamic imaging algorithms are applied to identify particles, determine the ROI, and quantify the signal amplitude of a particle, which involves assumptions regarding the nature of the signal, noise, and particle dimensions. Based on the working principle of imaging flow cytometers, five technical issues can be anticipated.

First, the dynamic imaging algorithms used to trigger event acquisition, together with the individual pixel response and noise characteristics decrease the precision with which the lower LoD of light scattering or fluorescence detection can be determined compared to traditional FCM. Consequently, it is more difficult to determine which fraction of the EVs exceeds the lower LoD and hence data reproducibility is impeded. Second, dynamic ROI determination leads to variation in the included background noise, which may cause a non‐linear response between the actual signal amplitude and the measured signal amplitude at low signal levels (Görgens et al., 2019). Such a non‐linear response would impede the accuracy of measured signal amplitudes as well as the validity of calibrations. Third, the dynamic range of TDI‐CCD cameras is orders of magnitude lower than the dynamic range of the PMTs used in traditional FCM. The calibration of an imaging FCM may need to be performed at lower laser powers than used for EV analysis, especially because commercially available reference particles are much brighter than EVs (Section 6.3.1.2). Fourth, imaging flow cytometers have co‐aligned lasers such that cells are illuminated with different lasers at the same time to avoid any discrepancies in cell orientation^14^. However, the use of co‐aligned lasers reduces dexterity of fluorophore selection, because signals of fluorophores with a different excitation peak but a similar emission spectrum cannot be distinguished. Fifth, imaging flow cytometers generally operate at a lower flow rate than most traditional cytometers. When used with high magnification lenses, the flow rate can be <1 μl min^−1^ (Görgens et al., 2019). Flow cytometers running at relatively low flow rates require a higher EV concentration than traditional cytometers, which may be a limitation with sample sources that have a low EV concentration.

In summary, imaging FCM may provide higher fluorescence sensitivity and resolution than traditional FCM, but the signal processing is notably different. Further research is needed to investigate and quantify the impact of the anticipated technical issues of imaging FCM. Although technical solutions may be available to address these issues, these solutions are not implemented yet because similar to traditional flow cytometers, imaging flow cytometers are optimized for cell detection. Hence it is important that assays employing imaging FCM implement the appropriate calibrations (Section 6.3), experimental design (Section 3.1), and assay controls (Section 5).

#### 2.4.3 Spectral flow cytometers

The main distinction between spectral flow cytometers and traditional flow cytometers is the way in which optical signals of different wavelengths are spectrally separated before detection. Traditional flow cytometers use spectral filters to direct light within selected wavelength bands, which ideally are fluorophore specific, to different detectors (Section 2.3.2.3). The first commercial spectral flow cytometer introduced by Sony Biotechnology used a spectrometer^15^ instead of spectral filters to evenly split optical signals with a wavelength ranging from 420 to 800 nm across 32 detectors. Newer implementations of the Sony instruments now have up to 188 detectors. Some flow cytometers, such as the Cytek Aurora, split the spectrum in unequal parts, with larger wavelength bands overlapping with the emission peaks of common fluorophores. This implementation has the advantage of increasing the S/N of fluorophores whilst still detecting the spectrum.

The advantage of measuring the full spectrum is that fluorophores with similar peak emissions, but different emission spectra, can be measured simultaneously. These spectral signatures can be separated using spectral unmixing. Spectral unmixing is a mathematical procedure in which a measured emission of the combined spectrum of multiple fluorophores is deconvoluted into individual signatures in a process of spectral decomposition. A prerequisite is that the spectra of each fluorophore is known a priori. Due to spectral unmixing, spectral flow cytometers are particularly suitable for the detection of samples stained with multiple different fluorophores. One potential drawback to the spectral platform is a decreased S/N because the signal originating from each fluorophore is split and measured by multiple detectors, adding noise to the measurement. Like traditional flow cytometers, the utility of spectral FCM remains limited by the small surface area and thus dim signals of EVs. The advantages and disadvantages of spectral FCM approaches to EV research is currently being explored.

### 2.5 Flow cytometer characterization and calibration

Before designing and analysing an EV experiment (Chapter 3.1), it is good practice to characterize the performance of the flow cytometer(s) that are accessible to use. Characterizing the performance of a flow cytometer in a quantitative manner allows selection of the optimal instrument for an EV assay. Characteristics that should be assessed include the dynamic range and sensitivity of each detector as well as the range of particle concentrations that can be measured. The range of particle concentrations that can be measured will determine the minimum sample dilution and the capability of an instrument to detect rare EVs. The lower and upper detection limit of the concentration can be assessed with the buffer only controls (Section 5.1) and by the serial dilution controls (Section 5.7), respectively.

Calibration is required to determine the dynamic range and sensitivity of a detector because the signals collected from a flow cytometer are in arbitrary units and specific to each instrument. The process of calibration involves the measurement of a reference material (i.e., beads) that is used to relate arbitrary units of measurement to standard units, that is, the number of fluorescent molecules, the number of photons, the particle diameter, or the scattering cross section (Section 6.3). Upon calibration, the dynamic range and sensitivity of a detector can be expressed in standard units, allowing for cross‐comparison with other FCM platforms. Furthermore, these quantitative metrics enable assessment and monitoring of instrument performance over time. Importantly, instrument calibration using validated reference materials for instrument characterization allows for the expression of measured EV signals in standardized, and therefore comparable units (Chapter 7).

## 3 EXPERIMENTAL DESIGN AND PRE‐ANALYTICAL PROCEDURES

### 3.1 Experimental design

**FIGURE 10:**
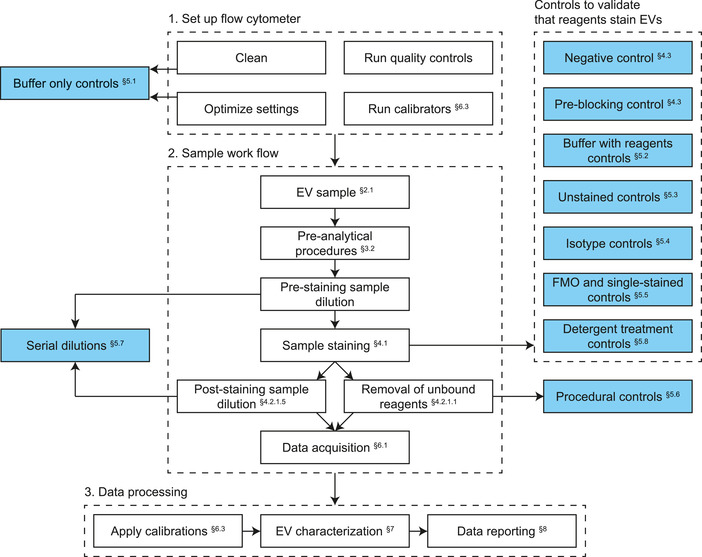
Generic experimental design of a flow cytometry experiment to characterize single extracellular vesicles (EVs). Assay controls are marked in blue. FMO: fluorescence minus one.

While a consensus method for experimental design is likely not feasible, several steps can be taken to improve the interpretability and reproducibility of EV FCM data. To explain the purpose of each step in the experimental design, Figure 10 shows a generic experimental design of a single EV FCM experiment. The main steps can be divided into (1) setting up the flow cytometer, (2) preparing and measuring the samples, and (3) processing and reporting the data. Assay controls are marked in blue and aim to verify that a given step in the experimental design is successful. Without these assay controls, numerous artifacts that can masquerade as EVs may sneak into the data unintentionally. In the next chapters, all steps and assay controls are discussed in the same order as in the MIFlowCyt‐EV reporting framework (Welsh et al., 2020; Welsh et al., 2021).

Figure 10 outlines fundamental experimental design choices, including the dilution of samples prior to staining, the dilution of samples after fluorescent staining or removing unbound reagents with an EV purification method, and selecting the applicable assay controls. Other important decisions include the choice of the trigger detector (Section 6.1.1), whether to measure a sample for a set number of events, time, or volume, and, if required, which pre‐analytical procedures should be used to collect, handle, store and eventually concentrate and/or isolate EVs.

### 3.2 Pre‐analytical procedures

The results of an EV FCM experiment are strongly influenced by how the sample itself was collected, handled, stored, and processed prior to measurement. These pre‐analytical procedures influence downstream assays and have led to variability in data within the field. While there are no gold standards for these pre‐analytical procedures, it has become clear that detailed reporting of experimental variables improves reproducibility. This insight has led to a comprehensive body of literature on pre‐analytical variables, notably EV‐TRACK, MIFlowCyt‐EV, MISEV, and position papers for specific assays (Coumans et al., 2017; Lötvall et al., 2014; Mateescu et al., 2017; Théry et al., 2018; van der Pol et al., 2022; Van Deun et al., 2017; Welsh et al., 2020). The main commonalities of these efforts are the emphasis on transparency in reporting and the recognition that pre‐analytical variables may bias downstream data.

Details on sample collection, handling and storage in the context of EV research will not be discussed here, but could be readily assessed in the literature (Lötvall et al., 2014; Théry et al., 2018; van der Pol et al., 2018; Van Deun et al., 2017). While EV FCM generally does not require sample purification and/or concentration, EV purification may be needed to remove unbound reagents, such as fluorescent antibodies, as well as reagents added prior to staining, such as anticoagulants and additives to cell culture media (e.g., phenol red). The next section briefly explains four frequently used methods of EV purification.

#### 3.2.1 Ultracentrifugation

Concentration and purification of EVs by ultracentrifugation follows the principle of sedimentation by centrifugal force, where particle separation is based on differences in size and density (Rikkert et al., 2018). Larger particles with higher density than the suspension fluid migrate away from the axis of the centrifuge towards the pellet, while smaller and less‐dense particles migrate toward the axis of the centrifuge and remain in the supernatant. The aim of ultracentrifugation is to pellet down EVs, while remaining unbound reagents in the supernatant. The advantages of ultracentrifugation are that multiple samples can be processed in parallel, and that the procedure is easy to learn and apply. Some disadvantages of this method include: limited enrichment and recovery (Geeurickx et al., 2019; Görgens et al., 2019; Jayachandran et al., 2012; Ludwig et al., 2018; Rikkert et al., 2018), formation of aggregates, and destruction of EVs (Linares et al., 2015). This can result in a reduction and alteration of EV characteristics. Reported recovery ranges of EVs isolated by ultracentrifugation range from 5% to 80% (Geeurickx et al., 2019; Görgens et al., 2019; Jayachandran et al., 2012; Ludwig et al., 2018; Rikkert et al., 2018). The large discrepancy in the reported recoveries can in part be explained by the differences in the sensitivity of the flow cytometers used to measure EVs. The smaller the detected EVs, the lower the quantified recovery, because smaller EVs spin down with lower efficiency. Another disadvantage of ultracentrifugation is that micelles originating from generic fluorescent staining (Section 4.1) may co‐isolate with EVs (Dominkuš et al., 2018). The use of ultracentrifugation requires expensive specialized equipment and has limited scalability due to the number of samples that can be processed simultaneously.

#### 3.2.2 Density gradient centrifugation

Density gradient centrifugation is a method to separate particles based on their density and can therefore be used to separate EVs from non‐EV particles or separate different types of EVs (der Vlist et al., 2012). With density gradient centrifugation, a centrifugation tube is filled with layers of liquid having a density that increases in density from the top to the bottom of the tube. In the EV‐field, frequently used substances to create layers of different density are iodixanol and sucrose (Aalberts et al., 2012). Besides equilibrium centrifugation, differences in sedimentation velocity can also be used to separate particles based on both mass density and size (Cantin et al., 2008). Disadvantages of density gradient centrifugation are that the procedure takes multiple hours and requires specialized and expensive equipment. Moreover, limited numbers of samples can be processed at the same time and the recovery of EVs is variable and substantially lower than some of the other methods described in this compendium (Geeurickx et al., 2019).

#### 3.2.3 Size exclusion chromatography

Size‐exclusion chromatography (SEC) fractionates by size (Lötvall et al., 2014; Mol et al., 2017). With SEC, the sample is loaded onto a column containing a porous material, which is called the stationary phase. The stationary phase typically consists of agarose beads with an effective size cut‐off of 35 or 70 nm (Böing et al., 2014; Monguió‐Tortajada et al., 2019). The buffer running through the column is called the mobile phase. Particles larger than the pores of the stationary phase will pass directly through the column, whereas smaller particles will enter the pores, get delayed and elute in later fractions. An advantage of SEC is the application of low forces compared to ultracentrifugation, resulting in significantly less shear stress, damage, and aggregate formation. In addition, SEC takes a few minutes whereas ultracentrifugation and density gradient centrifugation take one or more hours. SEC with sepharose 2B has also been shown to have a high recovery of up to 80% for EVs >70 nm (Corso et al., 2017; Monguió‐Tortajada et al., 2019). Disadvantages of this technique include the dilution of the sample and co‐elution of EVs with certain reagents, such as micelle‐forming lipophilic dyes (Dominkuš et al., 2018).

#### 3.2.4 Ultrafiltration

Ultrafiltration can separate unbound reagents from EVs using filters designed for concentration of proteins ranging in molecular weight from 10 to 750 kDa. With centrifugal ultrafiltration, samples are loaded on top of a filter, which is placed in the centre of a centrifugation tube. By applying an acceleration force by centrifugation, particles smaller than the filter pore, such as unbound reagents, flow through the filter while the EVs are retained. The EVs remaining on top of the filter are concentrated and can be resuspended in the buffer of choice. With tangential flow filtration, the sample flows tangentially across the surface of the filter, where particles smaller than the pore are being trapped (Busatto et al., 2018). At the end of the filter, the filtered sample is released.

Advantages of ultrafiltration are that it can be performed with conventional centrifuges and with high recovery (up to 80%) (Lobb et al., 2015). Disadvantages include the formation of protein and EV aggregates, which can lead to filter clogging, binding of EVs to the filter (Davies et al., 2012; Kornilov et al., 2018; Lai et al., 2010; Lobb et al., 2015; Rood et al., 2010), and disruption of EVs due to shear stress (Cvjetkovic et al., 2014). Filter clogging may be overcome by performing tangential flow filtration instead of direct flow filtration. Similar to ultracentrifugation and SEC, ultrafiltration does not warrant removal of micelles formed by lipophilic dyes.

## 4 SAMPLE PREPARATION

Sample preparation is a prerequisite for successful FCM experiments. Aside from the standard best practices recommended for FCM analysis of cells, additional considerations need to be taken when preparing EV samples for analysis. This chapter covers the basics of EV sample preparation, including a discussion on fluorescent reagents and their selection, procedures for staining, options for the removal of unbound reagents, and serial dilution of samples for analysis.

### 4.1 Fluorescent staining

Fluorescent staining allows for the phenotypic analysis of EVs through identification of specific cellular biochemical components, such as proteins, lipids, and nucleic acids present in or associated with EVs (Figure 2A). Fluorescent staining can thereby be used to differentiate EVs from non‐EV particles and to elucidate the biology and function of EVs.

Compared to a cell, an EV has a substantially smaller surface area and volume. This physical constraint results in proportionately fewer available target molecules for staining, and therefore stained EVs display orders of magnitude lower fluorescence signals than stained cells (Figure 2D). The fluorescence signals of EVs are typically close to or below the lower LoD of the fluorescence detectors, making the detection of an entire population of stained EVs difficult or infeasible. For example, the fluorescence signals of stained EVs may overlap with the background noise of the flow cytometer, autofluorescence signals from unstained EVs and non‐EV particles, or fluorescence signals from unbound reagents. To optimize the S/N, it is imperative that the selected reagents and applied procedures are thoroughly validated and optimized for EV applications.

Fluorescent reagents that are typically used for staining EVs are antibodies conjugated with fluorophores (Section 4.1.1) and generic membrane or luminal stains (Section 4.1.2). Alternatively, EVs can be stained using nucleic acid stains (Section 4.1.2.2) or genetically engineered to express fluorescent proteins (Section 4.1.3).

#### 4.1.1 Immunofluorescence staining

**FIGURE 11:**
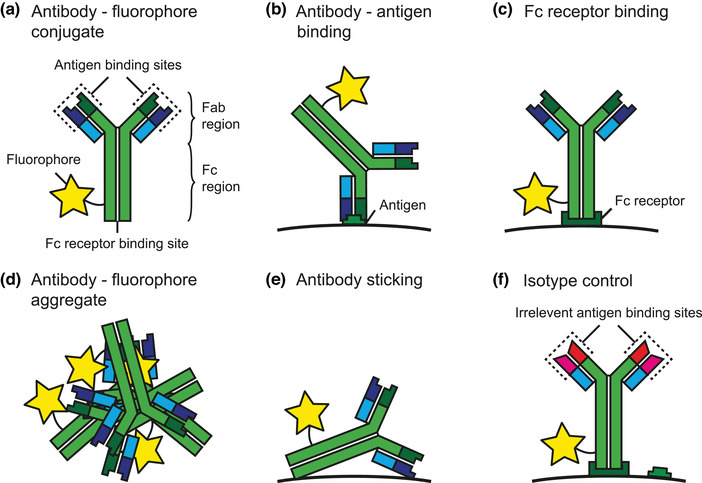
Schematic representation of a fluorescent antibody conjugate and processes involved in antibody staining. (A) Fluorophore conjugated to an antibody. The antibody consists of two antigen‐binding sites (Fab region) and an isotype specific fragment to bind to Fc receptors (Fc region). (B) The antigen binding sites allow the antibody to bind specifically to antigens exposed on the surface of an EV. (C) The isotype specific fragment binds to isotype‐specific Fc receptors that may be exposed on the surface of an EV. Therefore, antibodies do not bind specifically to antigens, but also to Fc receptors. (D) Fluorescent antibody conjugates, antibodies and fluorophores may form aggregates with similar dimensions as EVs. (E) Antibodies may stick to the surface of EVs or other particles. (F) Isotype control antibody with the same Fc regions (e.g., IgG1 and IgG2a) as antibodies used to stain EVs, but with a Fab region that recognizes irrelevant antigens. Isotype control antibodies therefore allow assessment of the degree of Fc receptor‐mediated binding.

The most common fluorescence staining method in FCM utilizes antibodies conjugated with fluorophores (Saper, 2009). Antibodies allow for the targeted labelling of specific protein antigens. Figure 11(A) schematically depicts a fluorescent antibody conjugate. Structurally, the antibody consists of two antigen‐binding fragments (Fabs) and an isotype specific or crystallizable fragment (Fc). As the name suggests, the Fabs contain the antigen binding sites and allow the antibody to bind specifically to antigens exposed on the surface of EVs, as shown in Figure 11(B). The Fc region is identical in all antibodies of a common isotype, and binds to isotype‐specific Fc receptors that can be present on EVs, as shown in Figure 11(C). Although cells may bind sufficient Fc receptors to allow for detection, Fc receptor binding is likely less prominent for EVs than for cells under the assumption that the expression level scales proportionally to the surface area of the particle. To substantiate this statement, Figure 2(C) shows the indicative number of IgG‐Fc receptors of platelet‐derived EVs, assuming that the IgG‐Fc receptor density of platelets and platelet‐derived EVs are equal and that the same fraction of receptors is stained. On flow cytometers with an LoD lower than 100 PE molecules, the IgG‐Fc receptors would remain undetectable for EVs smaller than 580 nm.

Antibodies can be conjugated with one or multiple copies of a fluorophore. The average number of copies of a fluorophore that is conjugated with an antibody is defined as the fluorophore to protein ratio (F/P). Reagents should have a specified F/P of at least 1. An F/P <1 indicates that not every antibody molecule within a reagent is conjugated to a fluorophore. Hence, conjugated and unconjugated antibody molecules are competing for the same antigen, resulting in a decrease in fluorescence intensity, because unconjugated antibodies do not emit fluorescence. An F/P ≥1 neither guarantees the absence of unconjugated antibody molecules, because the F/P is an ensemble average. However, the probability of unconjugated antibody molecules is lower for F/P ≥1 than F/P<1. The F/P of commercially available antibodies is optimized for cell detection. Antibodies with an F/P specifically devoted to EV applications are warranted in the future.

Most antibodies used in FCM are monoclonal. Monoclonal antibodies are derived from a single cell line and bind specifically to a single epitope within an antigen. An epitope is the part of an antigen that is recognized by the antibody. Antibodies from a different clone targeting the same antigen may recognize different epitopes and may have different binding affinities. Polyclonal antibodies are derived from different cell lines and may target multiple antibodies or a single antibody, but different epitopes. Polyclonal antibodies usually have the same isotype and are commonly used as secondary antibodies in indirect immunofluorescence staining (Section 4.1.1.1). Monoclonal antibodies result in most consistent staining and are therefore recommended for EV FCM.

Antibody reagents require careful selection, due to the wide variety of different antibody reagents with the same target that are commercially available (Xie, 2017). It is recommended to report catalogue and lot numbers consistently (Welsh et al., 2020). Before starting large and long‐lasting studies, it is further recommended to test different antibody clones to the same antigen in order to confirm reproducibility (Böttcher et al., 2019). With antigens that are not well‐known, it is preferable to demonstrate specificity by using a cell line that does and does not expresses the antigen.

##### 4.1.1.1 Direct and indirect immunofluorescence staining

With direct immunofluorescence staining, antigens are stained with an antibody that is conjugated with one or more fluorophores. The antibody recognizes and binds to the antigen and can be detected by fluorescence. In practice, direct immunofluorescence staining is performed with a mix of different antibodies targeting different antigens. Each antibody type in a mix is conjugated with a different fluorophore to enable identification of the antibody type.

With indirect immunofluorescence staining, antigens are stained with a primary antibody that is not conjugated with fluorophores. In turn, the primary antibody is stained with secondary or even tertiary antibodies conjugated to fluorophores to amplify the detection of the primary antibody. Indirect staining has historically been developed for staining of cells but is unsuitable for high parameter analyses. Therefore, indirect staining of cells is nowadays not commonly used. Although signal amplification is appealing, indirect staining is not recommended for EV research for the following reasons.

When indirect immunofluorescence is used to stain cells, unbound primary antibodies are washed away to reduce binding competition with the secondary antibody and reduce the concentration of required secondary antibody. However, as the removal of unbound antibodies from EV samples is difficult (Section 3.2), the indirect staining procedure is more complicated for EVs than for cells. Additionally, the increase in the number of successive incubations extends the time of the staining procedure and may contribute to the loss of EVs, because EVs can stick to the tubes or plates used for staining. Each additional antibody included in the staining procedure warrants additional assay controls (Section 5) to interpret the data. The additional antibodies may also increase the background noise level, contribute to swarm detection (Section 2.3.3.4) and lead to cross‐linking of EVs. Furthermore, indirect staining negates the ability to report on antigen density (Section 7.3), because the secondary or tertiary antibodies that are conjugated to the detected fluorophores do not directly target the antigen of interest.

##### 4.1.1.2 Antibody alternatives

A newer class of antibodies and antibody alternatives that allow for antigen‐specific targeting include nanobodies and aptamers. Aptamers are oligonucleotides molecules that specifically bind to a molecular target. Nanobodies, or single‐domain antibodies, are fragments of camelid antibodies that contain only a single Fab region. As aptamers and nanobodies can be conjugated with fluorophores and bind specifically to antigens, they may become a viable alternative to conventional antibodies for staining (Bruce & McNaughton, 2017). Especially the small size compared to conventional antibodies and the lack of Fc receptors while retaining specificity of binding to targeted antigens are attractive properties of aptamers and nanobodies for EV FCM applications (Bruce & McNaughton, 2017). However, the utility of these new staining alternatives for EV FCM is yet to be demonstrated.

##### 4.1.1.3 Fluorophore selection

The selection of fluorophores for an EV FCM experiment needs to be in concordance with the detection capabilities of the flow cytometer that will be used and involves three steps. First, the combination of detectors and fluorophores resulting in the most sensitive detection should be identified. Second, reference materials should be available to calibrate the detectors corresponding to the selected fluorophores. Third, when different fluorophores are simultaneously used, *spectral spillover*, which is the overlap between the *emission spectra* of different fluorophores, should be avoided. Although algorithms exist to deconvolute spectrally overlapping signals, fluorescence spillover leads to a reduction in sensitivity (Nguyen et al., 2013), which is undesirable for EV FCM where signals are already dim (Section 2.1.1).

The first step of fluorophore selection involves the identification of the most sensitive detectors and brightest fluorophores. To identify the most sensitive detectors, the LoD of each detector needs to be determined, which can be achieved through instrument characterization (Section 2.5). The brightness of fluorophores was extensively discussed in Sections 2.2.2.1 to 2.2.2.3. Small fluorophores are usually dimmer compared to large fluorophores, but smaller fluorophores allow for a higher *F/P* when conjugated to antibodies (Chattopadhyay et al., 2012; McKinnon, 2018; Schwarze et al., 1983). Whether larger fluorophores cause more problems with staining EVs compared to cells, depends on the surface density and mobility of antigens in the membrane, and is a subject that requires further investigation. Table 4 provides an overview of the properties of commonly used fluorophores and available reference materials.

##### 4.1.1.4 Types of fluorophore

Fluorophores that are used in FCM can be divided into four classes: (1) large protein molecules, (2) small organic molecules, (3) organic polymers and (4) inorganic fluorescent nanocrystals (Chattopadhyay et al., 2012).

Large protein molecules and small organic molecules were one of the first fluorophores to be used in FCM. An example of large protein fluorescent molecules are phycobiliproteins, such as *APC* and PE, which range in molecular mass between ∼100 and 300 kDa (see Table 4). Examples of small organic molecule fluorophores are the cyanine dyes and FITC, which is a derivative of fluorescein and has a molecular mass of 0.4 kDa. Many new dyes have been developed from these original large protein and small organic molecules.

Organic polymer dyes are diode polymers whose brightness increases with the length of the polymer. Organic polymer dyes have a higher extinction coefficient, quantum yield and brightness than large protein and small organic molecules. An example of an organic polymer dye is Brilliant Violet, which has a molecular mass between 7 and 80 kDa. The molecular mass of Brilliant Violet is thereby similar to large protein molecules. Organic polymer dyes might be prone to aggregation and require specialized buffers to maintain stability. Caution should therefore be taken when organic polymer dyes are used to stain EVs, because aggregates could result in false positive events.

The most common type of inorganic fluorescent nanocrystals are quantum dots (QDs). QDs are nanocrystals of a fluorescent semiconductor material that typically range 10 nm to 20 nm in diameter (Bera et al., 2010), which is large relative to the size of EVs. The brightness of QDs is on par with the brightest conventional fluorophores. QDs are resistant to photobleaching, have a narrower emission spectrum than conventional fluorophores, and are optimally excited in the UV range. Similar to conventional fluorophores, QDs can be conjugated to antibodies, aptamers or nanobodies. Disadvantages of QDs are that they (1) are large relative to the size of EVs, (2) are difficult to conjugate to antibodies, (3) have limited stability when conjugated, (4) have a wide excitation spectrum, (5) are polyvalent^16^ when conjugated and therefore capable of capturing more than one EV, and (6) depending on their composition, QDs may substantially increase the buoyant density of stained EVs, thereby affecting density gradient centrifugation procedures. Newer types of inorganic fluorescent nanocrystals include polymer dots, trademarked as the StarBright dyes (Chen et al., 2017; Jin et al., 2012). Polymer dots are brighter, but also larger than QDs. For EV staining applications, polymer dots have the same usage concerns as QDs.

##### 4.1.1.5 Tandem dyes

Tandem dyes are created through covalent linkage of a donor and an acceptor molecule. Excitation and emission of light by a tandem dye follow the principles of FRET (Section 2.2.2.2). The donor molecule is called the base dye. A single base dye can be linked to multiple acceptor molecules, which may contribute to variation between production lots. Tandem dyes may consist of a large protein fluorophore linked to small organic molecules, such as PE‐Cy5 and APC‐Cy7. Alternatively, the base dye could be an organic polymer dye. For example, BV421 and BV510 are base dyes, while other available Brilliant Violet dyes are tandem dyes.

For EV FCM, base dyes are preferred over tandem dyes, because base dyes are smaller than tandem dyes and because to the best of our knowledge fluorescence calibration materials, such as beads with an assigned number of molecules of equivalent soluble fluorophores (MESF), are unavailable for tandem dyes. MESF is a common standard unit of fluorescence intensity that can be derived by performing a calibration (Section 6.3.1). Fluorescence calibration with tandem dyes can be performed using antibody capture (ABC) beads (Section 6.3.1).

##### 4.1.1.6 New fluorophore developments for EV FCM

New commercially available fluorophores are continuously being introduced, yet few of these new fluorophores are a result of new chemistry. Instead, these fluorophores are a re‐branding of existing fluorophores with slight modifications. New fluorophores with distinct excitation and emission spectra and different brightness are often developed with high parameter cell‐based assays in mind. As a result, most new fluorophores do not satisfy the criteria for use in EV FCM. Meanwhile the field is awaiting new bright base dyes that are substantially smaller than EVs. Manufacturers can contribute to the improvement of reproducibility and standardization by providing the F/P, tandem ratio, and antibody concentration of existing reagents for EV FCM. In addition, each newly released fluorophore should be accompanied with reference materials to perform an MESF calibration corresponding to the fluorophore.

##### 4.1.1.7 Other fluorophore considerations

The emission and excitation spectra and fluorescence intensity of a fluorophore are affected by the chemical environment (Sections 2.2.2.1). Generally, conjugation of fluorophores to antibodies as well as the close proximity of lipids, proteins, soluble molecules, membranes, and other antibody fluorophore conjugates may lead to quenching (Section 2.2.2.2) and thereby to a reduction in fluorescence intensity. The probability of quenching increases with increasing antigen densities, but solid experimental data on the antigen density of EVs is currently lacking and therefore this topic requires further investigation.

#### 4.1.2 Generic fluorescent staining

Generic fluorescent staining, defined here as any form of non‐immunofluorescence staining, stain a specific type of molecule, such as lipids, amine groups, or nucleic acids. Generic fluorescent staining is often used with the purpose of labelling and detecting all, and preferably only, EVs. However, a generic stain that is capable of staining all and only EVs does not yet exist (de Rond et al., 2019; de Rond et al., 2018) and is an area of potential research for new dye developments. A legitimate reason to use generic stains is to enable fluorescent triggering (Section 6.1). Depending on the flow cytometer and staining procedure (de Rond et al., 2018), fluorescent triggering can improve the S/N, thereby enabling detection of smaller EVs compared to scatter triggering (Arraud et al., 2016).

##### 4.1.2.1 Membrane and amine reactive dyes

Many of the generic stains available are lipid membrane dyes, plasma membrane dyes or amine reactive dyes. Lipid and plasma membrane dyes bind to or incorporate into the EV membrane. Examples include 4‐(2‐[6‐(dioctylamino)‐2‐naphthalenyl]ethenyl)‐1‐(3‐sulfopropyl)pyridinium (di‐8‐ANEPPS), BODIPY TR ceramide, CellMask Stains, lipophilic carbocyanine dyes, MemBright, and Paul Karl Horan (PKH) dyes (de Rond et al., 2019; de Rond et al., 2018; van der Vlist et al., 2012). Amine reactive dyes target the amine groups on proteins within the EV lumen and depend on the presence of esterases for cleavage and subsequent activation. Examples of amine reactive dyes include calcein acetoxymethyl ester (CA), calcein CA violet, and carboxyfluorescein succinimidyl ester (CFDA‐SE) (Collot et al., 2019; Johnson et al., 2020; Lubart et al., 2020; Morales‐Kastresana et al., 2019; Stoner et al., 2016).

The intended application for many generic stains is to identify specific membrane structures within cells in fluorescence imaging. When selecting a generic stain for use in EV FCM, four factors should be taken into consideration. First, the presence and staining efficiency of non‐EV particles may affect the staining efficiency and detection of EVs. Second, fluorescent lipophilic membrane dyes may form micelles or aggregates, which may cause false positive events when used for EV staining (Morales‐Kastresana et al., 2017; van der Vlist et al., 2012). Although membrane dyes based on zwitterionic anchors could be used to reduce aggregate formation (Collot et al., 2019), generic stains typically require removal of unbound reagents and fluorescent non‐EV particles (Section 3.2). Third, EVs are smaller and therefore dimmer than cells (Section 2.1.1), also after generic fluorescent staining. Fourth, EVs may have a reduced ability to efficiently intercalate membrane dyes compared to cells, depending on the lipid and protein composition of EVs as well as on the chemical composition of the dye. Improved data reporting is necessary to generate evidence‐based recommendations for the selection of generic fluorescent stains (Welsh et al., 2020).

When generic stains are used for quantitative fluorescence analysis, careful data evaluation is required, because the binding or insertion efficiency and the fluorescent properties of generic stains depend on the composition of EVs. Therefore, the measured fluorescence intensities do not only reflect differences in EV size but can also reflect differences in EV membrane composition (Lubart et al., 2020).

##### 4.1.2.2 Nucleic acid stains

Fluorescent nucleic acid stains are available to stain different types of nucleic acids in different ways. For example, nucleic acid stains for DNA are capable of intercalating in between base‐pairs, across base‐pairs (bis‐intercalator), across major and minor grooves of the DNA, and across the DNA backbone. Many available DNA stains are not specific for DNA and also emit fluorescence when bound to RNA, although the emitted fluorescence intensity is usually dimmer (Liu et al., 2022). Whereas nucleic acid stains have been developed to stain all nucleic acids, molecular beacons are developed to label specific sequences (Tan et al., 2004). These molecular beacons only emit fluorescence when bound to the target nucleic acid sequence.

To date, there is only one study demonstrating the detection of nucleic acids in or at particles within the EV size range on a calibrated flow cytometer (Liu et al., 2022). These experiments have been performed on lab‐built flow cytometers that are capable of detecting <10 fluorophores. With a commercial flow cytometer and currently available nucleic acid stains, however, it is unlikely that nucleic acids can be detected within EVs^17^. The utility of publications wherein a commercial flow cytometer was used to detect nucleic acid stains in EVs is limited, because a lack of calibration and assay controls hampers data interpretation.

Regarding assay controls, nucleases should be used after nucleic acid staining to demonstrate that signals are (1) specific to the nucleic acid of interest and (2) not derived from external sources (Liu et al., 2022). However, nuclease treatment of nucleic acids in the lumen of EVs requires membrane permeabilization to demonstrate signal abolition.

#### 4.1.3 Genetic expression of fluorescent proteins

As an alternative to staining, EVs can become fluorescent by genetic manipulation of their parent cells (Geeurickx et al., 2019; Görgens et al., 2019; Shen et al., 2018). Cells can load proteins, such as the tetraspanin CD63, into EVs. In order to release fluorescent EVs, cells can be genetically manipulated to bind a fluorescent protein to a protein that is endogenously loaded into (or onto) EVs, such as green fluorescent protein to CD63. During vesicle formation, the cell incorporates the hybrid protein into vesicles and as a result, EVs released by the cell are fluorescent (Görgens et al., 2019; Wiklander et al., 2018).

Endogenous loading of fluorescent proteins into EVs is typically achieved by binding fluorescent proteins to EV sorting proteins, such as the tetraspanins (Corso et al., 2019). An advantage of endogenous loading is that no unbound reagents are present in the medium, because EVs are fluorescent upon release from the cell. However, sub‐populations of released EVs may express different amounts of fluorescent proteins (van der Vlist et al., 2012). Furthermore, non‐physiological concentrations of modified proteins could affect the biology of parent cells as well as the protein composition and function of EVs.

### 4.2 Staining procedure

The procedure for immunofluorescence and generic fluorescent staining of EVs share the same basic principles. In addition, the best practices and recommendations for immunofluorescence and generic fluorescent staining of cells also apply to EVs (Saper, 2009; Weller, 2018), although cell activation, antibody internalization, and death are not a concern when staining EVs.

#### 4.2.1 Staining conditions, considerations and procedure

Fluorescent staining depends on many variables, including the concentration of the reagent, the concentration of the target molecules and thus the concentration of EVs, the exposure of light, the incubation time, the pH of the medium, and the temperature. Many staining reactions are reversible and therefore reach a dynamic equilibrium when the binding and unbinding of reagents occur at the same rate (Van Oss et al., 1986). Distinct from immunofluorescence staining, the binding efficiency of many generic fluorescent stains depends on reagents that aid membrane intercalation, such as dimethyl sulfoxide. When describing immunofluorescence and generic fluorescent staining experiments, all relevant variables need to be reported (Welsh et al., 2020).

To develop a reliable assay with a maximized fluorescence signal, it is important to optimize the concentration of the staining reagent(s), concentration of EVs, and staining conditions. In general, the staining procedure of EVs is as follows: (1) remove aggregates in the reagents, such as aggregates of antibodies and fluorophores, (2) estimate the concentration of EVs in the sample, (3) estimate the optimal concentration of reagents and EVs during incubation by titration, (4) determine the optimal incubation time and temperature, (5) reduce the concentration of unbound reagents associated with fluorescent staining prior to analysis by FCM.

##### 4.2.1.1 Removal of staining reagent aggregates

Staining reagents may contain particles that exceed the trigger threshold, such as antibody‐fluorophore aggregates, as illustrated in Figure 11(D), protein aggregates, or micelles in the case of lipid dyes. Particles originating from the reagent can be reduced in concentration or entirely removed by centrifugation or filtration. For example, the fastest acceleration setting of a benchtop high‐speed centrifuge (e.g., 19,000∙g for 5 min) is often sufficient to remove detectable antibody‐fluorophore aggregates. To confirm that particles in the reagent do not represent false‐positive counts, the buffer with reagents control (Section 5.2) should be used.

##### 4.2.1.2 Estimation of EV concentration

As reversible staining reactions reach a dynamic equilibrium, only a fraction of the target molecules is stained. To ensure that the fraction of stained target molecules is close to one, the staining reaction needs to be saturated, which can be achieved by using “an excess” of staining reagents. The concentration of staining reagents required to reach saturation depends on the dissociation constant of the reaction and the concentration of target molecules. Ideally, the concentration of the target molecules during staining is therefore similar in different samples. However, the concentration of the target molecules in EV samples cannot be accurately determined. Instead, it is good practice to estimate the concentration of EVs as an indication of the concentration of the target molecules. The concentration of EVs can be estimated with FCM or an orthogonal detection method, such as resistive pulse sensing (RPS). To achieve similar EV concentrations in different samples during staining, samples can be diluted based on preliminary EV concentration measurements.

##### 4.2.1.3 Titration of staining reagents

The concentration of staining reagents required to reach saturation is difficult to theoretically predict, because often neither the dissociation constant of the reaction nor the concentration of target molecules is known. In addition, the absence of swarm detection originating from staining reagents needs to be experimentally confirmed. Therefore, titration of the staining reagents is recommended to find the optimal concentration of staining reagents. This recommendation applies to immunofluorescence and generic fluorescent staining.

Titration aims to find the highest concentration of staining reagents that does not result in false‐positive events originating from the reagent, which should be checked with the buffer with reagents controls (Section 5.2). The optimal concentration of staining reagents results in the maximum fluorescence intensities of stained EVs, while keeping the background fluorescence intensity to a minimum (Kalina et al., 2020). The titration should be performed on a predetermined concentration of EVs and a range of 6 to 8 serial dilutions is recommended to find the optimum. The optimal concentration of staining reagents should always be reported in standard units, such as mole L^−1^ or μg ml^−1^, but not in dilution factors.

##### 4.2.1.4 Optimization of incubation time and temperature

The two main conditions that affect staining are the incubation time and temperature. Ideally, the incubation time should equal the time required to reach the dynamic equilibrium. In practice, however, the incubation time is compromised by sample degradation and time restrictions. An increase in temperature will shorten this incubation time, but might induce non‐specific labelling or degradation of the sample and reagents. To prevent bleaching of fluorophores, incubation is performed in a dark environment.

While the aforementioned variables have been extensively tested for antibody staining of cells, the optimal variables for staining EVs are subject to ongoing investigations (Tertel et al., 2020). Moreover, the optimal variables may differ between reagents and EV samples. Therefore, all relevant variables need to be reported when describing the antibody staining procedure (Welsh et al., 2020).

##### 4.2.1.5 Reduction of unbound reagents

After staining, the EV sample will contain unbound reagents. As the presence of unbound reagents may lead to false‐positive counts, the concentration of unbound reagents needs to be decreased after staining either by EV purification methods (Section 3.2) or sample dilution (Section 5.7). There are advantages and disadvantages to both methods. Dilution is a more economical and practical approach than purification and prevents the loss of EVs. However, when dilution does not inhibit false‐positive counts originating from reagents, EV purification is required. EV purification methods can reduce the concentration of unbound reagents, but are often laborious, have a limited and varying EV recovery (Yuana et al., 2015), and may be biased towards certain EV types or sizes (Böing et al., 2014; Rikkert et al., 2018).

An example of a reagent that typically requires purification after staining, are lipophilic dyes. Lipophilic dyes can form aggregates and/or micelles in the size and fluorescence range of fluorescently stained EVs, thereby inducing false‐positive fluorescent counts. These aggregates and micelles can be removed from EVs by density gradient centrifugation (Dominkuš et al., 2018; Hoen et al., 2012; van der Vlist et al., 2012).

After applying EV purification methods or sample dilution, the absence of false‐positive counts originating from reagents should be checked using the procedural controls (Section 5.6) or the buffer with reagents controls (Section 5.2), respectively. EV purification or sample dilution can affect the antibody and EV concentrations and therefore the dynamic equilibrium, meaning that the time between staining and detection may decrease the brightness of stained EVs. Therefore, the time between staining and detection should be investigated, optimized, standardized and reported (Welsh et al., 2020).

### 4.3 Detection of stained EVs by flow cytometry

**FIGURE 12:**
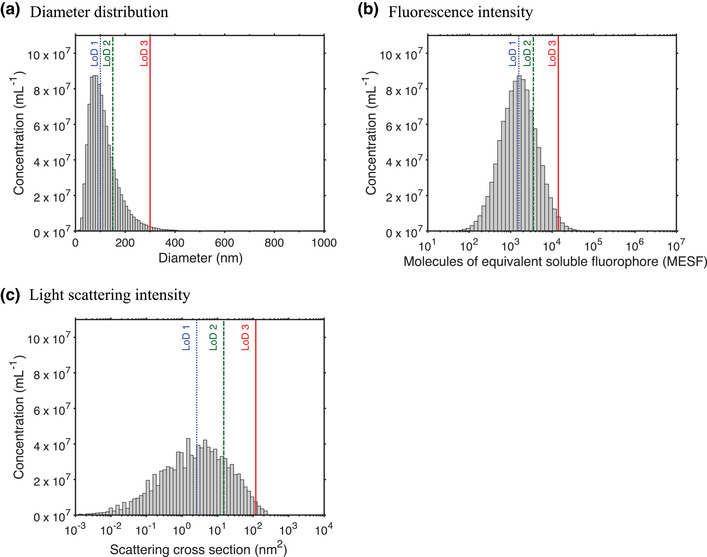
The lower limit of detection (LoD) of a flow cytometer affects the statistical description of the size distribution, fluorescence intensity distribution, and light scattering intensity distribution of an extracellular vesicle (EV) sample. (A) Size distribution, (B) fluorescence intensity distribution, and (C) light scattering intensity distribution of the same population of EVs. The vertical lines denote three LoDs relating to diameters of 100 nm (dotted line), 150 nm (dashed line), and 300 nm (solid line). The effect of these LoDs for statistical descriptions of the size distribution, fluorescence intensity distribution, and light scattering intensity distribution can be found in Tables 4 and 5.

**FIGURE 13:**
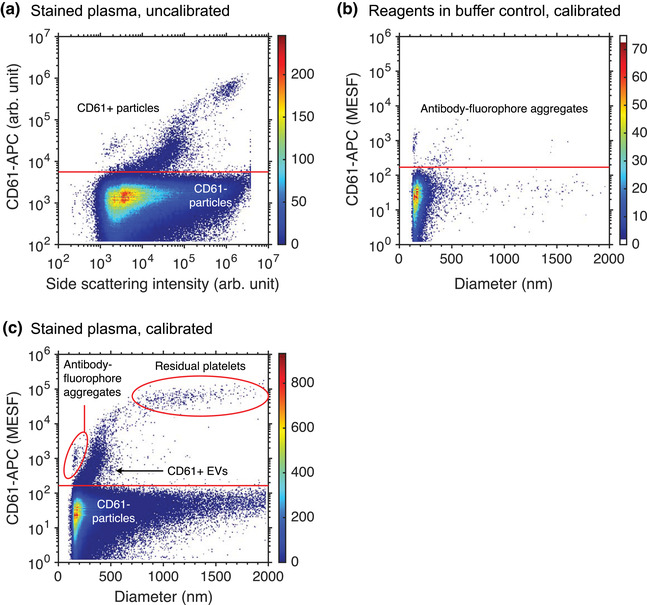
Example of the identification of EVs stained with antibodies using an assay control and a calibration. (A) Measured fluorescence intensity (Apogee A60‐Micro) versus the side scattering intensity of particles in plasma stained with CD61‐APC (Gasecka et al., 2020). The fluorescent gate (horizontal line) differentiates stained particles (CD61+) from background noise and unstained particles (CD61‐). The arbitrary units (arb. unit) preclude identification of the three positively stained populations. (B) Measured fluorescence intensity in standard units versus the diameter of particles in CD61‐APC added to phosphate buffered saline, which was the buffer used in panel A. The data confirms the presence of antibody‐fluorophore aggregates. (C) Measured fluorescence intensity in standard units versus the diameter of particles in the same sample as displayed in panel A. Owing to the calibration and the reagents in buffer control, the three populations can be readily identified as antibody‐fluorophore aggregates, EVs stained with CD61, and residual platelets.

EVs labelled with fluorescently conjugated antibodies or generic fluorescent stains are dim. To illustrate this point, Figure 12(B) shows an estimate of the fluorescence intensity distribution of CD61+ EVs in plasma using the estimated size distribution of EVs in Figure 12(A) and the number of receptors per EV in Figure 2(D). Despite the high density of CD61 compared to most other antigens, fluorescence signals of most EVs are below 1000 MESF. For comparison, residual platelets in fresh whole blood stained with CD61‐APC have a confined fluorescence intensity profile with a median fluorescence intensity (MFI) around 100,000 MESF. The dim signal of fluorescently labelled EVs has two implications for their detection by flow cytometry:

First, the fluorescence signals of stained EVs may be indistinguishable from background noise of the flow cytometer and unstained particles. Although selecting brighter fluorophores (Section 4.1.1.3) and a detector with higher sensitivity will improve the ability to detect stained EVs, a fluorescent gate is required to differentiate stained EVs from unstained EVs and/or non‐EV particles. For example, Figure 13(A) shows the measured fluorescence intensity versus the side scattering intensity of particles in plasma stained with CD61‐APC. The horizontal line indicates the fluorescent gate that differentiates stained particles from background noise and unstained particles. The fluorescent gate may be defined using the unstained controls (Section 5.3), which typically results in lower background fluorescence than the stained samples. Alternatively, fluorescent gates are defined manually based on the scatter plot or automatically based on the histogram of the background fluorescence intensity (Gankema et al., 2022). Hitherto, the distinction between stained and unstained particles remains subjective, which emphasizes the importance of characterizing the performance of the flow cytometer by a calibration (Section 2.5) and reporting fluorescence gates in standard units (Section 6.3.1).

Second, the positive fluorescence signals may originate from non‐EV particles, such as antibody aggregates or cells. For example, Figure 13(A) clearly shows that three distinct particle populations stain positively for CD61. To identify these populations, the data were calibrated (Section 6.3) and the reagents in buffer control (Section 5.2) was performed. Figure 13(B) and (C) show the measured fluorescence intensity in standard units versus particle diameter of the reagents in buffer control and stained plasma sample, respectively, confirming the presence of antibody‐fluorophore aggregates. In addition, the brightest population is associated with platelets, because both the calibrated fluorescence intensity and size range are typical for platelets. Other possible causes of false positive fluorescence signals include binding of the Fc region of an antibody to Fc receptors present on EVs (Figure 11C) or sticking of antibodies to EVs or other particles (Figure 11E).

To confirm that fluorescent signals originate from EVs, assay controls (Section 5) are needed. The seven most common assay controls for fluorescence staining are listed in Figure 10. In short, to differentiate EVs from particles originating from the reagent, such as antibody‐fluorophore aggregates, buffer with reagents controls (Section 5.2) or procedural controls (Section 5.6) can be used. To determine the level of background noise and autofluorescence, unstained controls (Section 5.3) can be used. To identify antibodies that bind to Fc‐receptors or that are sticky, isotype controls can be used (Section 5.4). When multiple antibodies are used simultaneously, fluorescence‐minus‐one (FMO) and single‐stained controls (Section 5.5) can be used to investigate whether antibodies aggregate to each other. Detergent treatment (Section 5.8) can be used to verify that signals originate from membrane‐enclosed particles, such as EVs, and not from detergent‐resistant particles. Two controls that are not discussed in detail in this compendium, but which are recommended especially when using new antibodies to new samples, are the negative control and the pre‐blocking control. With the negative control, a sample containing EVs that do not have the targeted antigen is used to confirm the absence of a signal and thus non‐specific binding. With the pre‐blocking control, the targeted antigens of Es are pre‐blocked with antibodies that are not conjugated to a fluorophore. Samples with pre‐blocked antigens should not stain positively and can therefore be used as a negative control.

## 5 ASSAY CONTROLS

Assay controls are used to confirm that the measured events can be attributed to EVs. Assay controls are also important to identify sources of background noise and to optimize the settings of the flow cytometers, such as the trigger threshold(s). However, assay controls are not meant to apply quantitative corrections to data, such as the subtraction of background noise.

In this chapter, buffer‐only controls (Section 5.1), buffer with reagents controls (Section 5.2), unstained controls (Section 5.3), isotype controls (Section 5.4), FMO and single‐stained controls (Section 5.5), procedural controls (Section 5.6), serial dilution controls (Section 5.7), and detergent treatment controls (Section 5.8) will be discussed. Figure 10 shows to which experimental aspects each assay control applies to. Although many of these controls are also used in cellular cytometry, the procedural controls, serial dilution controls, and detergent treatment controls are specific to EV FCM. Each description starts with background information and a definition of the goal of the control, followed by a description of the procedure(s) covering problems and solutions specific to EV FCM.

### 5.1 Buffer‐only controls

The goal of the buffer‐only controls is to quantify the contribution of background noise (Section 2.3.3) and particles in the dilution buffer and compare it to the total counts of particles in the EV sample(s). In addition, the buffer‐only controls can be used to set the trigger threshold(s) and confirm whether the flow cytometer is clean. Buffer‐only controls should always be performed, reported, and considered in data interpretation.

#### 5.1.1 Procedure

As EVs are diluted in the buffer, the buffer requires careful selection. The buffer should have the same osmolarity and pH as the medium of the EV sample, be as free as possible of detectable particles and ideally sterile to avoid the presence of bacteria, fungi, and outer membrane vesicles. Buffers should be used freshly to reduce the presence of calcium‐phosphate precipitates (Larson et al., 2013). Filter‐sterilized buffers can be acquired commercially and techniques such as deionization, degasification, filtration, photolysis, and/or ultracentrifugation can be employed to reduce the presence of particles. When storage of buffers is required, it is recommended to freeze the buffer or store the buffer at 4°C for a maximum of 2 weeks.

Measure the dilution buffer using the same acquisition settings as the measurement of the EV sample(s). If the buffer‐only controls result in more events than desired, for example when the electronic abort rate is higher than desired (Section 2.3.3.4), the following steps can be taken to reduce unwanted background events.

Background events originating from particles in the dilution and sheath buffers can often be reduced by applying cleaning techniques or switching sheath filters (Section 2.3.1). Background events originating from electronic and optical noise might be minimized by modifications of the electronics and optics, respectively (de Rond et al., 2020). Nevertheless, hardware modifications require expertise and these components are usually not adjustable by the user, thus electronic and optical background noise often must be accepted as characteristics of the flow cytometer. If the dilution and sheath buffers are clean and the hardware is functioning optimally, background events may be further reduced by increasing the trigger threshold. Be aware that although increasing the trigger thresholds decreases the background events, it also decreases the sensitivity and therefore results in the detection of lower EV concentrations.

### 5.2 Buffer with reagents controls

Despite EV purification (section 3.2) or dilution, unbound reagents may contribute to background noise. The goal of the buffer with reagents controls is to quantify the contribution of unbound reagents to the total counts of particles in the EV sample(s). Buffer with reagents controls should be performed, reported, and considered in data interpretation whenever reagents are added to a sample.

#### 5.2.1 Procedure

Prepare a sample of dilution buffer under the same conditions as the EV sample(s), including all staining, washing, and dilution steps. Measure the buffer with reagents using the same settings as the measurement of the EV sample(s) and compare the counts to the buffer‐only controls (Section 5.1). Ideally, the buffer with reagents controls and buffer‐only controls have similar counts. If the buffer with reagents controls results in more counts than desired, steps can be taken to reduce counts originating from unbound reagents, such as titrating the reagents (Section 4.2.1.3) and/or reducing antibody‐fluorophore aggregates (Section 4.2.1.1).

Prior to staining, the concentration of particles originating from the reagent, such as antibody‐fluorophore aggregates, can be reduced by centrifugation or filtration (Section 4.2.1.1). Figure 13(B) and (C) show that antibody‐fluorophore aggregates can be recognized as events with a higher fluorescence signal to light scattering signal ratio than EVs. Whereas a single type of antibody may produce minimal background in buffer, the same antibody may result in aggregates when multiple different types of antibodies are simultaneously used to stain EVs.

Post staining, micelles formed by lipophilic dyes may be removed by density gradient centrifugation (Section 3.2.2). The contribution of other unbound reagents may be reduced by increasing the sample dilution or optimizing or changing the EV purification procedures (Section 3.2). Alternatively, counts attributed to unbound reagents can be reduced by increasing the trigger threshold at the expense of sensitivity.

### 5.3 Unstained controls

Due to the small size of EVs (Section 2.1.1), fluorescence levels of stained EVs are often indistinguishable from the background fluorescence level. In addition, staining reagents may affect the number of detected events, for example by inducing swarm detection. The goals of the unstained controls are to (1) determine at which fluorescence level the fluorescently stained EVs can be differentiated from unstained particles, (2) provide a reference for the number of events detected with and without reagents, (3) check for swarm detection induced by unbound reagents. Given the range of utility in assessing the quality of acquired data, unstained controls are recommended.

#### 5.3.1 Procedure

Prepare the unstained controls under the same conditions as the stained EV sample(s), including all washing and dilution steps, but omit the staining procedure. Measure the unstained controls using the same settings as the measurement of the stained EV sample(s).

The unstained controls provide an indication of the background fluorescence levels, which is helpful to determine the fluorescence gate that differentiates between stained particles and unstained particles. Stained samples may result in a higher number of events than unstained controls, because either aggregates in the reagents may contribute to false positive counts, which could be confirmed by the buffer with reagents controls (Section 5.2), or unbound reagents are causing swarm detection, which could be confirmed by an increased background fluorescence level of the stained samples compared to the unstained controls. When unbound reagents cause swarm detection, the concentration of unbound reagents should be decreased by dilution or EV isolation (Section 3.2). Unbound reagents could originate from the staining procedure, the cell culture medium (e.g., phenol red), the processed biofluid (e.g., anticoagulants), or non‐EV particles in the sample.

### 5.4 Isotype controls

During antibody staining, antibodies may bind by mechanisms other than the envisioned antibody‐antigen interaction, thereby leading to undesired false positive events and a decreased resolution between background fluorescence and true positive events. For example, Figure 11(C) shows that the Fc region of an antibody can bind to Fc receptors at the surface of EVs. In addition, Figure 11(E) shows that depending on their physicochemical properties, antibodies can also be sticky. The goal of isotype controls is to quantify the contribution of antibodies that bind to Fc receptors.

Isotype controls require isotype control antibodies, which are antibodies with the same Fc regions (e.g., IgG_1_ and IgG_2a_) as the antibodies used to stain EVs, but with a Fab region that recognizes irrelevant antigens, as shown in Figure 11(F). Hence, isotype controls provide insight into Fc receptor‐mediated binding.

Isotype controls should be performed when validating assays involving antibody staining, especially when assays involve EVs derived from cell types whose Fc receptor expression have not been characterized. Isotype controls are particularly important when staining EVs in blood plasma, because blood plasma contains several cell types expressing Fc receptors. Isotype control antibodies are not a perfect surrogate for evaluating the ‘stickiness’ of an antibody, because isotype control antibodies and antibodies differ.

#### 5.4.1 Procedure

Prepare the EV sample(s) under the same conditions as during antibody staining (Section 4.1.1), including all dilution and/or washing steps, but use an isotype control antibody instead of the original antibody to stain the EVs. Ideally, the antibody and isotype control antibody (1) are applied with the same concentration, (2) are conjugated with the same fluorophore, and (3) have the same F/P, although this is not often feasible to achieve in practice. Measure the isotype controls using the same acquisition settings as the measurement of the EV sample(s). If the isotype controls result in higher counts than desired, Fc receptors present on the EVs in a sample can be blocked by the addition of isotype antibodies without fluorophores.

Due to possible differences in concentration, fluorophore type, and F/P between antibodies and isotype control antibodies, it is not recommended to use isotype controls to define gates or to subtract positive counts or intensities of the isotype control from those of EV samples. If the contribution of Fc receptor binding is substantial, the use of an Fc blocking reagent is recommended.

### 5.5 Fluorescence‐minus‐one and single‐stained controls

When an EV sample is stained with a mixture of antibody fluorophore conjugates, it is important to assess whether one fluorophore affects the measurement of another fluorophore. For example, fluorophores with spectral overlap may cause spectral spillover, wherein a part of the signal of one type of fluorophore is picked up by the detector corresponding to another type of fluorophore. To compensate spectral spillover, antibody capture beads can be used to quantify the magnitude of spectral spillover and correct for it mathematically^18^. Compensation of dim signals originating from EVs, however, can result in spread of background fluorescence levels, making thresholds set on unstained controls inaccurate.

The goal of FMO controls is to determine the background fluorescence level in the absence of one fluorescent antibody conjugate, which is helpful to determine the fluorescence gate that differentiates between stained particles and unstained particles. The goals of single‐stained controls are to (1) validate the compensation of spectral spillover, and (2) identify potential confounding factors from fluorescent staining, such as quenching of one fluorophore by another fluorophore (Section 2.2.2.2).

When composing a mixture of antibodies to stain EVs, it is advisable to avoid using fluorophores that exhibit substantial spillover, especially because compensation of dim signals can result in a spread of background fluorescence levels. However, when using a mixture of antibodies with substantial spillover, FMO and single‐stained controls should be used.

#### 5.5.1 Procedure

For the FMO controls, prepare the EV sample(s) under the same conditions as during antibody staining (Section 4.1.1), including all dilution and/or washing steps, but leave out one type of fluorescent antibody conjugate from the mixture of staining reagents. Measure the FMO control using the same acquisition settings as the measurement of the EV sample(s) and do this for each type of fluorescent antibody conjugate in the mixture of staining reagents. For example, if EVs are stained with the antibodies A, B, and C, then the appropriate FMO controls would be A + B, A + C, and B + C. The measurement of A + B can be used to determine the background fluorescence level corresponding to measuring C.

For the single‐stained control, prepare the EV sample(s) under the same conditions as during antibody staining (Section 4.1.1) including all dilution and/or washing steps, but stain the samples with each type of fluorescent antibody conjugate separately. Measure the single‐stained control using the same acquisition settings as the measurement of the EV sample(s). When FMO and single‐stained controls give the same results regarding background fluorescence level and EV fluorescence intensities, spectral spillover is absent. When the single‐stained controls give the same results as EV samples(s) stained with the full mixture of staining reagents, quenching of one type of fluorophore by another type of fluorophore does not take place.

### 5.6 Procedural controls

To ensure the detection of single fluorescently stained EVs, assays may require removal of unbound reagents by EV purification methods (Section 3.2). However, some combinations of reagents and EV purification methods cause artifacts, such as false positive fluorescent events that resemble the signals of fluorescent EVs, hence leading to misinterpretation of results. Known artifacts are the formation of dye‐micelles or micelle‐like structures, aggregation of antibodies, and staining of non‐EV particles (Hoen et al., 2012; Morales‐Kastresana et al., 2019; Morales‐Kastresana et al., 2017; van der Vlist et al., 2012).

The goal of procedural controls is to identify potential artifacts due to the combination of certain reagents and EV purification methods. Procedural controls can be seen as an extension of other controls, such as the buffer‐only controls (Section 5.1) and buffer with reagents controls (Section 5.2) and can aid to develop optimized staining protocols. Procedural controls are recommended when unbound reagents are removed with an EV purification method after fluorescent staining.

#### 5.6.1 Procedure

The procedural control is usually combined with other assay controls, such as the buffer‐only control and the reagents in buffer control. For example, to investigate whether an EV purification method itself introduces detectable particles in the sample, for example when using a dirty filter or column, apply the EV purification method to buffer‐only under the same conditions as the stained samples(s). Measure the purified buffer samples using the same acquisition settings as the measurement of the EV sample(s). Ideally, this procedural control results in similar counts as the buffer‐only controls (Section 5.1).

To investigate whether an EV purification method introduces detectable fluorescent particles originating from the staining reagent, apply the EV purification method to reagents in buffer under the same conditions as the stained samples(s). Measure the purified reagents in buffer samples using the same acquisition settings as the measurement of the EV sample(s). Ideally, this procedural control results in similar counts as the buffer with reagents control (Section 5.2).

### 5.7 Serial dilution controls

At physiological EV concentrations, hundreds or more EVs and non‐EV particles can pass through the interrogation point simultaneously and may cause swarm detection (Section 2.3.3.4) (van der Pol et al., 2012). Swarm detection may go unnoticed, especially because a part of the EVs and non‐EV particles does not exceed the detection threshold. Therefore, extra controls are required to ensure single EV detection.

The simple solution to avoid swarm detection is sample dilution. However, by diluting the sample, the number of measured EVs as well as the statistical significance of the measurement decrease. In addition, different samples may require different dilutions. The goal of the serial dilution controls is to obtain the lowest sample dilution and thus the highest count rate without the occurrence of swarm detection. Ideally, serial dilutions are applied to every sample to confirm the absence of swarm detection.

#### 5.7.1 Procedure

Dilution series are preferably performed with stained EV samples, because the staining reagents may also affect or cause swarm detection. Prepare at least four dilutions to span 2–4 orders of magnitude and measure each dilution using the same acquisition settings as the optimally diluted stained EV samples. The dynamic range of the measured concentration of a flow cytometer typically exceeds 2 orders of magnitude, whereas the concentration of submicron‐sized particles in body fluids may also differ two orders of magnitudes between donors. Therefore, the range of serial dilutions should span 2–4 orders of magnitude. To find the optimal dilution, plot the measured concentration and median signal intensities versus dilution using logarithmic scales. Swarm detection is absent for the dilutions where the concentration scales linearly with the dilution and where the median fluorescence and light scattering intensities are independent of the dilution (Libregts et al., 2018; Stoner et al., 2016).

**FIGURE 14:**
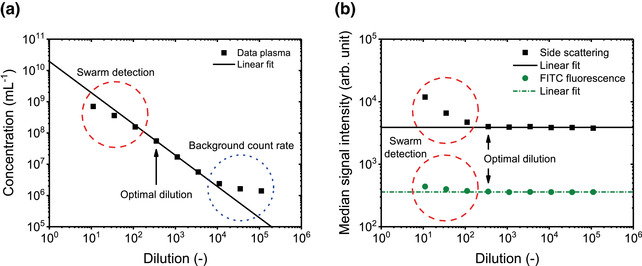
Serial dilution to determine the minimum required dilution to prevent swarm detection. (A) Measured concentration (Apogee A60‐Micro) and (B) measured median side scattering intensity and median fluorescein (FITC) fluorescence intensity in arbitrary units (arb. unit) of particles in pooled (5 healthy male and 5 healthy female donors) cell‐depleted plasma exceeding a side scattering cross section of 10 nm^2^ versus dilution. Data are fitted with a linear function. The dashed circles indicate dilutions for which swarm detection occurs. The dotted circle indicates dilutions for which the background counts dominate the measured concentration. The optimal dilution is the lowest dilution for which swarm detection does not occur (arrows).

For example, Figure 14(A) shows the measured concentration of particles in cell depleted plasma versus dilution. The dilutions span 4 orders of magnitude and increase in steps of $\sqrt{10}$ to accomplish equal spacing on a logarithmic scale. For dilutions >350‐fold, the measured concentration scales linearly with the dilution, except for dilutions resulting in a concentration corresponding to the background count rate. For dilutions <350‐fold, however, the concentration does not scale linearly with dilution because swarm detection occurs. To confirm the presence of swarm detection, Figure 14(B) shows that the median side scattering intensity and MFI of particles in the same plasma sample increase for dilutions <350‐fold. Therefore, the optimal dilution is 350‐fold.

The optimal dilution depends on many factors, such as the dimension of the interrogation point, which in turn depends on the flow rate, the concentration of reagents, the purity of the sample, and the concentration and size distribution of particles in the sample. Therefore, a universally applicable measurement procedure for EVs does not exist and the use of a universal dilution factor for the analysis of a given fluid type is not recommended.

Although serial dilutions are ideally applied to every sample, serial dilutions of all samples may be unfeasible in large studies. Instead, serial dilutions can be performed on a few representative samples to estimate the critical (measurable) concentration below which swarm detection is absent. To avoid swarm detection, other samples in the study should then be diluted sufficiently to be below this critical concentration. Most likely, the critical concentration depends on the flow cytometer and sample type. Research on the existence of a critical concentration below which swarm detection is absent and the validity of its use is still ongoing.

### 5.8 Detergent treatment controls

Biological samples often contain particles within the same size range as EVs. These particles may be mistaken for EVs, which leads to undesired false positive counts. The goal of detergent treatment controls is to differentiate detergent‐sensitive membrane‐enclosed particles, such as cells and EVs, from detergent‐resistant particles, such as detergent‐resistant membrane domains, immune complexes, and lipoprotein particles (Cloutier et al., 2013; György et al., 2011; Schroeder et al., 1994; Seddon et al., 2004), and thus estimate the contribution of particles misidentified as EVs.

Although there are well‐established detergent treatment protocols for lysing cells, the effect of detergent treatments on EVs and other particles with subcellular dimensions is not well‐known (György et al., 2011). For example, plasma contains EVs, but also may contain platelets and cell fragments (Arraud et al., 2014; Bettin et al., 2022), which all have a phospholipid membrane and will also lyse in the presence of a detergent. The lysis of particles therefore does not necessarily confirm the presence of EVs. Whether EVs or other subcellular particles lyse upon detergent treatment will also depend on the particle composition and the type of detergent used and therefore requires further investigation. Moreover, excess detergent may form micelles, which may cause detection artifacts. Systematic studies investigating detergent type, concentration, and incubation time across EV sources and other subcellular particles and their concentrations are required to ascertain whether a given detergent is suitable. It is recommended to apply the detergent treatment to a few representative samples of each assay.

#### 5.8.1 Procedure

Prepare a non‐ionic detergent at a concentration higher than the critical micelle concentration. Non‐ionic detergents have the ability to disrupt lipid‐lipid and lipid‐protein interactions and therefore can lyse membrane‐enclosed particles, such as EVs (György et al., 2011; György et al., 2012). In contrast to non‐ionic detergents, ionic detergents can also disrupt protein‐protein interactions and therefore can lyse particles that are held together by protein‐protein interactions, such as lipoprotein particles. Determine the optimal concentration of the detergent by titration. As a control, add the detergent to the dilution buffer to verify the absence of events associated with particles, such as micelles, originating from the detergent.

Prepare the EV sample(s), including all staining, washing, and dilution steps. Measure the EV sample(s) with and without addition of the detergent and verify whether fluorescence positive events disappear after addition of the detergent. The presence of fluorescence positive events after the addition of the detergent suggests that non‐EV particles have been stained and should therefore be reported.

## 6 INSTRUMENT DATA ACQUISITION AND CALIBRATION

### 6.1 Trigger detector and threshold

A flow cytometer continuously measures optical signals with multiple fluorescence and light scattering detectors. For most of the time and at an appropriate sample dilution (Section 5.7), however, no detectable particle is present in the interrogation point. To prevent storage of irrelevant data, only particles and background noise with signal levels exceeding the trigger threshold of one or more detectors are recorded (Section 2.3.3). Selection of the trigger detector and threshold are therefore essential because they define the level of background noise and which particles are detected.

#### 6.1.1 Selecting the trigger detector(s)

Whether to trigger on a fluorescence or scatter detector is a decision that should be made during the experimental design (Section 3.1). A fluorescence‐based trigger detects all particles that emit sufficient fluorescence to exceed the trigger‐threshold. To emit fluorescence, EVs are stained with fluorescent staining reagents (Section 4.1). In the pursuit of measuring all EVs, many fluorescence‐based trigger assays are based on generic fluorescent stains (Section 4.1.2). Although generic fluorescent stains may allow membrane specific detection, there is no generic fluorescent stain that stains all EVs (100% sensitivity) and only EVs (100% specificity), as other particles, such as lipoproteins, may also be stained (de Rond et al., 2019; de Rond et al., 2018). In addition, generic fluorescence stains may require EV purification steps to remove unbound particles, such as micelles, which may affect the measured EV concentrations (Hoen et al., 2012) and requires an additional procedural control (Section 5.6).

A scatter‐based trigger includes all particles, both EVs and non‐EV particles, which scatter enough light to exceed the trigger‐threshold. In contrast to traditional FCM of cells, where FSC provides a robust and reliable trigger signal, SSC is generally more suitable for EV detection than FSC (Section 2.3.2.3). Light scattering signals tend to be brighter than the fluorescence signals, because every atom scatters light. However, the background noise associated to stray light and particles in the sheath fluid is higher for scatter detectors than for fluorescence detectors because stray light cannot be spectrally filtered and all particles in the sheath fluid scatter light. Therefore, scatter detection typically results in a lower S/N than fluorescence detection. Whether fluorescence or light scatter triggering leads to detection of smaller and thus more EVs, however, depends on the flow cytometer, sample and fluorescent staining reagent used (de Rond et al., 2018).

#### 6.1.2 Selecting the trigger threshold

After selecting the trigger channel(s), the trigger threshold needs to be set for each trigger detector. Figure 8 illustrates the function of the trigger threshold. To select the optimal trigger threshold for a given assay and flow cytometer, it is important to understand how the background noise and trigger threshold affect which particles can be measured. For most flow cytometers, optical signals originating from EVs are dim and will partially overlap with the background noise. The signal level at which EVs can be distinguished from the background noise is the lower LoD of the used detector. Typically, a portion of the EV population in a sample does not exceed the lower LoD and can therefore not be detected. The few flow cytometers that do have the sensitivity to detect the smallest EVs, however, are constrained by an upper LoD, because larger EVs in the sample saturate the detector. The range between the upper and lower LoD, called the dynamic range (Section 2.3.3.5), is therefore insufficient to detect all EVs for current flow cytometers. From this perspective, an advantage of fluorescence detection is that it requires a lower dynamic range than light scatter detection, because the fluorescence signal of surface stained EVs scales with the 2^nd^ power of the diameter. For comparison, the scatter signal of the smallest EVs scale approximately to the 4^th^ power of the diameter, assuming that EVs are core‐shell particles (Section 2.2.3.4).

The trigger threshold can be set at or above the background noise level. When the trigger threshold is set at the background noise level, the electronics will include events attributed to background noise, which could be used as a reference. However, background noise is continuously present and therefore can, depending on the trigger threshold, trigger more background noise than actual particles. Consequently, a trigger threshold at the background noise level can be demanding on the processing speed of the electronics. On the other hand, a trigger threshold above the background noise level excludes events attributed to background noise and reduces the number of electronic aborts, but also excludes particles that otherwise could have been detected.

**FIGURE 15:**
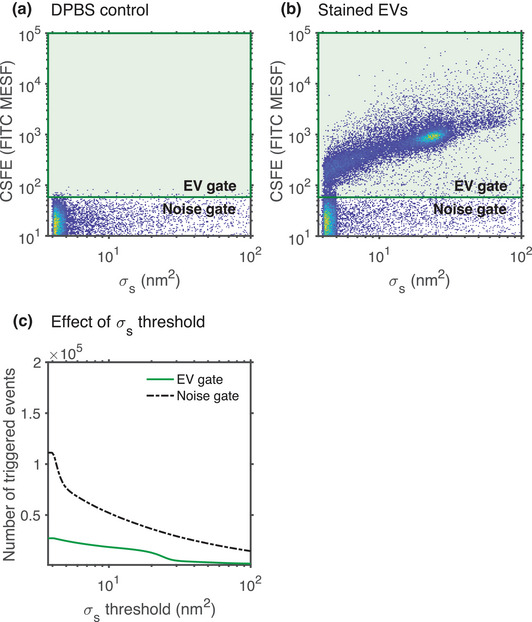
Effect of the trigger threshold on the number of measured EVs and on the number of events associated with background noise. Fluorescence intensity versus effective scattering cross section ($\sigma_{s}$) of (A) Dulbecco's phosphate‐buffered saline (DPBS) and (B) extracellular vesicles (EVs) from DC2.4 cell lines stained with carboxyfluorescein succinimidyl ester (CSFE). The fluorescence intensity of fluorescein (FITC) is calibrated into units of molecules of equivalent soluble fluorophore (MESF). The green line indicates the gate that differentiates fluorescently stained particles from fluorescent background noise. (C) Number of triggered events versus the threshold applied to the light scattering detector provided in the same units as the effective scattering cross section in panel A and B. For a decreasing threshold, the number of events associated with background noise increases more strongly than the number of measured EVs.

As an example, Figure 15 demonstrates how the trigger threshold can affect the number of measured EVs and the number of events associated with background noise. Figure 15(A) shows the fluorescence signal of CSFE in Dulbecco's PBS versus the effective scattering cross section. The horizontal line differentiates background noise from stained particles. Most particles fall below the horizontal line, which confirms that the reagents in buffer control is negative. Figure 15(B) shows the fluorescence signal of EVs stained with CSFE versus the effective scattering cross section. The fluorescently stained EVs exceed the horizontal line and therefore their detectability is limited by the light scatter threshold and not by the fluorescent sensitivity. Based on this measurement, Figure 15(C) shows the number of triggered noise events and EVs as a function of the trigger threshold, which is expressed as the effective scattering cross section. The lower the trigger threshold, the higher the number of measured EVs but also the higher the number of noise events. The sudden increase in events just below ∼5 nm^2^ revels the opto‐electronic noise floor. Reducing the triggering threshold below ∼5 nm^2^ would result in an event rate too large for the electronics to process and would result in undercounting EVs. To define the trigger threshold, procedures based on assay controls, such as the buffer‐only controls (Section 5.1) and the buffer with reagents controls (Section 5.2) for scatter‐based triggering, and the unstained controls (Section 5.3) or FMO controls (Section 5.5) for fluorescence‐based triggering, can help.

Given the complexity of this procedure, there is yet to be a consensus on the best method for selecting the trigger detector and threshold. To complicate matters, it is possible to set AND or OR gates on multiple detectors. Moreover, some flow cytometers have the option to determine the trigger threshold automatically and dynamically (Section 2.4.2). Consequently, it is unlikely that a consensus will be reached because the trigger threshold strategy depends on the assay and the type of flow cytometer used. However, given the importance of the trigger threshold, there is consensus in the EV‐field that the trigger detector should be calibrated (Section 6.3) and reported in standard units (Welsh et al., 2020).

### 6.2 Sample volume determination and flow rate stability

The concentration of EVs is a widely reported property. Flow cytometers determine the concentration of EVs by counting the number of measured EVs within the volume of sample fluid that has passed through the laser intercept during the acquisition. The concentration of EVs is particularly difficult to measure because flow cytometers are unable to detect all EVs (Section 8.1), but also because accurate determination of the sample volume is challenging. Methods to estimate the sample volume include (1) using a calibrated peristaltic or syringe‐based pump to inject the sample into the sheath flow, (2) measuring a known concentration of reference particles, (3) weighing a volume of water before and after analysis, and (4) using a flow rate sensor. The listed methods to measure the sample volume are all valid and may provide consistency when used on a single flow cytometer. However, these methods all have limitations and therefore may show different degrees of variability if used interchangeably between different flow cytometers.

#### 6.2.1 Calibrated pump

In the use of a calibrated peristaltic or syringe pump, it is assumed that the injected sample volume equals the adjusted sample volume. However, the piston, motor, and/or tubing of a peristaltic or syringe pump are subject to wear, thereby decreasing the accuracy of calibrations over time. Therefore, peristaltic and syringe pumps require periodic calibration using another method to determine the sample volume accurately.

#### 6.2.2 Counting beads

The accuracy of sample volume determination using reference particles with a known concentration (also called counting beads), is contingent on the accuracy of the reported concentration of the beads. Aggregation of these beads will result in an underestimation of the measured volume. Several steps can be taken to minimize the effects of bead aggregation. These include use of appropriate storage and dilution buffers, thorough vortexing and/or sonicating the beads before use, and by accounting for multiplets. A multiplet is an event where a few particles were coincidentally detected. Multiplets can often be distinguished from single particles by their proportionally higher fluorescence and light scattering signals. Ideal counting beads for EV applications would: (1) have a similar brightness as EVs, (2) not sediment, (3) be amenable for spiking into samples (i.e., plasma) without aggregating or affecting EVs, and (4) have concentrations that are determined in a metrologically traceable manner. Counting beads fulfilling the aforementioned criteria, however, are currently not commercially available.

#### 6.2.3 Determine sample volume weight

Using a reliable and accurate scale, a sample of water can be weighed pre‐ and post‐acquisition to deduce the sample volume analysed since water has a density of 1 g/ml at 25°C. Several assumptions are made when using this method, including (1) the measuring scale is accurate, (2) there is a negligible dead or void volume, which is the total sample volume from the sample injection tube to the interrogation point, (3) no backflush of sheath fluid from the sample injection tube into the sample tube upon finishing the acquisition, and (4) the absence of residual sample at the sample injection tube. To ensure a negligible void volume, a sample with water can be measured for a longer time than the measurement time.

#### 6.2.4 Flow rate sensor

**FIGURE 16:**
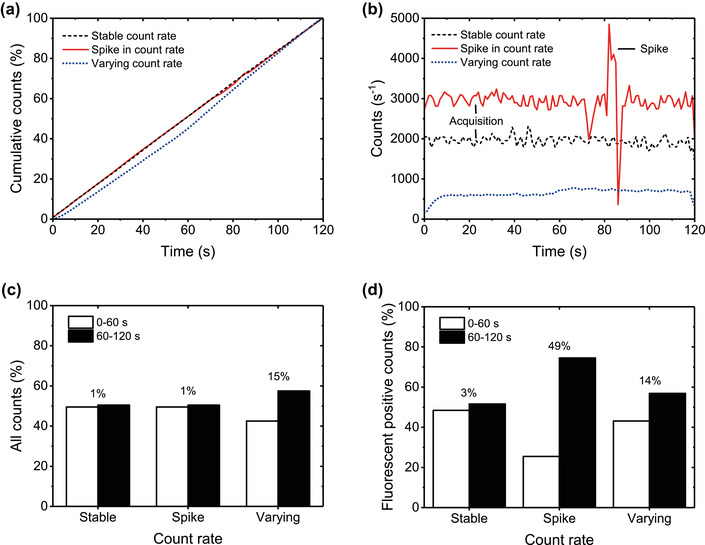
Relevance of checking the count rate. (A) Cumulative distribution function of the counts and (B) histogram of the counts versus time for extracellular vesicle (EV) samples measured with a stable count rate, a spike in the count rate, and a varying count rate. The samples with a stable count rate and a spike in the count rate contain particles in cell‐depleted plasma stained with CD45‐APC measured with an Apogee A60‐Micro. The sample with a varying count rate contains particles form a cell‐depleted erythrocyte concentrate stained with CD235a‐PE measured with an FACSCanto II (BD, USA). The cumulative distribution function of the counts clearly reveals flow rate variations that last long relative to the measurement time, whereas the histogram of the counts versus time makes spikes visible. For the A60‐Micro measurements, count rate fluctuations within 400 s^−1^ are caused by the data acquisition. (C) Fraction of the total counts and (D) CD45‐APC+ or CD235a‐PE+ EVs measured during the first and last 60 s in the same samples as measured in panels A and B. Differences in counts between the first and last 60 s of the measurement were negligible (1%–3%) for the sample with a stable count rate, negligible (1%) for the total counts of the sample with spikes in the count rate, 49% for the fluorescent positive counts of the sample with spikes in the count rate, and 14–15% for the sample with a varying count rate. Thus, spikes and variations in the count rate may lead to unreliable counts and concentration estimates.

The volume measurement methods as described above are at best an estimate of the sample volume due to the many assumptions taken in its determination. One assumption that applies to all periodically applied procedures is that the flow rate is stable. Without a flow rate sensor, the count rate provides an indirect way to monitor the flow rate stability. Figure 16 show the cumulative distribution function of the counts and a histogram of the counts versus time, respectively, for samples measured with a stable count rate, a spike in the count rate, and a varying count rate. The cumulative distribution function of the counts clearly reveals flow rate variations over a significant range of the measurement time, whereas in the histogram representation of the counts versus time this effect is obscured. Figure 16 further shows that during the first seconds of the measurement with a varying count rate, the flow rate increases, which is likely due to an unstable flow rate after the sample boost is engaged. To minimize the sample loading time, flow cytometers ‘boost’ the sample from the sample injection tube to the flow cell before data acquisition starts. When data acquisition starts too soon after the boost, the flow rate may still be unstable, which results in increased background noise and variation in data. Precautions that can be taken to reduce the effect of the boost on the stability of the flow rate are to incorporate a time delay (usually <1 min) before recording the data or acquiring data for an extended period of time and gating out the start of the measurement. Automated gating algorithms exist for removing flow rate inconsistencies in data (Meskas et al., 2020; Monaco et al., 2016).

To illustrate how spikes and variations in the count rate can affect the total counts, Figure 14 show the fraction of total counts and fluorescently stained EVs measured during the first and last 60 s of a 120 s measurement. Differences in counts between the first and last 60 s of the measurement were negligible (1%–3%) for the sample with a stable event rate, negligible (1%) for the total counts of the sample with spikes in the count rate, 49% for the fluorescent positive counts of the sample with spikes in the event rate, and 14–15% for the sample with a varying event rate. Thus, spikes and variations in the event rate may lead to unreliable counts and thus unreliable EV concentration measurements. Hence, it is important to check the stability of the event rate for each sample measured by plotting the cumulative distribution function of the counts and a histogram of the counts versus time.

### 6.3 Calibration

To enable comparison of measurement results across assays, flow cytometers and laboratories, a critical need for standardized measuring and reporting exists within the EV field. This need has arisen because the use of different experimental design and flow cytometers result in different LoDs, which leads to the measurement of different EV concentrations even within the same sample (Section 2.1.1).

The key to achieve standardization is calibration. In the metrological context, calibration is the conversion of an arbitrary unit scale to a scale of standard units with a known uncertainty. In contrast to normalization or quality controls, which keep the data in arbitrary units, calibration results in standard units that are independent of the flow cytometer and therefore allows comparison of data between different flow cytometers.

Aside from data comparison, calibration improves data interpretation. Figure 13(A) shows the measured fluorescence intensity versus the side scattering intensity of particles in plasma stained with CD61‐APC. The arbitrary units preclude identification of the three positively stained populations. For comparison, Figure 13(C) shows the measured fluorescence intensity in standard units versus the diameter of particles in the same sample. Owing to the calibration the three populations can be readily identified and the concentration of CD61+ EVs can be determined.

The next sections show an example of fluorescence and light scattering calibrations and discuss the procedures and limitations involved.

#### 6.3.1 Fluorescence calibration

**FIGURE 17:**
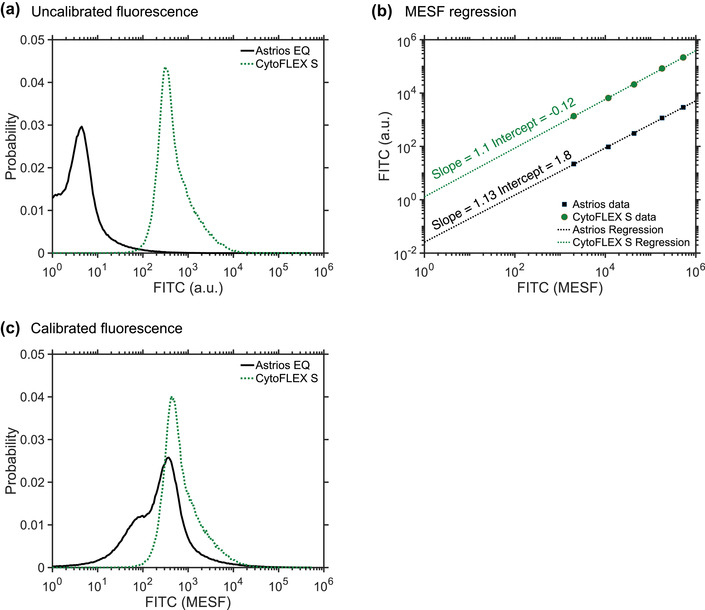
Relevance of fluorescence calibration. (A) Probability density function of extracellular vesicles (EVs) stained with carboxyfluorescein succinimidyl ester (CFSE) measured by the fluorescein (FITC) detector of a Beckman Coulter Astrios EQ (solid line) and a Beckman Coulter CytoFLEX S (dotted line). The peak intensities are at different locations on the arbitrary units (arb. unit) scale. (B) Calibration of the FITC detectors of a Beckman Coulter Astrios EQ (solid line) and a Beckman Coulter CytoFLEX S (dotted line). The measured arb. units are related to the specified molecules of equivalent soluble fluorochrome (MESF) of reference materials using linear regression. (C) Application of the MESF calibration from panel B to the data from panel A. The calibration results in comparable CFSE‐stained EV data.

Flow cytometers report fluorescence intensities in arbitrary units. For example, Figure 17(A) shows the fluorescence histogram of EVs stained with the generic dye carboxyfluorescein succinimidyl ester (CFSE) measured by a Beckman Coulter Astrios EQ and a Beckman Coulter CytoFLEX S (Morales‐Kastresana et al., 2019; Morales‐Kastresana et al., 2017). The trigger was set at the side scattering detector. Both flow cytometers measured the same EV sample, but the data are different, demonstrating that the arbitrary units hamper data interpretation and comparison. In addition, the sensitivity and dynamic range of the fluorescence detector cannot be compared and may be different^19^. Consequently, these flow cytometers may have detected different EVs.

To reveal the aforementioned issues, Figure 17(B) shows how the arbitrary units of fluorescence are related to the standard unit of MESF. The details of this calibration procedure are discussed in Section 6.3.1.1. By applying the calibration in Figure 17(B), Figure 17(C) shows the same fluorescence histograms of the CFSE‐stained EV population as Figure 17(A), but now in units of MESF. It becomes immediately clear that the two distributions of the fluorescence intensity, of which the peaks differed two orders of magnitude in Figure 17(A), do overlap after calibration. Thus, calibration leads to improved data interpretation and comparison. Fluorescence calibration should therefore be seen as a priority to improve the quality and reproducibility of EV FCM research (Welsh et al., 2020).

##### 6.3.1.1 Procedures

In FCM, there are currently two standard units for fluorescence intensity that can be applied to calibration of EV measurements: ABC and MESF^20^. MESF is the most frequently used unit and requires reference particles with an assigned fluorescence intensity (Gaigalas et al., 2005; Schwartz et al., 2004). MESF reference particles, also called MESF beads, have a surface that is stained with the fluorophore of interest. To assign the fluorescence intensity in units of MESF to the beads, the bead manufacturer compares a bulk fluorescence measurement of a known concentration of beads to a bulk fluorescence measurement of a known concentration of soluble fluorophores. For example, a bead with 1000 MESF APC has a brightness equal to 1000 soluble APC molecules. The brightness is expressed in MESF because the concentration and fluorescence intensity of soluble fluorophores can be readily determined, whereas the actual number of fluorophores of a bead cannot. MESF beads are generally spectrally matched to the same fluorophores conjugated to antibodies. Therefore, MESF beads can be used to calibrate flow cytometers with different excitation and detection characteristics (Sections 2.3.2.2 and 2.3.2.3). MESF beads for commonly used fluorophores, such as APC, FITC and PE, are commercially available.

To calibrate EV fluorescence intensity, the first step is to measure the MESF beads and determine the MFI per population at the same settings used to acquire the EV samples. Next, the data and the specified MESF values are logarithmically transformed and fitted by a linear regression to relate arbitrary units to standard units (Corso et al., 2019; Shen et al., 2018; Tan et al., 2004; Weller, 2018; Wiklander et al., 2018). To explain this procedure, Figure 17(B) shows the assigned MESF values of five bead populations versus their acquired median fluorescent intensity for the same flow cytometers as in Figure 17(A). Due to the linearity of FCM detectors, the measured fluorescence intensity scales linearly with the specified MESF values of each bead population. A log transformation is required to avoid that the regression is biased towards the relatively bright beads before a linear regression can be applied. After log transformation of both the data and the MESF values, the slope *a* and intercept *b* of the linear regression relate the arbitrary units of fluorescence $P_{F}\left[\mathrm{arb}.\mathrm{unit}\right]$ to the assigned number of fluorophores $P_{F}\left[\mathrm{MESF}\right]$ as follows:

(9) $$P_{F}\;\left[\mathrm{MESF}\right]\;=10^{\frac{\log\left(P_{F}\left[\mathrm{arb}.\;\mathrm{Unit}\right]\right)-b}{a}}$$

where log represents a logarithm with base 10. Please note that Equation (9) only applies when $P_{F}\left[\mathrm{arb}.\;\mathrm{unit}\right]$ is plotted versus $P_{F}\left[\mathrm{MESF}\right]$, such as in Figure 17(B). The linear regression can be calculated with a spreadsheet or using third‐party analysis software, such as FlowJo, FCSExpress, and FCM_PASS_ (Welsh et al., 2020).

When MESF beads are unavailable, for example in the case of a non‐common fluorophore, ABC beads can be used. ABC beads bear calibrated numbers of immunoglobulin binding molecules that can be stained with an antibody‐fluorophore of interest and used to calibrate the fluorescence intensity in units of ABC. The procedure for ABC calibration can be performed in analogy to the aforementioned procedure for MESF calibration. ABC calibration is independent of the F/P and the emission spectrum of the fluorophores. Similar to MESF beads, ABC beads are commercially available.

##### 6.3.1.2 Limitations

A limitation of MESF beads is that the interpretation of results requires careful interpretation for three reasons. First, when a soluble fluorophore is conjugated to an antibody and/or binds to a bead or an EV, the chemical environment of the fluorophore changes and therefore the fluorescence intensity and spectrum of the fluorophore change (Section 2.2.2.1). Consequently, units of MESF do not represent the actual number of fluorophores on a reference particle or an EV. Second, EVs emit autofluorescence, which may overlap in intensity and spectrum with the sparsely stained EVs. Third, multiple fluorophores may be bound to a single antibody. To relate units of MESF to the actual number of fluorophores on an EV, the change in fluorescence intensity and spectrum of a soluble fluorophore, when conjugated to an antibody and/or when bound to a bead or an EV, need to be determined with specialized equipment. In addition, the autofluorescence of an EV needs to be determined and the F/P needs to be known.

MESF beads were developed for cellular analysis. Therefore, MESF beads typically are polystyrene beads of several micrometres in diameter and have a minimum fluorescence intensity of thousands of MESF, which is bigger and brighter than EVs. Even the dimmest beads in a commercial kit may be brighter than the fluorescence of stained EVs. Consequently, to calibrate fluorescence intensities in the range of EVs requires data extrapolation, which adds to the uncertainty of the calibration. Moreover, the assigned MESF values may differ between manufacturers, which emphasizes the need to report all experimental details (Welsh et al., 2020). The limited shelf life of MESF beads requires periodical recalibration and purchasing.

A limitation of ABC beads is that antibody capture is specific for one species and thus requires distinct beads for IgGs from different species. Another limitation is that, depending on the capture antigen, different isotypes and clones may exhibit different binding properties, resulting in an inaccurate calibration. Like MESF beads, ABC beads have a limited shelf life and therefore require periodic recalibration and purchasing.

##### 6.3.1.3 Cross calibration using hard‐dyed beads

A daily calibration with ABC or MESF beads may be desired, but would be laborious, costly, and may lead to stability issues. An alternative approach is the use of hard‐dyed, multi‐peak, multi‐fluorophore beads, often referred to as “Rainbow” beads. Rainbow beads are typically polystyrene beads impregnated with multiple fluorophores that excite and emit across the full spectral range used in FCM. Rainbow beads can be cross calibrated against ABC and MESF beads to assign calibrated fluorescence intensity values to each population, which makes them useful as stable reference particles for daily instrument calibration and quality control. These cross‐calibrated intensity assignments are specific to the excitation and emission characteristics of a flow cytometer because the fluorophores within the rainbow beads are not spectrally matched to the fluorophores used for EV staining. For this reason, while ABC and MESF calibrations can be compared across flow cytometers, rainbow beads need to be cross calibrated on individual flow cytometers for optimal accuracy.

#### 6.3.2 Light scatter calibration

Similar to fluorescence intensities, flow cytometers measure light scattering intensities in arbitrary units, which makes data interpretation and comparison not only difficult, but also prone to misinterpretation. Misinterpretation particularly occurs when standardization of scatter signals is done by polystyrene or silica beads without the use Mie theory (Section 2.2.3.4). As explained in Section 2.2.3, the light scattering intensity measured by a flow cytometer depends on the particle diameter and refractive index as well as the optical configuration of the flow cytometer. As polystyrene beads, silica beads and EVs differ in refractive index and because flow cytometers differ in optical configuration, gates defined by the scattering intensities of beads select different sizes of EVs on different flow cytometers.

**FIGURE 18:**
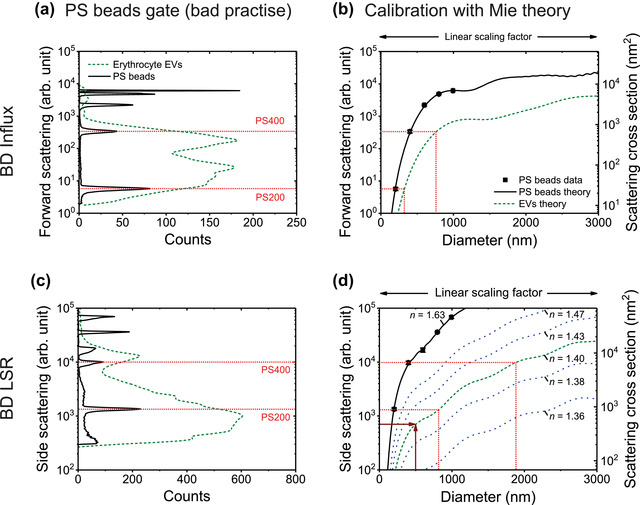
Relevance of scatter calibration. (A) Light scattering intensities of 200 and 400 nm polystyrene (PS) beads (solid line) and EVs stained with Glycophorin A (CD235a) from a cell‐depleted erythrocyte blood bank concentrate (dashed line) measured by the forward scattered light detector of a BD Influx. (B) Measured (symbols) and calculated (lines) light scattering intensity of PS (solid line) and EVs (dashed line) versus diameter for the BD Influx. The calibration factor, which relates the measured arbitrary units (arb. unit; left axis) to the theoretical scattering cross section (right axis), is 0.16. The calibration reveals that the 200 and 400 nm PS bead gate selects EVs with a diameter between ∼300 and ∼800 nm. (C) Idem as panel A, but measured with the side scattering detector of a BD LSR. (D) Idem as panel B, but for the BD LSR. The calibration factor is 1.47. The calibration reveals that the 200 nm and 400 nm PS bead gate selects EVs with a diameter between ∼800 nm and ∼1900 nm. Thus, a PS bead gate selects different EVs at different flow cytometers. EVs were modelled as solid spheres with a refractive index (*n*) of 1.40.

For example, Figure 18(A) and (C) show the light scattering intensities of 200 nm and 400 nm polystyrene beads and EVs stained with anti‐Glycophorin A (CD235a) from a cell‐depleted erythrocyte blood bank concentrate measured by a BD Influx and a BD LSR. From the histograms of the scattering intensities of the EVs it becomes clear that the bead gates select different EVs at different flow cytometers. To explain this, Figure 18(B) and (D) show the light scattering calibrations for these flow cytometers. Using Mie theory, the measured light scattering intensities of polystyrene beads are related to their effective scattering cross section and diameter, taking into account both the refractive index of polystyrene and the optical configuration of the flow cytometer. This calibration allows to predict the scatter versus diameter relation for EVs assuming a refractive index of 1.40. Due to the calibration, it becomes evident that within the 200 nm and 400 nm polystyrene bead gate, the BD Influx measured EVs with a diameter ranging from ∼300 to ∼800 nm, whereas the BD LSR measured EVs between ∼800 and ∼1900 nm. Figure 18(D) further shows that also silica beads, which range in refractive index between 1.43 and 1.47, still scatter more light than similar‐sized EVs. In contrast to what some manufacturers claim, silica beads should therefore not be used to set EV gates.

The example in Figure 18 shows the importance of calibration and understanding how the scatter to diameter relation of a flow cytometer depends on the particle refractive index. Due to calibration, it becomes clear that gates based on polystyrene or silica beads lead to data misinterpretation and incomparable results. Moreover, stating the minimum detectable diameter as a metric for the sensitivity of a scatter detector, which is common practice among manufacturers to advertise their instrument, provides no basis for comparison without stating the refractive index of the particle. For example, flow cytometers are regularly advertised as being “able to detect 100 nm particles”, which may suggest that the flow cytometer can detect EVs with a diameter of 100 nm. Typically, however, the 100 nm particles refer to 100 nm polystyrene beads, which have similar SSC (NA = 1.2, λ = 405 nm) signals as 25 nm silver beads, 49 nm gold beads, 136 nm silica beads, and 207 nm EVs. Supplemental materials 1 contains a video to provide insight into the scatter to diameter relation of EVs and beads. The video shows that the scatter to diameter relation does not only depend on the particle refractive index, but also on the collection angles of the flow cytometer.

In summary, the use of Mie theory to calibrate light scattering signals requires the knowledge of the optical configuration of the flow cytometer as well as refractive index of EVs (de Rond et al., 2018; van der Pol et al., 2012; Welsh et al., 2020). Calibrated light scattering signals allow comparing concentrations of EVs within the same size range, thereby reducing the variation in the measured concentration of EVs on different flow cytometers (van der Pol et al., 2018; Welsh et al., 2020).

##### 6.3.2.1 Procedure

Light scatter calibration relies on relating the median scattering intensities in arbitrary units of well‐characterized, homogeneous, non‐fluorescent beads, such as polystyrene and silica beads, to their theoretical scattering cross sections in units of nm^2^. The scattering cross sections are calculated with Mie theory (Section 2.2.3.4) to take into account the optical configuration of the flow cytometer and the refractive index contrast between the particle and the medium, and are typically expressed as the effective scattering cross section in nm^2^ (de Rond et al., 2018; van der Pol et al., 2012). The median scattering intensities of the beads can be related to their theoretical counterparts by a linear scaling factor, which can be obtained by least square fitting. Figure 18(B) and (D) show how the measured scattering intensity in arbitrary units is related to the theoretical effective scattering cross section in nm^2^ by a linear scaling factor. Best practice is to plot the scattering intensity versus particle diameter to confirm the calibration and instrument sensitivity, because the scattering intensities differ per type of flow cytometer. The scatter to diameter relation for EVs can be calculated, assuming a refractive index of EVs, and used to measure the size distribution of EVs (Section 7.1).

There are four options for implementing light scatter calibration (de Rond et al., 2018). First, measuring your own beads and performing your own optical modelling. This method requires understanding of the physics of single particle light scattering and mathematics, as well as knowledge of the optical configuration of the used flow cytometer (de Rond et al., 2018). Second, measuring your own beads and using free Mie calculation software. This method requires less understanding of physics and mathematics, but does still require a profound understanding of the optical configuration of the used flow cytometer. Third, measuring your own beads and using free semi‐automated calibration software (e.g., FCM_PASS_) (Welsh et al., 2020). This method only requires determining the median scatter intensities of your own beads, as physics, mathematical and flow cytometer knowledge are built into the software. Fourth, purchase a kit, which includes beads and automated calibration software (e.g., Rosetta Calibration, Exometry, Netherlands) (van der Pol et al., 2018). This method requires no knowledge of the flow cytometer, physics and math and simply involves importing of the data acquired from the beads in the kit into the software.

##### 6.3.2.2 Limitations

Although introduced to the EV‐field in 2012 (van der Pol et al., 2012), light scatter calibration is still seldomly utilized, likely due to complexity, the lack of educational resources, and the limited awareness of commercial and freely available software and materials to improve the ergonomic implementation of light scatter calibration. While light scatter calibration converts arbitrary units to standard units, such as the effective scattering cross section in nm^2^, these standard units still depend upon the collection angles and illumination wavelength of the flow cytometer. For example, Figure 18(B) and (D) show that the effective scattering cross sections of a 200 nm polystyrene bead differ due to the differences in the collection angles between the Influx and LSR. Hence, the effective scattering cross section can only be used to compare data between flow cytometers with the same collection angles and illumination wavelength.

To make scatter data comparable between flow cytometers with different optical configurations (van der Pol et al., 2018), light scattering intensities need to be related to the particle diameter. The accuracy of the estimated diameter by scatter calibration depends on the accuracy with which the refractive index of the particle is known. As the refractive index of polystyrene is well‐characterized, sizing polystyrene beads resulted in a measurement error <5% and a coefficient of variation of the particle size distribution width of <4%, which is more accurate and precise than NTA (van der Pol et al., 2014). For EVs, however, the refractive indices are not so precisely known. Measurements show that the mode of refractive index distributions of EVs from plasma and urine is at or below 1.40 (de Rond et al., 2019; Gardiner et al., 2014; Geeurickx et al., 2019; Konokhova et al., 2012; van der Pol et al., 2018; van der Pol et al., 2014), which is substantially lower than the refractive index of silica. As the refractive index of EVs will differ per source, the refractive index requires careful selection. In fact, the refractive index itself is not homogeneously distributed within an EV (Section 2.1.1), because based on refractive index measurements of cells, it is to be expected that the phospholipid membrane has a higher refractive index (1.40–1.52) than the lumen (1.34–1.42) (Brunsting & Mullaney, 1974; Curl et al., 2005; Ducharme et al., 1990; Ghosh et al., 2006; Horvath et al., 2003; Kienle et al., 2014; Maltsev et al., 2011; Valkenburg & Woldringh, 1984; van Manen et al., 2008). Mie theory, however, can take the refractive index distribution within EVs into account by modelling a core‐shell structure. An uncertainty evaluation is required to investigate how the assumed refractive index affects the accuracy of the estimated diameter for a given flow cytometer. Another assumption of Mie theory and EV diameter estimation in general is that EVs are spherical, as will be further discussed in Section 7.1.2.

A disadvantage of sizing by a single light scattering detector is that the scatter to diameter relationship does not always provide a unique solution for the diameter. This particularly applies to cytometers with low (<1.0) numerical aperture collection lenses. For example, Figure 18(D) shows that for the Influx, EVs between 1100 and 1500 nm generate the same FSC signal and can therefore not be differentiated by FSC.

## 7 EXTRACELLULAR VESICLE CHARACTERIZATION

### 7.1 Diameter, surface area and volume approximation of extracellular vesicles

The diameter is a frequently measured property of EVs, given the range of size distributions reported in literature. For FCM, EV diameter estimates improve the verification that the measured particles are EVs, which is particularly important because frequently used gates based on polystyrene beads may lead to the detection of larger particles than the envisioned EVs (Section 6.3.2) (van der Pol et al., 2012). Other applications of EV diameter estimates involve reducing interlaboratory variability by reporting EV concentrations within the same size range (van der Pol et al., 2018), comparing size distributions to other instruments (van der Pol et al., 2014), and using the size distribution to optimize EV purification methods. Once the EV diameter is estimated, it can be used to calculate the surface area and volume of an EV, which may lead to new biological and clinical insights (van der Pol et al., 2013). The standard units obtained by calibration not only facilitate data comparison, but also enable reporting of more insightful properties of EVs, such as the diameter, refractive index, as well as the total number and density of receptors.

#### 7.1.1 Procedures

There are multiple methods to estimate the diameter and size distribution of EVs by FCM. Using light scattering, the EV diameter can be determined by modelling and calibrating one light scatter detector with an assumed refractive index for the EVs of interest. This approach has now become accessible with free and commercially available software (van der Pol et al., 2018; Welsh et al., 2020), and has been shown to be more accurate and precise than sizing particles by NTA (van der Pol et al., 2014).

**FIGURE 19:**
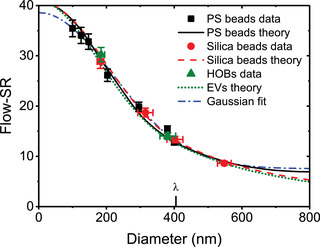
The flow cytometry scatter ratio (Flow‐SR), which is the ratio of the side to forward scattered light intensity, allows determination of the diameter of particles independent of the particle refractive index. Flow‐SR versus diameter for polystyrene (PS) beads (squares), silica beads (circles), and hollow silica beads (HOBs; triangles) measured (symbols) by an Apogee A60‐Micro operating at 405 nm wavelength (λ), calculated with Mie theory (lines), and fitted empirically with a Gaussian function (dotted line). For this flow cytometer, Flow‐SR provides a unique solution for the particle diameter between 0 nm and 600 nm.

To determine the EV diameter independent of an assumed refractive index, the FCM scatter ratio (Flow‐SR), which is the ratio between SSC and FSC, can be used. Figure 19 shows the Flow‐SR versus diameter for polystyrene beads, silica beads, and hollow organosilica beads (HOBs). Mie theory calculations confirm that for this flow cytometer, the Flow‐SR provides a unique solution for particle diameters between 0 and 600 nm. The use of Mie theory, however, is not required to derive diameter when using Flow‐SR. Flow‐SR can be implemented using beads with a diameter smaller than or equal to the illumination wavelength and then be fitted, for example with a Gaussian function.

As an alternative to Flow‐SR, custom‐built flow cytometers that allow for measuring angle‐resolved light‐scattering profiles per particle can be used to derive the particle diameter and refractive index (Konokhova et al., 2012; Konokhova et al., 2016; Konokhova et al., 2016). Here, Mie theory and optimization algorithms are used to find the best diameter and refractive index describing the measured angle‐resolved light‐scattering profile. Particles for which no accurate theoretical description of the angle‐resolved light‐scattering profile is present, are assumed to be non‐spherical and can be omitted from the analysis.

Along with light scattering, fluorescence can also be used to approximate the EV diameter using vesicle standards with well‐characterized size distributions (Stoner et al., 2016). When EVs are stained uniformly with a membrane dye, the fluorescence distribution of the vesicle standards is proportional to the surface area distribution of the EVs (Stoner et al., 2016). Hence, the vesicle standard can be used to relate fluorescence signals to the surface area and diameter of EVs that are stained with the same membrane dye under the same experimental conditions.

#### 7.1.2 Limitations

Like other sizing techniques, such as NTA and RPS, the measurement of EV size by FCM generally relies on the assumption that EVs are spherical. According to cryo‐EM, EVs <600 nm in human blood plasma are spherical (Arraud et al., 2014), whereas EVs >600 nm also include tubular EVs and membrane fragments, which represent 5% and 0.5% of all EVs, respectively. Approaches based on light scattering measurements and Mie theory to estimate EV diameter by FCM do not work for tubular EVs and membrane fragments, and thus will provide meaningless numbers. Only multiangle light scattering measurements can identify non‐spherical EVs and therefore are able to exclude them from the size distribution (Konokhova et al., 2012; Konokhova et al., 2016; Konokhova et al., 2016).

Sizing by a single scatter detector depends on the assumed effective refractive index or refractive index distribution of EVs, which is not precisely known and may differ between samples and EV types (Section 2.2.3.1). Therefore, size distributions of EVs obtained by FCM should be confirmed using orthogonal methods.

Sizing by Flow‐SR is limited by the lower LoD of the least sensitive scatter detector. For flow cytometers where the FSC detector is equipped with a photodiode, the FSC detector will be incapable of detecting EVs <1000 nm in diameter. For instruments with an APD or PMT detector to measure FSC, such as the Apogee A60‐Micro, BC Astrios, and BD Influx, the applicable size range of Flow‐SR is in between approximately 200 nm and 600 nm for EVs (de Rond et al., 2019; van der Pol et al., 2018). Particles larger than the applicable range are sized incorrectly and therefore are preferably removed from the sample prior to analysis.

Sizing EVs by angle‐resolved light scattering requires a custom‐built flow cytometer and custom‐developed software. World‐wide, only a few flow cytometers capable of measuring angle‐resolved light scattering of single particles exist. Maltsev et al. performed the first angle‐resolved light scattering measurements on submicron particles in human blood plasma samples with a custom‐built flow cytometer (Konokhova et al., 2012). With their instrument, they could differentiate EVs, LPs, and platelets without labelling and describe the relation between the background noise levels of FSC and SSC detectors and the size and refractive index of submicrometre particles (Konokhova et al., 2016; Konokhova et al., 2016). With 600 μs, however, the acquisition time per particle of their instrument is long compared to commercial flow cytometers, which have a typical acquisition time of 1 μs per particle. Moreover, the lower LoD of their instrument corresponds to the detection of 400 nm EVs, which is substantially less sensitive than commercial flow cytometers. Thus, the acquisition of more detailed information on submicron particles comes at the expense of speed and sensitivity.

Sizing EVs by fluorescence assumes that the brightness and surface density of the dyes that are used to stain membranes is equal for vesicle standards and EVs. However, the brightness depends on the chemical environment, which may differ between vesicle standards and EVs. In addition, differences in membrane composition between the vesicle standards and EVs may result in staining differences and thus inaccurate sizing. Finally, the size of vesicle standards is difficult to determine accurately because orthogonal techniques, such as cryo‐EM, NTA, and RPS, have their own limitations.

In summary, fluorescence and light scattering signals of flow cytometers can be used to measure the size and size distribution of EVs exceeding the lower LoD. This information increases our knowledge on the fundamental properties of EV samples, and in addition paves the way to standardized assays and clinical and pharmacological insights regarding the role of EVs as a biomarker and therapeutic target. All FCM‐based size estimates, however, have uncertainties that are the subject of current research.

### 7.2 Refractive index approximation of extracellular vesicles

Refractive index approximation of EVs is a relatively new, technologically challenging, yet important research area, because knowledge of the refractive index of EVs is essential for interpretation of light scattering signals from EVs and applications thereof, including data comparison and EV identification. For example, refractive index approximation can be used for label‐free differentiation between EVs (n<1.40) and LPs (n>1.45) (de Rond et al., 2019; Konokhova et al., 2016; van der Pol et al., 2018).

Although the refractive index of EVs is distributed concentrically (Section 2.1.1), hitherto only the effective refractive index of EVs has been measured with FCM and NTA (Gardiner et al., 2014; Konokhova et al., 2012; Konokhova et al., 2016; Konokhova et al., 2016; van der Pol et al., 2018; van der Pol et al., 2014). In contrast to FCM, commercial NTA instruments fail to differentiate EVs from LPs based on refractive index approximation (Gardiner et al., 2014; Geeurickx et al., 2019), likely due to a low precision in measuring size and light scattering intensity (de Rond et al., 2018; Khamsi, 2020).

This section briefly discusses two FCM‐based methods to approximate the effective refractive index of EVs.

#### 7.2.1 Procedures

The first approach to approximate the effective refractive index of EVs is explained by Figure 18(D) and involves calibration of a scatter detector using reference particles and Mie theory (Section 6.3.2), followed by an extrapolation of the scatter to diameter relationship for multiple refractive indices. For particles of known diameter, the refractive index can now be interpolated using the calculated scatter to diameter relationships. For example, a particle with a diameter of 500 nm and a side scattering intensity of 700 arbitrary units has a refractive index of 1.40. The particle diameter can be obtained from an orthogonal sizing technique. However, to relate the diameter obtained from an orthogonal sizing technique to the scattering intensity measured by FCM requires monodisperse particles, which EVs are not. Refractive index approximation based on orthogonal sizing techniques are therefore only suitable for synthetic particles and homogenously distributed biological particles such as viruses and virus‐like particles (Tang et al., 2019). Alternatively, the diameter of EVs can be measured directly by FCM using Flow‐SR or fluorescence membrane staining (Section 7.1.1) and used as input to look up the refractive index (de Rond et al., 2019; van der Pol et al., 2018).

The second approach to measure particle refractive index relies on custom‐built flow cytometers that allow for measuring angle‐resolved light‐scattering profiles per particle (Konokhova et al., 2012; Konokhova et al., 2016; Konokhova et al., 2016), as described in Section 7.1.1. The measurement of multiple light scattering angles allows differentiation between spherical and non‐spherical particles and could in principle be used to solve the thickness and refractive index of the membrane and the refractive index of the core of EVs independently.

Refractive indices are dependent on the wavelength of light. Hence, it is important to report the wavelength at which the refractive index is approximated in all refractive index approximation methods.

#### 7.2.2 Limitations

Currently, the main limitation of refractive index approximation is that the methods cannot be validated due to a lack of well‐characterized reference particles of known refractive index that provide an uncertainty statement. Although generally assumed, solid reference particles do not necessarily have the same refractive index as the bulk material (Section 2.2.3.1). To address this issue, metrological institutes are developing methods and procedures to measure the refractive index of submicron‐sized particles that do not rely on a need for reference particles (Kuiper et al., 2020).

FCM‐based refractive index approximation using the first described approach (Section 7.2.1) depends on the accuracy with which the particle diameter and light scattering signal are measured. An underestimation of the particle diameter as well as scatter signals close to the lower LoD generally cause an overestimation of the refractive index, sometimes leading to improbably high values (de Rond et al., 2018; van der Pol et al., 2021; van der Pol et al., 2014). Limitations on particle sizing are discussed in Section 7.1.2.

The main limitations of FCM‐based refractive index approximation using the second described approach (Section 7.2.1) are the reduced speed and sensitivity compared to standard flow cytometers, as discussed in detail in Section 7.1.2.

### 7.3 Antibody and antigen number approximation

Calibration of the fluorescence intensity in MESF units (Section 6.3.1) enables estimation of the number of antibodies bound per EV. If (1) the F/P is equal to one, and (2) the brightness of the fluorophore is not affected by conjugation to the antibody or by staining the sample (Section 2.2.2.2), the measured MESF values equal the number of antibodies per EV. Otherwise, the F/P and the change in brightness due to conjugation and/or staining need to be measured and considered to relate MESF to the number of antibodies per EV. Furthermore, antibodies are bivalent and therefore may bind to either one or two antigens, leading to a statistical error of at maximum 2‐fold in the determined number of antigens per EV. If better estimates are required, nanobodies or monovalent Fab fragments can be used (Section 4).

An alternative approach to estimating the number of antibodies per EV is the use of ABC beads, which express calibrated numbers of immunoglobulin binding molecules. ABC beads are first bound with the fluorophore conjugated antibody of interest and analysed under the same conditions as the EV samples. The fluorescence intensity in arbitrary units can then be related to the number of antibody capture sites on the ABC beads, thus to the number of antibodies per EV.

## 8 DATA REPORTING

Measurement results of EV FCM experiments depend on the experimental design, preanalytical variables, data acquisition settings and the applied gates. In turn, the rigor of EV FCM experiments depends on the implementation and outcome of assay controls. Therefore, it is recommended to report all details about the experimental design and preanalytical variables (Coumans et al., 2017; Théry et al., 2018), data acquisition settings, applied gates, and assay controls (Welsh et al., 2020). As flow cytometers provide data in arbitrary units and only part of all EVs can be detected, it is essential to calibrate the data and report the gates in standard units. Moreover, to evaluate the rigor and reproducibility of published data, access to the raw data of the EV samples and quality controls is important. Preferably, the Flow Cytometry Standard (FCS) file format are used and shared together with the analysis files (e.g. FlowJo .wsp files, FCS Express .fey files, or programming scripts) at a public repository.

### 8.1 EV number concentration

The concentration of EVs is a widely reported sample property, which is used to investigate EV‐related questions and to optimize and compare instrument performance, assay efficacy, and assay controls. However, the measurement of EV concentration is difficult, because it involves various sources of systematic and random errors and it depends on the sensitivity of the used detectors. For example, an important technical requirement of measuring the EV concentration is that the flow rate and sensitivity of the detectors are well‐known and stable. In addition, due to the skewed size, fluorescence, and light scattering distribution of EVs (Figure 12), small changes in the lower LoD can lead to large changes in measured EV concentrations (Section 2.1.1).

To illustrate the strong dependency of the measured EV concentrations on the lower LoD, Figure 12(A) shows the same size distribution as in Figure 2(A), but with a bin width of 10 nm and three vertical lines representing the lower LoD of three different flow cytometers. The flow cytometer with a lower LoD of 300 nm measures a concentration of 1.5·10^6^ EVs · ml^−1^, whereas the flow cytometer with a lower LoD of 150 nm measures 2.1·10^7^ EVs · ml^−1^, and the flow cytometer with a lower LoD of 100 nm measures 5.0·10^7^ EVs · ml^−1^.

Table 5 summarizes the relation between the lower LoDs of these flow cytometers and the measured EV concentrations. As the measured EV concentration depends on the lower LoD, the measured EV concentration should be reported together with the lower LoD, or preferably the dynamic range, in standard units of the used detectors. For example: “We measured 4∙10^6^ CD61+ EVs with a diameter between 180 and 1,000 nm and a fluorescence intensity between 20 and 2000 MESF PE per ml of cell‐depleted human blood plasma”. Hitherto, only a handful of studies reported the dynamic range of detectors together with the measured EV concentrations. In fact, all other studies reporting EV concentrations are irreproducible. The MIFlowCyt‐EV framework attempts to improve reproducibility and comparisons of EV concentrations in the literature through standard reporting of factors that influence the LoD, such as the dynamic range, sensitivity, and trigger threshold (Welsh et al., 2020).

**TABLE 4:** Properties of commonly used fluorophores. AF: Alexa Fluor; APC: allophycocyanin; BV: brilliant violet; eGFP: enhanced green fluorescent protein; FITC: fluorescein; MESF: molecules of equivalent solble fluorophores; PE: phycoerythrin

Fluorophore	Type	Molecular mass (kDa)	Extinction Coefficient (ε, M^−1^ cm^−1^)	Quantum Yield (ϕ)	Fluorophore brightness (ε·ϕ)	MESF calibrators
PE	Large protein	250	1.96E + 06	0.84	1.65E + 06	Bangs Laboratories, Becton Dickinson, Spherotech
BV421	Organic polymer	75	2.50E + 06	0.65	1.63E + 06	BD Special Order
APC	Large protein	105	7.00E + 05	0.68	4.76E + 05	Bangs Laboratories, BD Special Order, Spherotech
AF647	Small organic molecules	1.3	2.70E + 05	0.33	8.91E + 04	Bangs Laboratories
AF488	Small organic molecules	0.6	7.30E + 04	0.92	6.72E + 04	Bangs Laboratories
FITC	Small organic molecules	0.4	7.30E + 04	0.92	6.72E + 04	Bangs Laboratories, Spherotech
eGFP	Small protein	27	5.59E + 04	0.6	3.35E + 04	Bangs Laboratories, Spherotech

**TABLE 5:** Effect of the lower limit of detection (LoD) on the reported statistics of the size distribution and particle concentration

		Diameter (nm)			
	LoD (nm)	Median	Mean	Mode	Concentration (ml^−1^)
True population		100	114	74	1.0 · 10^8^
LoD 1	100	141	157	101	5.0 · 10^7^
LoD 2	150	188	206	206	2.1 · 10^7^
LoD 3	300	342	363	321	1.5 · 10^6^

### 8.2 EV brightness

The brightness of an EV refers to the measured fluorescence or light scattering intensity. The fluorescence intensity depends on the number of fluorophores at or within the EV, whereas the light scattering intensity depends on the (effective) scattering cross section of the EV, which in turn depends on the diameter of the EV and the refractive index distribution within the EV.

In traditional FCM analyses, the fluorescence intensity of a population of cells, which can be fully resolved from the background by FSC, is typically described by the MFI. For intensity distributions of EVs, however, the use of summarizing statistics of histograms, such as mean, median, mode, standard deviation, and confidence intervals, are unreliable data, because EVs cannot be fully resolved from the background.

To illustrate how the lower LoD of a flow cytometer affects the summarizing statistics of histograms, Figure 12(B) and (C) show a fluorescence and light scattering intensity distribution, respectively, of an EV population and the LoDs of three flow cytometers. In addition, Table 6 shows the statistics of the brightness distributions measured by the three flow cytometers. For flow cytometers with a lower LoD corresponding to the detection of 100, 150, or 300 nm EVs, the median fluorescence intensity is skewed to 3094 MESF, 5531 MESF, and 18670 MESF, respectively. With the same LoDs, the median scattering cross section is skewed to 11 nm^2^, 32 nm^2^, 158 nm^2^, respectively.

**TABLE 6:** Effect of the lower limit of detection (LoD) on the reported statistics of the fluorescence and light scattering intensities. MESF: molecules of equivalent soluble fluorophores

		Fluorescence Brightness (MESF)			Light scattering brightness (nm^2^)	
	LoD (nm)	Median	Mean	Mode	Median	Mean
True population			2611	560	3	12.49
LoD 1	100	3094	4394	1586	11	23.98
LoD 2	150	5531	7203	3571	32	47.37
LoD 3	300	18670	21551	14214	158	167.11

Given the strong dependency of statistics of EV brightness distributions on the lower LoDs, reporting raw, calibrated data along with the lower LoDs in standard units is highly recommended. Other data analysis procedures, such as normalization, smoothing, and expressing ratios or percentages, should be applied to calibrated data.

### 8.3 EV size distribution

Similar to the brightness of EVs (Section 8.2), statistical descriptions of the size distribution of EVs measured by FCM are biased by the lower LoD. Figure 12(A) shows an estimated size distribution of EVs with a median diameter of 100 nm. When a lower LoD corresponding to the detection of EVs with a diameter of 100 nm, 150 nm, or 300 nm are imposed, the median diameter is skewed to 141 nm, 188 nm, 344 nm. Table 4 shows the summarizing statistics of the size distributions measured by the three flow cytometers. Thus, the same EV population detected with three different lower LoDs results in a median statistic variability of >200 nm. Therefore, also when reporting the diameter of EVs, defining the lower LoD for a flow cytometer is critical and the use of statistics to describe size distributions should be used with caution.

## 9 CONCLUSIONS

Flow cytometers were developed in the 20^th^ century and widely used to study blood cells and many other types of particles. Compared to cells, however, EVs are challenging to characterize, because EVs have signal levels that are at or below the background noise level of flow cytometers. For this reason, many procedures frequently used for cells do not lend well for work with EVs (Table 1), and can lead to false assumptions (Table 2). Whereas entire text books have been devoted to FCM (Shapiro, 2003), specific knowledge about the application of FCM to characterize single EVs and other submicron‐sized particles have yet to be curated into a single source. This compendium, for the first time, summarizes the current knowledge, insights and good practices that are needed to set up an EV FCM experiment and generate reliable and reproducible results.

To date, best practices adopted in the EV field on the influence of pre‐analytical variables and experimental procedures are based on published EV research. Many of these studies contain uncalibrated data and are lacking in the assay controls necessary to confirm that the measured signals were originating from EVs, making these publications unreliable and irreproducible. On the other hand, during the past decade the use of single EV FCM has reached a level of understanding that is starting to allow for the generation of reliable and reproducible data. The key to generating reliable and reproducible EV FCM data are implementation of assay controls, calibration, and transparency in data reporting. To communicate this message to the field, the EV FCM working group has published an ISEV position manuscript outlining a framework of minimum information that should be reported about an EV FCM experiment (Welsh et al., 2020). This compendium complements the MIFlowCyt‐EV framework by providing background information and rationale on EV FCM.

With assay controls and calibrations in place, future work to drive this field forward should focus on the development of more sensitive hardware, improved assays, dimmer reference materials and finally, a population of the scientific research literature with calibrated data on EVs. As a first step, the benefits of calibrating all FCM aspects need to be confirmed in inter‐institutional standardization studies. These studies are already underway with initiatives such as METVES II (metves.eu) and the ISEV Rigor & Standardization subcommittee EV Reference Material Task Force. To create more practical and accurate FCM assays, engagement of the research field with industry and metrological agencies to develop and test improved reference materials as calibrators, validators, and quality control materials for single EV assays is of key importance for the field (Geeurickx et al., 2019; Lozano‐Andrés et al., 2019; Tang et al., 2019; Welsh et al., 2020). The EV FCM working group will continue to assist new initiatives where possible, such as promoting and facilitating the utilization of standardized, transparent reporting for single EV FCM experiments, curating a resource of standardized EV FCM data, generating educational materials to aid in the performing and reporting of single EV FCM assays, and keep abreast of current knowledge to update the MIFlowCyt‐EV framework and this compendium.

## CONFLICT OF INTEREST

André Görgens has equity interest in and is a consultant for Evox Therapeutics Ltd, Oxford, UK. André Görgens is inventor on patents and patent applications related to extracellular vesicle engineering, manufacturing, and analysis. An Hendrix is an inventor on patents and patent applications related to extracellular vesicle products. Edwin van der Pol is co‐founder and shareholder of Exometry BV. Joshua A. Welsh and Jennifer C. Jones are inventors on NIH patents and patent applications related to extracellular vesicle assays. John P. Nolan is CEO of Cellarcus Biosciences. Joanne Lannigan is a paid consultant for EV development work for Cytek Biosciences. Romaric Lacroix and Françoise Dignat‐George declare that they have patents on extracellular vesicles assays and have received funding from the companies Stago and Beckman Coulter. Xiaomei Yan declares a competing financial interest as a cofounder and shareholder of NanoFCM Inc.

## Supporting information

Supplemental materials 1: Video to provide insight into the detection of light scattered by extracellular vesicles (EVs). Effective forward scattering cross section versus diameter (left), angular light scattering distribution and collection angles of the forward scattered light (FSC) and side scattered light (SSC) detectors (middle), and effective side scattering cross section versus the diameter (right) of EVs (Part 1) and of EVs, polystyrene beads, and silica beads (Part 2). Calculations were done with Mie theory using an illumination wavelength of 405 nm. EVs were modelled as core‐shell particles with a shell refractive index of 1.48, a shell thickness of 5 nm, and a core refractive index of 1.38. Polystyrene beads and silica were modelled as solid spheres with a refractive index of 1.63 and 1.46, respectively. The refractive index of the medium was 1.34. The half‐angle of light collection was 15° and 55° for FSC and SSC, respectively.

## ENDNOTES

1 Besides molecules, such as the fluorophores that are commonly used in FCM, also atoms and nanostructures, such as quantum dots, may exhibit fluorescence.

2 For clarity, Figure 4 shows only one electronic excited state. In reality, however, a molecule can have multiple electronic excited states.

3 The units of ε are M^‐1^·m^‐1^, which is equivalent to m^2^·mol^‐1^. The units of M^‐1^·m^‐1^ reveal that when light propagates through a suspension of fluorophores, then ε is the attenuation of light per m per molar concentration of the fluorophores. Hence, ε can be determined with an optical density measurement.

^4^
Under the condition that the illumination irradiance is sufficiently high to reach near photon saturation.

5 In addition to light, also a chemical reaction may irreversibly modify the chemical structure of a fluorophore. This process is called bleaching.

6 Raman scattering causes an energy gain or loss of the light scattered by a particle. However, the Raman effect is typically more than a million‐fold weaker than light scattered without energy gain or loss.

7 Based on the total scattering cross section of a solid sphere with a diameter of 210 nm in water calculated with Mie theory (Section 2.2.3.4). The modelled illumination wavelength is 405 nm.

8 In this section, the units for power and area are W and m^2^, respectively. In practise, however, laser powers are expressed in mW, laser beams are focused to an area of several μm^2^, and the effective scattering cross section of EVs is expressed in nm^2^.

9 Equation 3 is only valid for a focus with a homogeneous irradiance profile, such as the case for flat‐top illumination. In most flow cytometers, however, the irradiance profile of a focused laser beam has a Gaussian distribution, which changes Equation 3.

10 Rayleigh scattering assumes that the scattered intensity of the individual charges of a particle can be summed, because the charges radiate in phase, which results in constructive interference between the scattered light waves. When the distance between charges of particle exceeds ∼1/10^th^ the illumination wavelength, however, the charges do not radiate in phase and besides constructive interference, also destructive interference takes place, making Rayleigh theory inapplicable.

11 CD61+ EVs with a diameter >200 nm have a median effective refractive index of 1.37 (van der Pol et al., 2018).

12 for flow cytometers that are designed to characterize cells.

13 Engineers call a stable electrical offset the direct current (DC) component.

14 In traditional flow cytometers, particles typically are illuminated by spatially separated lasers that are aligned at different positions along the sample flow (section 2.3.2.2).

15 A spectrometer is an instrument that separates light by wavelength using diffraction gratings or prisms.

16 Polyvalent means that one fluorophore (or QD) binds multiple antibodies

17 Nucleic acid staining of cells results in dim fluorescence signals on commercial flow cytometers. Under the assumption that the nucleic acid concentration scales proportionally with volume, the concentration of nucleic acids in EVs and hence the anticipated fluorescence signal is 4 to 7 orders of magnitude lower compared to cells.

18 Although in cellular FCM compensation can be performed with stained cells instead of compensation beads, it is not recommended to use EVs to set up a compensation matrix, given the heterogeneity and dim signal intensities of EVs.

19 Methods that quantify the fluorescence sensitivity and dynamic range of a flow cytometer are beyond the scope of this educational manuscript. These methods do however allow for accurate reporting and data comparison, as well as instrument performance tracking over time (Giesecke et al., 2017; Parks et al., 2017; Wang & Hoffman, 2017; Wood, 1998).

20 Other standard units in FCM involve the number of detected photoelectrons and equivalent reference fluorophores (ERF) [40; Wood, 1998; Parks et al., 2017; Steen, 1992; Chase & Hoffman, 1998; Hoffman & Wood]. Estimation of the number of photoelectrons can be useful in instrument design, optimization, and characterization, whereas ERF units may be useful in cases where ABC or MESF assignments are not relevant or possible, such as generic fluorescent staining.

## References

[jev212299-bib-0001] Aalberts, M. , van Dissel‐Emiliani, F. M. F. , van Adrichem, N. P. H. , van Wijnen, M. , Wauben, M. H. M. , Stout, T. A. E. , & Stoorvogel, W. (2012). Identification of distinct populations of prostasomes that differentially express prostate stem cell antigen, annexin A1, and GLIPR2 in humans. Biology of Reproduction, 86, 82. 22133690 10.1095/biolreprod.111.095760

[jev212299-bib-0002] Arraud, N. , Gounou, C. , Turpin, D. , & Brisson, A. R. (2016). Fluorescence triggering: A general strategy for enumerating and phenotyping extracellular vesicles by flow cytometry. Cytometry Part A, 89, 184–195. 10.1002/cyto.a.22669 25857288

[jev212299-bib-0003] Arraud, N. , Linares, R. , Tan, S. , Gounou, C. , Pasquet, J. M. , Mornet, S. , & Brisson, A. R. (2014). Extracellular vesicles from blood plasma: determination of their morphology, size, phenotype and concentration. Journal of Thrombosis and Haemostasis, 12, 614–627. 24618123 10.1111/jth.12554

[jev212299-bib-0004] Austin Suthanthiraraj, P. P. , & Graves, S. W. (2013). Fluidics. Current Protocols in Cytometry, 65, 1. 10.1002/0471142956.cy0102s65 PMC4035220 23835801

[jev212299-bib-0005] Barranco, I. , Padilla, L. , Parrilla, I. , Álvarez‐Barrientos, A. , Pérez‐Patiño, C. , Peña, F. J. , Martínez, E. A. , Rodriguez‐Martínez, H. , & Roca, J. (2019). Extracellular vesicles isolated from porcine seminal plasma exhibit different tetraspanin expression profiles. Scientific Reports, 9, 1. 31399634 10.1038/s41598-019-48095-3 PMC6689046

[jev212299-bib-0006] Bera, D. , Qian, L. , Tseng, T.‐K. , & Holloway, P. H. (2010). Quantum dots and their multimodal applications: A review. Materials (Basel), 3, 2260–2345.

[jev212299-bib-0007] Berckmans, R. J. , Lacroix, R. , Hau, C. M. , Sturk, A. , & Nieuwland, R. (2019). Extracellular vesicles and coagulation in blood from healthy humans revisited. Journal of Extracellular Vesicles, 8, 1688936. 31762964 10.1080/20013078.2019.1688936 PMC6853244

[jev212299-bib-0008] Bettin, B. , Gasecka, A. , Li, B. , Dhondt, B. , Hendrix, A. , Nieuwland, R. , & van der Pol, E. (2022). Removal of platelets from blood plasma to improve the quality of extracellular vesicle research. Journal of Thrombosis and Haemostasis, 20(11), 2679–2685. 36043239 10.1111/jth.15867 PMC9825910

[jev212299-bib-0009] Biller, S. J. , Schubotz, F. , Roggensack, S. E. , Thompson, A. W. , Summons, R. E. , & Chisholm, S. W. (2019). Bacterial vesicles in marine ecosystems. Science, 343, 183. 10.1126/science.1243457 24408433

[jev212299-bib-0010] Bohren, C. F. , & Huffman, D. R. (2014). Absorption and scattering of light by small particles. 1st ed.

[jev212299-bib-0011] Böing, A. N. , van der Pol, E. , Grootemaat, A. E. , Coumans, F. A. W. , Sturk, A. , & Nieuwland, R. (2014). Single‐step isolation of extracellular vesicles by size‐exclusion chromatography. Journal of Extracellular Vesicles, 3, 23430. 10.3402/jev.v3.23430 PMC4159761 25279113

[jev212299-bib-0012] Böttcher, S. , van der Velden, V. H. J. , Villamor, N. , Ritgen, M. , Flores‐Montero, J. , Murua Escobar, H. , Kalina, T. , Brüggemann, M. , Grigore, G. , Martin‐Ayuso, M. , Lecrevisse, Q. , Pedreira, C. E. , van Dongen, J. J. M. , & Orfao, A. (2019). Lot‐to‐lot stability of antibody reagents for flow cytometry. Journal of Immunological Methods, 475, 112294. 28365329 10.1016/j.jim.2017.03.018

[jev212299-bib-0013] Brisson, A. R. , Tan, S. , Linares, R. , Gounou, C. , & Arraud, N. (2017). Extracellular vesicles from activated platelets: A semiquantitative cryo‐electron microscopy and immuno‐gold labeling study. Platelets, 28, 263–271. 28102751 10.1080/09537104.2016.1268255

[jev212299-bib-0014] Bruce, V. J. , & McNaughton, B. R. (2017). Evaluation of nanobody conjugates and protein fusions as bioanalytical reagents. Analytical Chemistry, 89, 3819–3823. 28316235 10.1021/acs.analchem.7b00470 PMC5796518

[jev212299-bib-0015] Brunsting, A. , & Mullaney, P. F. (1974). Differential light scattering from spherical mammalian cells. Biophysical Journal, 14, 439–453. 4134589 10.1016/S0006-3495(74)85925-4 PMC1334522

[jev212299-bib-0016] Busatto, S. , Vilanilam, G. , Ticer, T. , Lin, W.‐L. , Dickson, D. W. , Shapiro, S. , Bergese, P. , & Wolfram, J. (2018). Tangential flow filtration for highly efficient concentration of extracellular vesicles from large volumes of fluid. Cells, 7, 273. 30558352 10.3390/cells7120273 PMC6315734

[jev212299-bib-0017] Cai, Q. , Qiao, L. , Wang, M. , He, B. , Lin, F.‐M. , Palmquist, J. , Huang, S.‐D. , & Jin, H. (2018). Plants send small RNAs in extracellular vesicles to fungal pathogen to silence virulence genes. Science, 360, 1126–1129. 29773668 10.1126/science.aar4142 PMC6442475

[jev212299-bib-0018] Cantin, R. , Diou, J. , Bélanger, D. , Tremblay, A. M. , & Gilbert, C. (2008). Discrimination between exosomes and HIV‐1: Purification of both vesicles from cell‐free supernatants. Journal of Immunological Methods, 338, 21–30. 18675270 10.1016/j.jim.2008.07.007

[jev212299-bib-0019] Chapple, M. R. , Johnson, G. D. , & Davidson, R. S. (1990). Fluorescence quenching; a practical problem in flow cytometry. Journal of Microscopy, 159, 245–253. 1700820 10.1111/j.1365-2818.1990.tb03030.x

[jev212299-bib-0020] Chase, E. S. , & Hoffman, R. A. (1998). Resolution of dimly fluorescent particles: A practical measure of fluorescence sensitivity. Cytometry, 33, 267–279. 9773890

[jev212299-bib-0021] Chattopadhyay, P. K. , Gaylord, B. , Palmer, A. , Jiang, N. , Raven, M. A. , Lewis, G. , Reuter, M. A. , Nur‐ur Rahman, A. K. M. , Price, D. A. , Betts, M. R. , & Roederer, M. (2012). Brilliant violet fluorophores: A new class of ultrabright fluorescent compounds for immunofluorescence experiments. Cytometry Part A, 81, 456–466. 10.1002/cyto.a.22043 22489009

[jev212299-bib-0022] Chen, D. , Wu, I.‐C. , Liu, Z. , Tang, Y. , Chen, H. , Yu, J. , Wu, C. , & Chiu, D. T. (2017). Semiconducting polymer dots with bright narrow‐band emission at 800 nm for biological applications. Chemical Science, 8, 3390–3398. 28507710 10.1039/c7sc00441a PMC5416912

[jev212299-bib-0023] Cimorelli, M. , Nieuwland, R. , Varga, Z. , & van der Pol, E. (2021). Standardized procedure to measure the size distribution of extracellular vesicles together with other particles in biofluids with microfluidic resistive pulse sensing. PLoS ONE, 16, e0249603. 33793681 10.1371/journal.pone.0249603 PMC8016234

[jev212299-bib-0024] Cloutier, N. , Tan, S. , Boudreau, L. H. , Cramb, C. , Subbaiah, R. , Lahey, L. , Albert, A. , Shnayder, R. , Gobezie, R. , Nigrovic, P. A. , Farndale, R. W. , Robinson, W. H. , Brisson, A. , Lee, D. M. , & Boilard, E. (2013). The exposure of autoantigens by microparticles underlies the formation of potent inflammatory components: The microparticle‐associated immune complexes. EMBO Molecular Medicine, 5, 235–249. 23165896 10.1002/emmm.201201846 PMC3569640

[jev212299-bib-0025] Collot, M. , Ashokkumar, P. , Anton, H. , Boutant, E. , Faklaris, O. , Galli, T. , Mély, Y. , Danglot, L. , & Klymchenko, A. S. (2019). MemBright: A family of fluorescent membrane probes for advanced cellular imaging and neuroscience. Cell Chemical Biology, 26, 600–614.e7. 30745238 10.1016/j.chembiol.2019.01.009

[jev212299-bib-0026] Corso, G. , Heusermann, W. , Trojer, D. , Görgens, A. , Steib, E. , Voshol, J. , Graff, A. , Genoud, C. , Lee, Y. , Hean, J. , Nordin, J. Z. , Wiklander, O. P. B. , El Andaloussi, S. , & Meisner‐Kober, N. (2019). Systematic characterization of extracellular vesicle sorting domains and quantification at the single molecule–single vesicle level by fluorescence correlation spectroscopy and single particle imaging. Journal of Extracellular Vesicles, 8, 1663043. 31579435 10.1080/20013078.2019.1663043 PMC6758720

[jev212299-bib-0027] Corso, G. , Mäger, I. , Lee, Y. , Görgens, A. , Bultema, J. , Giebel, B. , Wood, M. J. A. , Nordin, J. Z. , & Andaloussi, S. E. L. (2017). Reproducible and scalable purification of extracellular vesicles using combined bind‐elute and size exclusion chromatography. Scientific Reports, 7, 11561. 28912498 10.1038/s41598-017-10646-x PMC5599601

[jev212299-bib-0028] Cossarizza, A. , Chang, H. , Radbruch, A. , Acs, A. , Adam, D. , Adam‐Klages, S. , Agace, W. W. , Aghaeepour, N. , Akdis, M. , Allez, M. , Almeida, L. N. , Alvisi, G. , Anderson, G. , Andrä, I. , Annunziato, F. , Anselmo, A. , Bacher, P. , Baldari, C. T. , Bari, S. , … Zychlinsky, A. (2019). Guidelines for the use of flow cytometry and cell sorting in immunological studies (Second Edition). European Journal of Immunology, 49, 1457–1973. 31633216 10.1002/eji.201970107 PMC7350392

[jev212299-bib-0029] Coumans, F. A. W. , Brisson, A. R. , Buzas, E. I. , Dignat‐George, F. , Drees, E. E. E. , El‐Andaloussi, S. , Emanueli, C. , Gasecka, A. , Hendrix, A. , Hill, A. F. , Lacroix, R. , Lee, Y. , van Leeuwen, T. G. , Mackman, N. , Mäger, I. , Nolan, J. P. , van der Pol, E. , Pegtel, D. M. , Sahoo, S. , … Nieuwland, R. (2017). Methodological guidelines to study extracellular vesicles. Circulation Research, 120, 1632–1648. 28495994 10.1161/CIRCRESAHA.117.309417

[jev212299-bib-0030] Coumans, F. A. W. , van der Pol, E. , Böing, A. N. , Hajji, N. , Sturk, G. , van Leeuwen, T. G. , & Nieuwland, R. (2014). Reproducible extracellular vesicle size and concentration determination with tunable resistive pulse sensing. Journal of Extracellular Vesicles, 3, 25922. 25498889 10.3402/jev.v3.25922 PMC4263901

[jev212299-bib-0031] Coumans, F. A. W. , van der Pol, E. , & Terstappen, L. W. M. M. (2012). Flat‐top illumination profile in an epifluorescence microscope by dual microlens arrays. Cytometry Part A, 81A, 324–331. 10.1002/cyto.a.22029 22392641

[jev212299-bib-0032] Curl, C. L. , Bellair, C. J. , Harris, T. , Allman, B. E. , Harris, P. J. , Stewart, A. G. , Roberts, A. , Nugent, K. A. , & Delbridge, L. M. D. (2005). Refractive index measurement in viable cells using quantitative phase‐amplitude microscopy and confocal microscopy. Cytometry Part A, 65, 88–92. 10.1002/cyto.a.20134 15800856

[jev212299-bib-0033] Cvjetkovic, A. , Lötvall, J. , & Lässer, C. (2014). The influence of rotor type and centrifugation time on the yield and purity of extracellular vesicles. Journal of Extracellular Vesicles, 3, 23111. 10.3402/jev.v3.23111 PMC3967015 24678386

[jev212299-bib-0034] Daimon, M. , & Masumura, A. (2007). Measurement of the refractive index of distilled water from the near‐infrared region to the ultraviolet region. Applied Optics, 46, 3811. 17538678 10.1364/ao.46.003811

[jev212299-bib-0035] Davies, R. T. , Kim, J. , Jang, S. C. , Choi, E.‐J. , Gho, Y. S. , & Park, J. (2012). Microfluidic filtration system to isolate extracellular vesicles from blood. Lab on a Chip, 12, 5202. 23111789 10.1039/c2lc41006k

[jev212299-bib-0036] Deka, C. , Lehnert, B. E. , Lehnert, N. M. , Jones, G. M. , Sklar, L. A. , & Steinkamp, J. A. (1996). Analysis of fluorescence lifetime and quenching of FITC‐conjugated antibodies on cells by phase‐sensitive flow cytometry. Cytometry, 25, 271–279. 8914824 10.1002/(SICI)1097-0320(19961101)25:3<271::AID-CYTO8>3.0.CO;2-I

[jev212299-bib-0037] Dempsey, G. T. , Vaughan, J. C. , Chen, K. H. , Bates, M. , & Zhuang, X. (2011). Evaluation of fluorophores for optimal performance in localization‐based super‐resolution imaging. Nature Methods, 8, 1027–1036. 22056676 10.1038/nmeth.1768 PMC3272503

[jev212299-bib-0038] de Rond, L. , Coumans, F. A. W. , Nieuwland, R. , van Leeuwen, T. G. , & van der Pol, E. (2018). Deriving extracellular vesicle size from scatter intensities measured by flow cytometry. Current Protocols in Cytometry, e43, 1. 10.1002/cpcy.43 30168659

[jev212299-bib-0039] de Rond, L. , Coumans, F. A. W. , Welsh, J. A. , Nieuwland, R. , van Leeuwen, T. G. , & van der Pol, E. (2020). Quantification of light scattering detection efficiency and background in flow cytometry. Cytometry Part A, 99, 671–679. 10.1002/cyto.a.24243 PMC8359315 33085220

[jev212299-bib-0040] de Rond, L. , Libregts, S. F. W. M. , Rikkert, L. G. , Hau, C. M. , van der Pol, E. , Nieuwland, R. , van Leeuwen, T. G. , & Coumans, F. A. W. (2019). Refractive index to evaluate staining specificity of extracellular vesicles by flow cytometry. Journal of Extracellular Vesicles, 8, 1643671. 31489142 10.1080/20013078.2019.1643671 PMC6713200

[jev212299-bib-0041] de Rond, L. , van der Pol, E. , Bloemen, P. R. , van den Broeck, T. , Monheim, L. , Nieuwland, R. , van Leeuwen, T. G. , & Coumans, F. A. W. (2020). A systematic approach to improve scatter sensitivity of a flow cytometer for detection of extracellular vesicles. Cytometry Part A, 97, 582–591. 10.1002/cyto.a.23974 PMC7383638 32017331

[jev212299-bib-0042] de Rond, L. , van der Pol, E. , Hau, C. M. , Varga, Z. , Sturk, A. , van Leeuwen, T. G. , Nieuwland, R. , & Coumans, F. A. W. (2018). Comparison of generic fluorescent markers for detection of extracellular vesicles by flow cytometry. Clinical Chemistry, 64, 680–689. 29453194 10.1373/clinchem.2017.278978

[jev212299-bib-0043] der Vlist, E. J. , Arkesteijn, G. J. A. , van de Lest, C. H. A. , Stoorvogel, W. , Nolte‐’t Hoen, E. N. M. , & Wauben, M. H. M. (2012). CD4+ T cell activation promotes the differential release of distinct populations of nanosized vesicles. Journal of Extracellular Vesicles, 1, 18364. 10.3402/jev.v1i0.18364 PMC3760647 24009884

[jev212299-bib-0044] Dominkuš, P. P. , Stenovec, M. , Sitar, S. , Lasič, E. , Zorec, R. , Plemenitaš, A. , Žagar, E. , Kreft, M. , & Lenassi, M. (2018). PKH26 Labeling of Extracellular Vesicles: Characterization and Cellular Internalization of Contaminating PKH26 Nanoparticles. Biochimica et Biophysica Acta ‐ Biomembranes, 1860, 1350–1361. 29551275 10.1016/j.bbamem.2018.03.013

[jev212299-bib-0045] Ducharme, D. , Max, J. J. , Salesse, C. , & Leblanc, R. M. (1990). Ellipsometric study of the physical states of phosphatidylcholines at the air‐water interface. Journal of Physical Chemistry, 94, 1925–1932.

[jev212299-bib-0046] Fox, M. H. , & Coulter, J. R. (1980). Enhanced light collection in a flow cytometer. Cytometry, 1, 21–25. 7273961 10.1002/cyto.990010106

[jev212299-bib-0047] Gaigalas, A. K. , Wang, L. , Schwartz, A. , Marti, G. E. , & Vogt Jr, R. F. (2005). Quantitating fluorescence intensity from fluorophore: assignment of MESF values. Journal of research of the National Institute of Standards and Technology, 110, 101. 27308107 10.6028/jres.110.010 PMC4847572

[jev212299-bib-0048] Gankema, A. A. F. , Li, B. , Nieuwland, R. , & van der Pol, E. (2022). Automated fluorescence gating and size determination reduce variation in measured concentration of extracellular vesicles by flow cytometry. Cytometry Part A, 101(12), 1049–1056. 10.1002/cyto.a.24665 PMC10084316 35707999

[jev212299-bib-0049] Gardiner, C. , Shaw, M. , Hole, P. , Smith, J. , Tannetta, D. , Redman, C. W. , & Sargent, I. L. (2014). Measurement of refractive index by nanoparticle tracking analysis reveals heterogeneity in extracellular vesicles. Journal of Extracellular Vesicles, 3, 25361. 25425324 10.3402/jev.v3.25361 PMC4247498

[jev212299-bib-0050] Gasecka, A. , Böing, A. N. , Filipiak, K. J. , & Nieuwland, R. (2017). Platelet extracellular vesicles as biomarkers for arterial thrombosis. Platelets, 28, 228–234. 27996341 10.1080/09537104.2016.1254174

[jev212299-bib-0051] Gasecka, A. , Nieuwland, R. , Budnik, M. , Dignat‐George, F. , Eyileten, C. , Harrison, P. , Lacroix, R. , Leroyer, A. , Opolski, G. , Pluta, K. , Pol, E. , Postuła, M. , Siljander, P. , Siller‐Matula, J. M. , & Filipiak, K. J. (2020). Ticagrelor attenuates the increase of extracellular vesicle concentrations in plasma after acute myocardial infarction compared to clopidogrel. Journal of Thrombosis and Haemostasis, 18, 609–623. 31833175 10.1111/jth.14689 PMC7065161

[jev212299-bib-0052] Geeurickx, E. , Tulkens, J. , Dhondt, B. , Van Deun, J. , Lippens, L. , Vergauwen, G. , Heyrman, E. , De Sutter, D. , Gevaert, K. , Impens, F. , Miinalainen, I. , Van Bockstal, P.‐J. , De Beer, T. , Wauben, M. H. M. , Nolte‐‘t‐Hoen, E. N. M. , Bloch, K. , Swinnen, J. V. , van der Pol, E. , Nieuwland, R. , … Hendrix, A. (2019). The generation and use of recombinant extracellular vesicles as biological reference material. Nature Communications, 10, 3288. 10.1038/s41467-019-11182-0 PMC6650486 31337761

[jev212299-bib-0053] Ghosh, N. , Buddhiwant, P. , Uppal, A. , Majumder, S. K. , Patel, H. S. , & Gupta, P. K. (2006). Simultaneous determination of size and refractive index of red blood cells by light scattering measurements. Applied Physics Letters, 88, 84101.

[jev212299-bib-0054] Giesecke, C. , Feher, K. , von Volkmann, K. , Kirsch, J. , Radbruch, A. , & Kaiser, T. (2017). Determination of background, signal‐to‐noise, and dynamic range of a flow cytometer: A novel practical method for instrument characterization and standardization. Cytometry Part A, 91, 1104–1114. 10.1002/cyto.a.23250 28960720

[jev212299-bib-0055] Görgens, A. , Bremer, M. , Ferrer‐Tur, R. , Murke, F. , Tertel, T. , Horn, P. A. , Thalmann, S. , Welsh, J. A. , Probst, C. , Guerin, C. , Boulanger, C. M. , Jones, J. C. , Hanenberg, H. , Erdbrügger, U. , Lannigan, J. , Ricklefs, F. L. , El‐Andaloussi, S. , & Giebel, B. (2019). Optimisation of imaging flow cytometry for the analysis of single extracellular vesicles by using fluorescence‐tagged vesicles as biological reference material. Journal of Extracellular Vesicles, 8, 1587567. 30949308 10.1080/20013078.2019.1587567 PMC6442110

[jev212299-bib-0056] György, B. , Módos, K. , Pállinger, É. , Pálóczi, K. , Pásztói, M. , Misják, P. , Deli, M. A. , Sipos, Á. , Szalai, A. , Voszka, I. , Polgár, A. , Tóth, K. , Csete, M. , Nagy, G. , Gay, S. , Falus, A. , Kittel, Á. , & Buzás, E. I. (2011). Detection and isolation of cell‐derived microparticles are compromised by protein complexes resulting from shared biophysical parameters. Blood, 117, e39–e48. 21041717 10.1182/blood-2010-09-307595

[jev212299-bib-0057] György, B. , Szabó, T. G. , Turiák, L. , Wright, M. , Herczeg, P. , Lédeczi, Z. , Kittel, Á. , Polgár, A. , Tóth, K. , Dérfalvi, B. , Zelenák, G. , Böröcz, I. , Carr, B. , Nagy, G. , Vékey, K. , Gay, S. , Falus, A. , & Buzás, E. I. (2012). Improved flow cytometric assessment reveals distinct microvesicle (cell‐derived microparticle) signatures in joint diseases. PLoS ONE, 7, 1. 10.1371/journal.pone.0049726 PMC3502255 23185418

[jev212299-bib-0058] Han, Y. , Gu, Y. , Zhang, A. C. , & Lo, Y.‐H. (2016). Imaging technologies for flow cytometry. Lab on a Chip, 16, 4639–4647. 27830849 10.1039/c6lc01063f PMC5311077

[jev212299-bib-0059] Hecht, E. (2001). Optics. 4th ed. San Francisco, USA.

[jev212299-bib-0060] Hoen, E. N. M. N.‐’t , van der Vlist, E. J. , Aalberts, M. , Mertens, H. C. H. , Bosch, B. J. , Bartelink, W. , Mastrobattista, E. , van Gaal, E. V. B. , Stoorvogel, W. , Arkesteijn, G. J. A. , & Wauben, M. H. M. (2012). Quantitative and qualitative flow cytometric analysis of nanosized cell‐derived membrane vesicles. Nanomedicine: Nanotechnology, Biology and Medicine, 8, 712–720. 22024193 10.1016/j.nano.2011.09.006 PMC7106164

[jev212299-bib-0061] Hoffman, R. A. , & Wood, J. C. S. (2007). Characterization of flow cytometer instrument sensitivity. Current Protocols in Cytometry, 40, 1. 10.1002/0471142956.cy0120s40 18770846

[jev212299-bib-0062] Horvath, R. , Fricsovszky, G. , & Papp, E. (2003). Application of the optical waveguide lightmode spectroscopy to monitor lipid bilayer phase transition. Biosensors & Bioelectronics, 18, 415–428. 12604259 10.1016/s0956-5663(02)00154-9

[jev212299-bib-0063] Jayachandran, M. , Miller, V. M. , Heit, J. A. , & Owen, W. G. (2012). Methodology for isolation, identification and characterization of microvesicles in peripheral blood. Journal of Immunological Methods, 375, 207–214. 22075275 10.1016/j.jim.2011.10.012 PMC3253871

[jev212299-bib-0064] Jeyarajah, E. J. , Cromwell, W. C. , & Otvos, J. D. (2006). Lipoprotein particle analysis by nuclear magnetic resonance spectroscopy. Clinics in Laboratory Medicine, 26, 847–870. 17110242 10.1016/j.cll.2006.07.006

[jev212299-bib-0065] Jin, Y. , Ye, F. , Wu, C. , Chan, Y.‐H. , & Chiu, D. T. (2012). Generation of functionalized and robust semiconducting polymer dots with polyelectrolytes. Chemical Communications, 48, 3161. 22349364 10.1039/c2cc17703j PMC3819213

[jev212299-bib-0066] Johnson, S. M. , Banyard, A. , Smith, C. , Mironov, A. , & McCabe, M. G. (2020). Large extracellular vesicles can be characterised by multiplex labelling using imaging flow cytometry. International Journal of Molecular Sciences, 21, 8723. 33218198 10.3390/ijms21228723 PMC7699300

[jev212299-bib-0067] Joint Committee for Guides in Metrology (JGCM) . (2012). International Vocabulary of Metrology – Basic and General Concepts and Associated Terms (VIM), JGCM.

[jev212299-bib-0068] Jung, S.‐R. , Han, R. , Sun, W. , Jiang, Y. , Fujimoto, B. S. , Yu, J. , Kuo, C.‐T. , Rong, Y. , Zhou, X.‐H. , & Chiu, D. T. (2018). Single‐molecule flow platform for the quantification of biomolecules attached to single nanoparticles. Analytical Chemistry, 90, 6089–6095. 29672026 10.1021/acs.analchem.8b00024 PMC5953847

[jev212299-bib-0069] Kalina, T. , Lundsten, K. , & Engel, P. (2020). Relevance of antibody validation for flow cytometry. Cytometry Part A, 97, 126. 10.1002/cyto.a.23895 31577065

[jev212299-bib-0070] Kasarova, S. N. , Sultanova, N. G. , Ivanov, C. D. , & Nikolov, I. D. (2007). Analysis of the dispersion of optical plastic materials. Optical Materials, 29, 1481–1490.

[jev212299-bib-0071] Khamsi, R. (2020). Plant vesicles inspire methods to protect crops. Nature, 582, S19.

[jev212299-bib-0072] Kienle, D. F. , De Souza, J. V. , Watkins, E. B. , & Kuhl, T. L. (2014). Thickness and Refractive Index of DPPC and DPPE Monolayers by Multiple‐Beam Interferometry. Analytical and bioanalytical chemistry, 406, 4725–4733. 24842403 10.1007/s00216-014-7866-9

[jev212299-bib-0073] Konokhova, A. I. , Chernova, D. N. , Moskalensky, A. E. , Strokotov, D. I. , Yurkin, M. A. , Chernyshev, A. V. , & Maltsev, V. P. (2016). Super‐resolved calibration‐free flow cytometric characterization of platelets and cell‐derived microparticles in platelet‐rich plasma. Cytometry Part A, 89, 159–168. 10.1002/cyto.a.22621 25808430

[jev212299-bib-0074] Konokhova, A. I. , Chernova, D. N. , Strokotov, D. I. , Karpenko, A. A. , Chernyshev, A. V. , Maltsev, V. P. , & Yurkin, M. A. (2016). Light‐scattering gating and characterization of plasma microparticles. Journal of Biomedial Optics, 21, 115003. 10.1117/1.JBO.21.11.115003 27893088

[jev212299-bib-0075] Konokhova, A. I. , Yurkin, M. a. , Moskalensky, A. E. , Chernyshev, A. V. , Tsvetovskaya, G. a. , Chikova, E. D. , & Maltsev, V. P. (2012). Light‐scattering flow cytometry for identification and characterization of blood microparticles. Journal of Biomedial Optics, 17, 057006. 10.1117/1.JBO.17.5.057006 22612145

[jev212299-bib-0076] Kormelink, T. G. , Arkesteijn, G. J. A. , Nauwelaers, F. A. , van den Engh, G. , Nolte‐’t Hoen, E. N. M. , & Wauben, M. H. M. (2016). Prerequisites for the analysis and sorting of extracellular vesicle subpopulations by high‐resolution flow cytometry. Cytometry Part A, 89, 135–147. 10.1002/cyto.a.22644 25688721

[jev212299-bib-0077] Kornilov, R. , Puhka, M. , Mannerström, B. , Hiidenmaa, H. , Peltoniemi, H. , Siljander, P. , Seppänen‐Kaijansinkko, R. , & Kaur, S. (2018). Efficient ultrafiltration‐based protocol to deplete extracellular vesicles from fetal bovine serum. Journal of Extracellular Vesicles, 7, 1422674. 29410778 10.1080/20013078.2017.1422674 PMC5795649

[jev212299-bib-0078] Kuchinskiene, Z. , & Carlson, L. A. (1982). Composition, concentration, and size of low density lipoproteins and of subfractions of very low density lipoproteins from serum of normal men and women. Journal of Lipid Research, 23, 762–769. 7119573

[jev212299-bib-0079] Kuiper, M. , van de Nes, A. , Nieuwland, R. , Varga, Z. , & van der Pol, E. (2020). Reliable measurements of extracellular vesicles by clinical flow cytometry. American Journal of Reproductive Immunology, 85, e13350. 32966654 10.1111/aji.13350 PMC7900981

[jev212299-bib-0080] Kuiper, M. W. , Koops, R. , Nieuwland, R. , & van der Pol, E. (2022). Method to traceably determine the refractive index by measuring the angle of minimum deviation. Metrologia, 055006. https://iopscience.iop.org/article/10.1088/1681-7575/ac8991

[jev212299-bib-0081] Lai, R. C. , Arslan, F. , Lee, M. M. , Sze, N. S. K. , Choo, A. , Chen, T. S. , Salto‐Tellez, M. , Timmers, L. , Lee, C. N. , El Oakley, R. M. , Pasterkamp, G. , de Kleijn, D. P. V. , & Lim, S. K. (2010). Exosome secreted by MSC reduces myocardial ischemia/reperfusion injury. Stem Cell Research, 4, 214–222. 20138817 10.1016/j.scr.2009.12.003

[jev212299-bib-0082] Lakowicz, J. R. (2006). Principles of fluorescence spectroscopy. 3rd ed.

[jev212299-bib-0083] Larson, M. C. , Luthi, M. R. , Hogg, N. , & Hillery, C. A. (2013). Calcium‐phosphate microprecipitates mimic microparticles when examined with flow cytometry. Cytometry Part A, 83, 242–250. 10.1002/cyto.a.22222 PMC3615643 23125136

[jev212299-bib-0084] Lewis, B. A. , & Engelman, D. M. (1983). Lipid bilayer thickness varies linearly with acyl chain length in fluid phosphatidylcholine vesicles. Journal of Molecular Biology, 166, 211–217. 6854644 10.1016/s0022-2836(83)80007-2

[jev212299-bib-0085] Libregts, S. , Arkesteijn, G. J. A. , Németh, A. , Nolte‐’t Hoen, E. N. M. , & Wauben, M. H. M. (2018). Flow cytometric analysis of extracellular vesicle subsets in plasma: Impact of swarm by particles of non‐interest. Journal of Thrombosis and Haemostasis, 16, 1423–1436. 29781099 10.1111/jth.14154

[jev212299-bib-0086] Linares, R. , Tan, S. , Gounou, C. , Arraud, N. , & Brisson, A. R. (2015). High‐speed centrifugation induces aggregation of extracellular vesicles. Journal of Extracellular Vesicles, 4, 29509. 26700615 10.3402/jev.v4.29509 PMC4689953

[jev212299-bib-0087] Liu, H. , Tian, Y. , Xue, C. , Niu, Q. , Chen, C. , & Yan, X. (2022). Analysis of extracellular vesicle DNA at the single‐vesicle level by nano‐flow cytometry. Journal of Extracellular Vesicles, 11, e12206. 35373518 10.1002/jev2.12206 PMC8977970

[jev212299-bib-0088] Lobb, R. J. , Becker, M. , Wen, S. W. , Wong, C. S. F. , Wiegmans, A. P. , Leimgruber, A. , & Möller, A. (2015). Optimized exosome isolation protocol for cell culture supernatant and human plasma. Journal of Extracellular Vesicles, 4, 27031. 26194179 10.3402/jev.v4.27031 PMC4507751

[jev212299-bib-0089] Lötvall, J. , Hill, A. F. , Hochberg, F. , Buzás, E. I. , Di Vizio, D. , Gardiner, C. , Gho, Y. S. , Kurochkin, I. V. , Mathivanan, S. , Quesenberry, P. , Sahoo, S. , Tahara, H. , Wauben, M. H. , Witwer, K. W. , & Théry, C. (2014). Minimal experimental requirements for definition of extracellular vesicles and their functions: A position statement from the international society for extracellular vesicles. Journal of Extracellular Vesicles, 3, 26913. 25536934 10.3402/jev.v3.26913 PMC4275645

[jev212299-bib-0090] Lozano‐Andrés, E. , Libregts, S. F. , Toribio, V. , Royo, F. , Morales, S. , López‐Martín, S. , Valés‐Gómez, M. , Reyburn, H. T. , Falcón‐Pérez, J. M. , Wauben, M. H. , Soto, M. , & Yáñez‐Mó, M. (2019). Tetraspanin‐decorated extracellular vesicle‐mimetics as a novel adaptable reference material. Journal of Extracellular Vesicles, 8, 1573052. 30863514 10.1080/20013078.2019.1573052 PMC6407598

[jev212299-bib-0091] Lubart, Q. , Hannestad, J. K. , Pace, H. , Fjällborg, D. , Westerlund, F. , Esbjörner, E. K. , & Bally, M. (2020). Lipid vesicle composition influences the incorporation and fluorescence properties of the lipophilic sulphonated carbocyanine dye SP‐DiO. Physical Chemistry Chemical Physics, 22, 8781–8790. 32285050 10.1039/c9cp04158c

[jev212299-bib-0092] Ludwig, A.‐K. , De Miroschedji, K. , Doeppner, T. R. , Börger, V. , Ruesing, J. , Rebmann, V. , Durst, S. , Jansen, S. , Bremer, M. , Behrmann, E. , Singer, B. B. , Jastrow, H. , Kuhlmann, J. D. , El Magraoui, F. , Meyer, H. E. , Hermann, D. M. , Opalka, B. , Raunser, S. , Epple, M. , … Giebel, B. (2018). Precipitation with polyethylene glycol followed by washing and pelleting by ultracentrifugation enriches extracellular vesicles from tissue culture supernatants in small and large scales. Journal of Extracellular Vesicles, 7, 1528109. 30357008 10.1080/20013078.2018.1528109 PMC6197019

[jev212299-bib-0093] Maltsev, V. P. , Hoekstra, A. G. , & Yurkin, M. A. (2011). Optics of white blood cells: Optical models, simulations, and experiments. In: Advanced optical flow cytometry: Methods and Disease diagnoses, V. V. Tuchin (ed.) pp. 63–93.

[jev212299-bib-0094] Mateescu, B. , Kowal, E. J. K. , van Balkom, B. W. M. , Bartel, S. , Bhattacharyya, S. N. , Buzás, E. I. , Buck, A. H. , de Candia, P. , Chow, F. W. N. , Das, S. , Driedonks, T. A. P. , Fernández‐Messina, L. , Haderk, F. , Hill, A. F. , Jones, J. C. , Van Keuren‐Jensen, K. R. , Lai, C. P. , Lässer, C. , di Liegro, I. , … Nolte‐‘t Hoen, E. N. M. (2017). Obstacles and opportunities in the functional analysis of extracellular vesicle RNA–An ISEV position paper. Journal of Extracellular Vesicles, 6, 1286095. 28326170 10.1080/20013078.2017.1286095 PMC5345583

[jev212299-bib-0095] Matyus, S. P. , Braun, P. J. , Wolak‐Dinsmore, J. , Saenger, A. K. , Jeyarajah, E. J. , Shalaurova, I. , Warner, S. M. , Fischer, T. J. , & Connelly, M. A. (2015). HDL particle number measured on the Vantera®, the first clinical NMR analyzer. Clinical Biochemistry, 48, 148. 25438074 10.1016/j.clinbiochem.2014.11.017

[jev212299-bib-0096] McCarthy, D. A. (2007). Fluorochromes and fluorescence, in flow cytometry: Principles and applications, M. G. Macey (ed.), pp. 59–112.

[jev212299-bib-0097] McKinnon, K. M. (2018). Flow cytometry: An overview. Current Protocols in Immunology, 120, 1. 10.1002/cpim.40 PMC5939936 29512141

[jev212299-bib-0098] Meskas, J. , Wang, S. , & Brinkman, R. R. (2020). FlowCut ‐ An R package for precise and accurate automated removal of outlier events and flagging of files based on time versus fluorescence analysis. bioRxiv, 2020.04.23.058545. 10.1002/cyto.a.24670 PMC9823164 35796000

[jev212299-bib-0099] Mitra, K. , Ubarretxena‐Belandia, I. , Taguchi, T. , Warren, G. , & Engelman, D. M. (2004). Modulation of the bilayer thickness of exocytic pathway membranes by membrane proteins rather than cholesterol. Proceedings of the National Academy of Sciences, 101, 4083–4088. 10.1073/pnas.0307332101 PMC384699 15016920

[jev212299-bib-0100] Mol, E. A. , Goumans, M.‐J. , Doevendans, P. A. , Sluijter, J. P. G. , & Vader, P. (2017). Higher functionality of extracellular vesicles isolated using size‐exclusion chromatography compared to ultracentrifugation. Nanomedicine: Nanotechnology, Biology and Medicine, 13, 2061. 28365418 10.1016/j.nano.2017.03.011

[jev212299-bib-0101] Monaco, G. , Chen, H. , Poidinger, M. , Chen, J. , de Magalhães, J. P. , & Larbi, A. (2016). FlowAI: Automatic and interactive anomaly discerning tools for flow cytometry data. Bioinformatics, 32, 2473. 27153628 10.1093/bioinformatics/btw191

[jev212299-bib-0102] Monguió‐Tortajada, M. , Gálvez‐Montón, C. , Bayes‐Genis, A. , Roura, S. , & Borràs, F. E. (2019). Extracellular vesicle isolation methods: Rising impact of size‐exclusion chromatography. Cellular and Molecular Life Sciences, 76, 2369–2382. 30891621 10.1007/s00018-019-03071-y PMC11105396

[jev212299-bib-0103] Monici, M. (2005). Cell and tissue autofluorescence research and diagnostic applications. Biotechnology Annual Review, 11, 227. 10.1016/S1387-2656(05)11007-2 16216779

[jev212299-bib-0104] Morales‐Kastresana, A. , Musich, T. A. , Welsh, J. A. , Telford, W. , Demberg, T. , Wood, J. C. S. , Bigos, M. , Ross, C. D. , Kachynski, A. , Dean, A. , Felton, E. J. , Van Dyke, J. , Tigges, J. , Toxavidis, V. , Parks, D. R. , Overton, W. R. , Kesarwala, A. H. , Freeman, G. J. , Rosner, A. , … Jones, J. C. (2019). High‐fidelity detection and sorting of nanoscale vesicles in viral disease and cancer. Journal of Extracellular Vesicles, 8, 1597603. 31258878 10.1080/20013078.2019.1597603 PMC6586126

[jev212299-bib-0105] Morales‐Kastresana, A. , Telford, B. , Musich, T. A. , McKinnon, K. , Clayborne, C. , Braig, Z. , Rosner, A. , Demberg, T. , Watson, D. C. , Karpova, T. S. , Freeman, G. J. , DeKruyff, R. H. , Pavlakis, G. N. , Terabe, M. , Robert‐Guroff, M. , Berzofsky, J. A. , & Jones, J. C. (2017). Labeling extracellular vesicles for nanoscale flow cytometry. Scientific Reports, 7, 1878. 28500324 10.1038/s41598-017-01731-2 PMC5431945

[jev212299-bib-0106] Morales‐Kastresana, A. , Welsh, J. A. , & Jones, J. C. (2020). Detection and sorting of extracellular vesicles and viruses using nanoFACS. Current Protocols in Cytometry, 95, e81. 33332760 10.1002/cpcy.81 PMC8443073

[jev212299-bib-0107] Nguyen, R. , Perfetto, S. , Mahnke, Y. D. , Chattopadhyay, P. , & Roederer, M. (2013). Quantifying spillover spreading for comparing instrument performance and aiding in multicolor panel design. Cytometry Part A, 83, 306. 10.1002/cyto.a.22251 PMC3678531 23389989

[jev212299-bib-0108] Palviainen, M. , Saraswat, M. , Varga, Z. , Kitka, D. , Neuvonen, M. , Puhka, M. , Joenväärä, S. , Renkonen, R. , Nieuwland, R. , Takatalo, M. , & Siljander, P. R. M. (2020). Extracellular vesicles from human plasma and serum are carriers of extravesicular cargo ‐ Implications for biomarker discovery. PLoS ONE, 15, e0236439. 32813744 10.1371/journal.pone.0236439 PMC7446890

[jev212299-bib-0109] Parks, D. R. , El Khettabi, F. , Chase, E. , Hoffman, R. A. , Perfetto, S. P. , Spidlen, J. , Wood, J. C. S. , Moore, W. A. , & Brinkman, R. R. (2017). Evaluating flow cytometer performance with weighted quadratic least squares analysis of LED and multi‐level bead data. Cytometry Part A, 91, 232. 10.1002/cyto.a.23052 PMC5483398 28160404

[jev212299-bib-0110] Perissinotto, F. , Rondelli, V. , Senigagliesi, B. , Brocca, P. , Almásy, L. , Bottyán, L. , Merkel, D. G. , Amenitsch, H. , Sartori, B. , Pachler, K. , Mayr, M. , Gimona, M. , Rohde, E. , Casalis, L. , & Parisse, P. (2021). Structural insights into fusion mechanisms of small extracellular vesicles with model plasma membranes. Nanoscale, 13, 5224–5233. 33687046 10.1039/d0nr09075a

[jev212299-bib-0111] Pinkel, D. , Dean, P. , Lake, S. , Peters, D. , Mendelsohn, M. , Gray, J. , Van Dilla, M. , & Gledhill, B. (1979). Flow cytometry of mammalian sperm: Progress in DNA and morphology measurement. Journal of Histochemistry and Cytochemistry, 27, 353. 86565 10.1177/27.1.86565

[jev212299-bib-0112] Poisson, S. D. (1837). Recherches Sur La Probabilité Des Jugements En Matière Criminelle et En Matière Civile (Bachelier).

[jev212299-bib-0113] Polyanskiy, M. N. Refractive Index Database. https://refractiveindex.info/. Accessed September 2, 2022.

[jev212299-bib-0114] Pospichalova, V. , Svoboda, J. , Dave, Z. , Kotrbova, A. , Kaiser, K. , Klemova, D. , Ilkovics, L. , Hampl, A. , Crha, I. , Jandakova, E. , Minar, L. , Weinberger, V. , & Bryja, V. (2015). Simplified protocol for flow cytometry analysis of fluorescently labeled exosomes and microvesicles using dedicated flow cytometer. Journal of Extracellular Vesicles, 4, 25530. 25833224 10.3402/jev.v4.25530 PMC4382613

[jev212299-bib-0115] Rikkert, L. G. , van der Pol, E. , van Leeuwen, T. G. , Nieuwland, R. , & Coumans, F. A. W. (2018). Centrifugation affects the purity of liquid biopsy‐based tumor biomarkers. Cytometry Part A, 93, 1207. 10.1002/cyto.a.23641 PMC6590195 30551256

[jev212299-bib-0116] Rood, I. M. , Deegens, J. K. J. , Merchant, M. L. , Tamboer, W. P. M. , Wilkey, D. W. , Wetzels, J. F. M. , & Klein, J. B. (2010). Comparison of three methods for isolation of urinary microvesicles to identify biomarkers of nephrotic syndrome. Kidney International, 78, 810. 20686450 10.1038/ki.2010.262

[jev212299-bib-0117] Saleh, B. E. A. , & Teich, M. C. (1991). Fundamentals of photonics. 1st ed.

[jev212299-bib-0118] Saper, C. B. (2009). A guide to the perplexed on the specificity of antibodies. Journal of Histochemistry and Cytochemistry, 57, 1. 18854594 10.1369/jhc.2008.952770 PMC2605712

[jev212299-bib-0119] Schroeder, R. , London, E. , & Brown, D. (1994). Interactions between saturated acyl chains confer detergent resistance on lipids and Glycosylphosphatidylinositol (GPI)‐anchored proteins: GPI‐anchored proteins in liposomes and cells show similar behavior. Proceedings of the National Academy of Sciences, 91, 12130. 10.1073/pnas.91.25.12130 PMC45390 7991596

[jev212299-bib-0120] Schwartz, A. , Gaigalas, A. K. , Wang, L. , Marti, G. E. , Vogt, R. F. , & Fernandez‐Repollet, E. (2004). Formalization of the MESF unit of fluorescence intensity. Cytometry Part B, 57, 1. 10.1002/cyto.b.10066 14696057

[jev212299-bib-0121] Schwarze, W. , Bernhardt, R. , Jänig, G.‐R. , & Ruckpaul, K. (1983). Fluorescent energy transfer measurements on fluorescein isothiocyanate modified cytochrome P‐450 LM2. Biochemical and Biophysical Research Communications, 113, 353. 6407482 10.1016/0006-291x(83)90473-4

[jev212299-bib-0122] Seddon, A. M. , Curnow, P. , & Booth, P. J. (2004). Membrane proteins, lipids and detergents: Not just a soap opera. Biochimica et Biophysica Acta ‐ Biomembranes, 1666, 105–117. 10.1016/j.bbamem.2004.04.011 15519311

[jev212299-bib-0123] Seidel, C. A. M. , Schulz, A. , & Sauer, M. H. M. (1996). Nucleobase‐specific quenching of fluorescent dyes. 1. Nucleobase one‐electron redox potentials and their correlation with static and dynamic quenching efficiencies. Journal of Physical Chemistry, 100, 5541.

[jev212299-bib-0124] Shapiro, H. M. (2003). Practical flow cytometry. 4th ed.

[jev212299-bib-0125] Shen, W. , Guo, K. , Adkins, G. B. , Jiang, Q. , Liu, Y. , Sedano, S. , Duan, Y. , Yan, W. , Wang, S. E. , Bergersen, K. , Worth, D. , Wilson, E. H. , & Zhong, W. A. (2018). Single Extracellular Vesicle (Ev) flow cytometry approach to reveal Ev heterogeneity. Angewandte Chemie International Edition, 57, 15675–15680. 30291794 10.1002/anie.201806901 PMC6246790

[jev212299-bib-0126] Snow, C. (2004). Flow cytometer electronics. Cytometry Part A, 57, 63. 10.1002/cyto.a.10120 14750126

[jev212299-bib-0127] Sódar, B. W. , Kittel, Á. , Pálóczi, K. , Vukman, K. V. , Osteikoetxea, X. , Szabó‐Taylor, K. , Németh, A. , Sperlágh, B. , Baranyai, T. , Giricz, Z. , Wiener, Z. , Turiák, L. , Drahos, L. , Pállinger, É. , Vékey, K. , Ferdinandy, P. , Falus, A. , & Buzás, E. I. (2016). Low‐density lipoprotein mimics blood plasma‐derived exosomes and microvesicles during isolation and detection. Scientific Reports, 6, 24316. 27087061 10.1038/srep24316 PMC4834552

[jev212299-bib-0128] Steen, H. B. (1992). Noise, sensitivity, and resolution of flow cytometers. Cytometry, 13, 822. 1458999 10.1002/cyto.990130804

[jev212299-bib-0129] Stoner, S. A. , Duggan, E. , Condello, D. , Guerrero, A. , Turk, J. R. , Narayanan, P. K. , & Nolan, J. P. (2016). High sensitivity flow cytometry of membrane vesicles. Cytometry Part A, 89, 196. 10.1002/cyto.a.22787 26484737

[jev212299-bib-0130] Student . (1907). On the error of counting with a haemacytometer. Biometrika, 351. http://www.medicine.mcgill.ca/epidemiology/hanley/c634/rates/HaemacytometerStudentBka1907.pdf

[jev212299-bib-0131] Sverdlov, E. D. (2012). Amedeo Avogadro's Cry: What is 1 microgram of exosomes? Bioessays, 34, 873. 22815202 10.1002/bies.201200045

[jev212299-bib-0132] SzöllHosi, J. , Damjanovich, S. , Nagy, P. , Vereb, G. , & Mátyus, L. (2006). Principles of resonance energy transfer. Current Protocols in Cytometry, 38, 1. 10.1002/0471142956.cy0112s38 18770831

[jev212299-bib-0133] Tan, W. , Wang, K. , & Drake, T. J. (2004). Molecular Beacons. Current Opinion in Chemical Biology, 8, 547. 15450499 10.1016/j.cbpa.2004.08.010

[jev212299-bib-0134] Tang, V. A. , Fritzsche, A. K. , Renner, T. M. , Burger, D. , Lannigan, J. A. , Brittain, G. C. , Ouellet, C. V. , Van der Pol, E. , & Langlois, M.‐A. (2019). Engineered retroviruses as fluorescent biological reference particles for nanoscale flow cytometry. bioRxiv, 614461.

[jev212299-bib-0135] Tertel, T. , Bremer, M. , Maire, C. , Lamszus, K. , Peine, S. , Jawad, R. , Andaloussi, S. E. L. , Giebel, B. , Ricklefs, F. L. , & Görgens, A. (2020). High‐resolution imaging flow cytometry reveals impact of incubation temperature on labeling of extracellular vesicles with antibodies. Cytometry Part A, 97, 602. 10.1002/cyto.a.24034 32415810

[jev212299-bib-0136] Théry, C. , Witwer, K. W. , Aikawa, E. , Alcaraz, M. J. , Anderson, J. D. , Andriantsitohaina, R. , Antoniou, A. , Arab, T. , Archer, F. , Atkin‐Smith, G. K. , Ayre, D. C. , Bach, J.‐M. , Bachurski, D. , Baharvand, H. , Balaj, L. , Baldacchino, S. , Bauer, N. N. , Baxter, A. A. , Bebawy, M. , … Zuba‐Surma, E. K. (2018). Minimal Information for Studies of Extracellular Vesicles 2018 (MISEV2018): A position statement of the international society for extracellular vesicles and update of the MISEV2014 guidelines. Journal of Extracellular Vesicles, 7, 1535750. 30637094 10.1080/20013078.2018.1535750 PMC6322352

[jev212299-bib-0137] Tian, Y. , Gong, M. , Hu, Y. , Liu, H. , Zhang, W. , Zhang, M. , Hu, X. , Aubert, D. , Zhu, S. , Wu, L. , & Yan, X. (2020). Quality and efficiency assessment of six extracellular vesicle isolation methods by nano‐flow cytometry. Journal of Extracellular Vesicles, 9, 1697028. 31839906 10.1080/20013078.2019.1697028 PMC6896440

[jev212299-bib-0138] Valkenburg, J. A. , & Woldringh, C. L. (1984). Phase separation between nucleoid and cytoplasm in Escherichia coli as defined by immersive refractometry. Journal of Bacteriology, 160, 1151. 6389508 10.1128/jb.160.3.1151-1157.1984 PMC215833

[jev212299-bib-0139] van den Engh, G. , & Farmer, C. (1992). Photo‐bleaching and photon saturation in flow cytometry. Cytometry Part A, 13, 669. 10.1002/cyto.990130702 1280553

[jev212299-bib-0140] van der Pol, E. , Böing, A. N. , Harrison, P. , Sturk, A. , & Nieuwland, R. (2012). Classification, functions, and clinical relevance of extracellular vesicles. Pharmacological Reviews, 64, 676. 22722893 10.1124/pr.112.005983

[jev212299-bib-0141] van der Pol, E. , Coumans, F. A. W. , Grootemaat, A. E. , Gardiner, C. , Sargent, I. L. , Harrison, P. , Sturk, A. , van Leeuwen, T. G. , & Nieuwland, R. (2014). Particle size distribution of exosomes and microvesicles determined by transmission electron microscopy, flow cytometry, nanoparticle tracking analysis, and resistive pulse sensing. Journal of Thrombosis and Haemostasis, 12, 1182. 24818656 10.1111/jth.12602

[jev212299-bib-0142] van der Pol, E. , Coumans, F. A. W. , Sturk, A. , Nieuwland, R. , & Van Leeuwen, T. G. (2014). Refractive index determination of nanoparticles in suspension using nanoparticle tracking analysis. Nano Letters, 14, 6195. 25256919 10.1021/nl503371p

[jev212299-bib-0143] van der Pol, E. , Coumans, F. A. W. , Varga, Z. , Krumrey, M. , & Nieuwland, R. (2013). Innovation in detection of microparticles and exosomes. Journal of Thrombosis and Haemostasis, 11, 36. 23809109 10.1111/jth.12254

[jev212299-bib-0144] van der Pol, E. , de Rond, L. , Coumans, F. A. W. , Gool, E. L. , Böing, A. N. , Sturk, A. , Nieuwland, R. , & van Leeuwen, T. G. (2018). Absolute sizing and label‐free identification of extracellular vesicles by flow cytometry. Nanomedicine: Nanotechnology, Biology and Medicine, 14, 801. 29307842 10.1016/j.nano.2017.12.012

[jev212299-bib-0145] van der Pol, E. , Hoekstra, A. G. , Sturk, A. , Otto, C. , van Leeuwen, T. G. , & Nieuwland, R. (2010). Optical and non‐optical methods for detection and characterization of microparticles and exosomes. Journal of Thrombosis and Haemostasis, 8, 2596. 20880256 10.1111/j.1538-7836.2010.04074.x

[jev212299-bib-0146] van der Pol, E. , Sturk, A. , van Leeuwen, T. G. , Nieuwland, R. , Coumans, F. A. W. , & Group, I.‐S.‐V. W. (2018). Standardization of extracellular vesicle measurements by flow cytometry through vesicle diameter approximation. Journal of Thrombosis and Haemostasis, 16, 1236. 29575716 10.1111/jth.14009

[jev212299-bib-0147] van der Pol, E. , van Gemert, M. J. C. , Sturk, A. , Nieuwland, R. , & van Leeuwen, T. G. (2012). Single vs. swarm detection of microparticles and exosomes by flow cytometry. Journal of Thrombosis and Haemostasis, 10, 919. 22394434 10.1111/j.1538-7836.2012.04683.x

[jev212299-bib-0148] van der Pol, E. , van Leeuwen, T. G. , & Yan, X. (2021). Misinterpretation of solid sphere equivalent refractive index measurements and smallest detectable diameters of extracellular vesicles by flow cytometry. Scientific Reports, 11, 24151. 34921157 10.1038/s41598-021-03015-2 PMC8683472

[jev212299-bib-0149] van der Pol, E. , Welsh, J. A. , & Nieuwland, R. (2022). Minimum information to report about a flow cytometry experiment on extracellular vesicles: Communication from the ISTH SSC subcommittee on vascular biology. Journal of Thrombosis and Haemostasis, 20, 245. 34637195 10.1111/jth.15540 PMC8729195

[jev212299-bib-0150] van der Vlist, E. J. , Nolte‐’t Hoen, E. N. M. , Stoorvogel, W. , Arkesteijn, G. J. A. , & Wauben, M. H. M. (2012). Fluorescent labeling of nano‐sized vesicles released by cells and subsequent quantitative and qualitative analysis by high‐resolution flow cytometry. Nature Protocols, 7, 1311. 22722367 10.1038/nprot.2012.065

[jev212299-bib-0151] Van Deun, J. , Mestdagh, P. , Agostinis, P. , Akay, Ö. , Anand, S. , Anckaert, J. , Martinez, Z. A. , Baetens, T. , Beghein, E. , Bertier, L. , Berx, G. , Boere, J. , Boukouris, S. , Bremer, M. , Buschmann, D. , Byrd, J. B. , Casert, C. , Cheng, L. , … Hendrix, A. (2017). EV‐TRACK: Transparent reporting and centralizing knowledge in extracellular vesicle research. Nature Methods, 14, 228–232. 28245209 10.1038/nmeth.4185

[jev212299-bib-0152] van Herwijnen, M. J. C. , Zonneveld, M. I. , Goerdayal, S. , Nolte, E. N. M. , Garssen, J. , Stahl, B. , Altelaar, A. F. M. , Redegeld, F. A. , & Wauben, M. H. M. (2016). Comprehensive proteomic analysis of human milk‐derived extracellular vesicles unveils a novel functional proteome distinct from other milk components. Molecular & Cellular Proteomics, 15, 3412. 27601599 10.1074/mcp.M116.060426 PMC5098039

[jev212299-bib-0153] van Manen, H.‐J. , Verkuijlen, P. , Wittendorp, P. , Subramaniam, V. , van den Berg, T. K. , Roos, D. , & Otto, C. (2008). Refractive index sensing of green fluorescent proteins in living cells using fluorescence lifetime imaging microscopy. Biophysical Journal, 94, L67–L69. 18223002 10.1529/biophysj.107.127837 PMC2275685

[jev212299-bib-0154] Van Oss, C. J. , Good, R. J. , & Chaudhury, M. K. (1986). Nature of the antigen‐antibody interaction: Primary and secondary bonds: Optimal conditions for association and dissociation. Journal of Chromatography B, 376, 111. 3711190

[jev212299-bib-0155] Vogel, R. , Savage, J. , Muzard, J. , Della Camera, G. , Vella, G. , Law, A. , Marchioni, M. , Mehn, D. , Geiss, O. , Peacock, B. , Aubert, D. , Calzolai, L. , Caputo, F. , & Prina‐Mello, A. (2021). Measuring particle concentration of multimodal synthetic reference materials and extracellular vesicles with orthogonal techniques: Who is up to the challenge? Journal of Extracellular Vesicles, 10, e12052. 33473263 10.1002/jev2.12052 PMC7804049

[jev212299-bib-0156] Vogel, S. S. , van der Meer, B. W. , & Blank, P. S. (2014). Estimating the distance separating fluorescent protein FRET pairs. Methods, 66, 131. 23811334 10.1016/j.ymeth.2013.06.021 PMC3964137

[jev212299-bib-0157] Vorselen, D. , van Dommelen, S. M. , Sorkin, R. , Piontek, M. C. , Schiller, J. , Döpp, S. T. , Kooijmans, S. A. A. , van Oirschot, B. A. , Versluijs, B. A. , Bierings, M. B. , van Wijk, R. , Schiffelers, R. M. , Wuite, G. J. L. , & Roos, W. H. (2018). The fluid membrane determines mechanics of erythrocyte extracellular vesicles and is softened in hereditary spherocytosis. Nature Communications, 9, 1. 10.1038/s41467-018-07445-x PMC6251882 30470753

[jev212299-bib-0158] Wang, L. , & Hoffman, R. A. (2017). Standardization, calibration, and control in flow cytometry. Current Protocols in Cytometry, 79, 1. 10.1002/cpcy.14 28055116

[jev212299-bib-0160] Weller, M. G. (2018). Ten basic rules of antibody validation. Analytical Chemistry Insights, 13, 1. 10.1177/1177390118757462 PMC5813849 29467569

[jev212299-bib-0161] Welsh, J. A. , Horak, P. , Wilkinson, J. S. , Ford, V. J. , Jones, J. C. , Smith, D. , Holloway, J. A. , & Englyst, N. A. (2020). FCMPASS software aids extracellular vesicle light scatter standardization. Cytometry Part A, 97, 569. 10.1002/cyto.a.23782 PMC7061335 31250561

[jev212299-bib-0162] Welsh, J. A. , Jones, J. C. , & Tang, V. A. (2020). Fluorescence and light scatter calibration allow comparisons of small particle data in standard units across different flow cytometry platforms and detector settings. Cytometry Part A, 97, 592. 10.1002/cyto.a.24029 PMC8482305 32476280

[jev212299-bib-0163] Welsh, J. A. , Pol, E. , Bettin, B. A. , Carter, D. R. F. , Hendrix, A. , Lenassi, M. , Langlois, M. , Llorente, A. , Nes, A. S. , Nieuwland, R. , Tang, V. , Wang, L. , Witwer, K. W. , & Jones, J. C. (2020). Towards defining reference materials for measuring extracellular vesicle refractive index, epitope abundance, size and concentration. Journal of Extracellular Vesicles, 9, 1816641. 33062218 10.1080/20013078.2020.1816641 PMC7534292

[jev212299-bib-0164] Welsh, J. A. , Tang, V. A. , van der Pol, E. , & Görgens, A. (2021). MIFlowCyt‐EV: The next chapter in the reporting and reliability of single extracellular vesicle flow cytometry experiments. Cytometry Part A, 99, 365. 10.1002/cyto.a.24268 33200505

[jev212299-bib-0165] Welsh, J. A. , Van Der Pol, E. , Arkesteijn, G. J. A. , Bremer, M. , Brisson, A. , Coumans, F. , Dignat‐George, F. , Duggan, E. , Ghiran, I. , Giebel, B. , Görgens, A. , Hendrix, A. I. , Lacroix, R. , Lannigan, J. , Libregts, S. F. W. M. , Lozano‐Andrés, E. , Morales‐Kastresana, A. , Robert, S. , De Rond, L. , … Jones, J. C. (2020). MIFlowCyt‐EV: A framework for standardized reporting of extracellular vesicle flow cytometry experiments. Journal of Extracellular vesicles, 9, 1713526. 32128070 10.1080/20013078.2020.1713526 PMC7034442

[jev212299-bib-0166] Wiklander, O. P. B. , Bostancioglu, R. B. , Welsh, J. A. , Zickler, A. M. , Murke, F. , Corso, G. , Felldin, U. , Hagey, D. W. , Evertsson, B. , Liang, X.‐M. , Gustafsson, M. O. , Mohammad, D. K. , Wiek, C. , Hanenberg, H. , Bremer, M. , Gupta, D. , Björnstedt, M. , Giebel, B. , Nordin, J. Z. , … Görgens, A. (2018). Systematic methodological evaluation of a multiplex bead‐based flow cytometry assay for detection of extracellular vesicle surface signatures. Frontiers in Immunology, 9, 1326. 29951064 10.3389/fimmu.2018.01326 PMC6008374

[jev212299-bib-0167] Wood, J. C. S. (1998). Fundamental flow cytometer properties governing sensitivity and resolution. Cytometry, 33, 260. 9773889

[jev212299-bib-0168] Xie, H. (2017). Validated Antibody Database (VAD): A database of antibodies curated from publications. FASEB Journal, 31, 983.

[jev212299-bib-0169] Xiong, F. , & Tanik, M. M. (2015). Deciphering flow cytometry electronics. In SoutheastCon 2015. Fort Lauderdale, USA. pp. 1–5.

[jev212299-bib-0170] Yuana, Y. , Böing, A. N. , Grootemaat, A. E. , van der Pol, E. , Hau, C. M. , Cizmar, P. , Buhr, E. , Sturk, A. , & Nieuwland, R. (2015). Handling and storage of human body fluids for analysis of extracellular vesicles. Journal of Extracellular Vesicles, 4, 29260. 26563735 10.3402/jev.v4.29260 PMC4643195

[jev212299-bib-0171] Zhu, S. , Ma, L. , Wang, S. , Chen, C. , Zhang, W. , Yang, L. , Hang, W. , Nolan, J. P. , Wu, L. , & Yan, X. (2014). Light‐scattering detection below the level of single fluorescent molecules for high‐resolution characterization of functional nanoparticles. ACS Nano, 8, 10998. 25300001 10.1021/nn505162u PMC4212780

